# Driver mutations of intrahepatic cholangiocarcinoma shape clinically relevant genomic clusters with distinct molecular features and therapeutic vulnerabilities

**DOI:** 10.7150/thno.63417

**Published:** 2022-01-01

**Authors:** Xiang-Yu Wang, Wen-Wei Zhu, Zheng Wang, Jian-Bo Huang, Sheng-Hao Wang, Fu-Mao Bai, Tian-En Li, Ying Zhu, Jing Zhao, Xin Yang, Lu Lu, Ju-Bo Zhang, Hu-Liang Jia, Qiong-Zhu Dong, Jin-Hong Chen, Jesper B. Andersen, Dan Ye, Lun-Xiu Qin

**Affiliations:** 1 Department of General Surgery, Huashan Hospital, Fudan University, Shanghai, China; 2 Cancer Metastasis Institute, Fudan University, Shanghai, China; 3 Department of Laboratory Medicine, The First Affiliated Hospital of Wenzhou Medical University, Wenzhou, China; 4 Department of Infectious Diseases, Huashan Hospital, Fudan University, Shanghai, China; 5 Biotech Research and Innovation Centre (BRIC), Department of Health and Medical Sciences, University of Copenhagen, Copenhagen N, Denmark; 6 Molecular and Cell Biology Lab, Institutes of Biomedical Sciences, and the Shanghai Key Laboratory of Medical Epigenetics, and the Key Laboratory of Metabolism and Molecular, Ministry of Education, Fudan University, Shanghai, China

**Keywords:** Genome sequencing, Driver mutation, ICC diversity.

## Abstract

**Purpose:** To establish a clinically applicable genomic clustering system, we investigated the interactive landscape of driver mutations in intrahepatic cholangiocarcinoma (ICC).

**Methods:** The genomic data of 1481 ICCs from diverse populations was analyzed to investigate the pair-wise co-occurrences or mutual exclusivities among recurrent driver mutations. Clinicopathological features and outcomes were compared among different clusters. Gene expression and DNA methylation profiling datasets were analyzed to investigate the molecular distinctions among mutational clusters. ICC cell lines with different gene mutation backgrounds were used to evaluate the cluster specific biological behaviors and drug sensitivities.

**Results:** Statistically significant mutation-pairs were identified across 21 combinations of genes. Seven most recurrent driver mutations (*TP53*, *KRAS*, *SMAD4*, *IDH1*/2, *FGFR2-fus* and *BAP1*) showed pair-wise co-occurrences or mutual exclusivities and could aggregate into three genetic clusters: Cluster1: represented by tripartite interaction of *KRAS*, *TP53* and *SMAD4* mutations, exhibited large bile duct histological phenotype with high CA19-9 level and dismal prognosis; Cluster2: co-association of *IDH*/*BAP1* or *FGFR2-fus*/*BAP1* mutation, was characterized by small bile duct phenotype, low CA19-9 level and optimal prognosis; Cluster3: mutation-free ICC cases with intermediate clinicopathological features. These clusters showed distinct molecular traits, biological behaviors and responses to therapeutic drugs. Finally, we identified S100P and KRT17 as “cluster-specific”, “lineage-dictating” and “prognosis-related” biomarkers, which in combination with CA19-9 could well stratify Cluster3 ICCs into two biologically and clinically distinct subtypes.

**Conclusions:** This clinically applicable clustering system can be instructive to ICC prognostic stratification, molecular classification, and therapeutic optimization.

## Introduction

Intrahepatic cholangiocarcinoma (ICC) is a highly intractable biliary tract cancer (BTC) originating from the epithelial cells of intrahepatic biliary trees, which is anatomically distinguished from extrahepatic cholangiocarcinoma (ECC). ICC also remains as the second most common primary liver cancer (PLC) following hepatocellular carcinoma (HCC) 1, 2. Although ICC is more prevalent in eastern and southeastern Asian countries, such as China and Thailand 3, its incidence and mortality are increasing globally in recent decades 4-6. With highly invasive and metastatic phenotype, ICC patients are mostly diagnosed at advanced stages with dismal outcome despite receiving standard chemotherapy. Surgical resection is the only curative treatment strategy for early stage ICCs, but a high rate of loco-regional recurrence and distant metastasis also constrain their long-term survival 7.

Various etiological factors, including viral hepatitis infection, metabolic liver disease, type 2 diabetes mellitus (T2DM), primary sclerosing cholangitis (PSC), liver fluke infection and hepatolithiasis, are known to be involved in the heterogeneous tumorigenic process of ICC 8, 9. And different cellular origins further contribute to its high histological complexity. Supporting this notion, some ICCs originate from mature cholangiocytes of large bile ducts and display major similarities to ECC, while the other subtypes originate from small bile ductules or progenitor cells in the Canal of Hering and even show some traits of HCC 2. The diversities of etiologies and cellular origins indicate the complicated mechanisms during ICC tumorigenesis. The development of an effective classification system for the clinical practice of ICC is thus urgently needed.

Based on comprehensive genomic characterization, the mutational landscape of ICC is emerging in recent years with the discovery of multiple driver mutations, which is different from that of ECC or HCC, and shows higher diversity 10-25. In addition, specific etiological and ethnical factors also exacerbate the discrepancy between Eastern and Western countries. For example, *TP53, KRAS* and *SMAD4* mutations have been identified as the most recurrent mutations in ICCs from Eastern countries with the prevalence of liver fluke, viral hepatitis and hepatolithiasis 11, 13, 16, 19-21, 23; in contrast, mutations without clear etiological factors, such as *IDH1*/*2, BAP1, PBRM1* mutations and *FGFR2* fusion are more prevalent in Western countries 12, 17, 18, 21, 22, 25. All these factors have inevitably added the difficulty for the establishment of a clinically applicable classification for ICC.

From a genetic perspective, cancer evolves through the emergence and selection of various genetic alterations 26. It has been estimated that average 4 (ranging from 1-10) driver mutations are needed for tumor development under positive selection 27. During this process, gene mutations do not occur randomly. Functionally antagonistic mutations are likely to be selected exclusively of each other, whereas synergistic alterations are frequently co-selected and observed together in certain tumor subtypes. This evidence suggests the existence of an underlying network of functional dependencies between driver mutations with potential biological and clinical impacts. To date, large-scale genetic profiling of human cancers has provided evidence of non-random patterns of co-occurrence and mutual exclusivity among specific oncogenic mutations, which sheds insightful light on cancer evolution 28-30, and can clinically guide molecular classification 31, 32, prognostication 33, 34 and therapeutic stratification 35-38. For ICC, the systematic discovery of driver mutations and their relationships is, however, far from elucidation, which is mainly due to lack of sufficient clinical specimens.

In the present study, we found that seven most recurrent mutations (*TP53*, *KRAS*, *SMAD4*, *IDH1*/2, *FGFR2-fus* and *BAP1*) showed pair-wise co-occurrences or mutual exclusivities in ICC, and could aggregate into three genomic clusters with distinct clinicopathological and molecular features, biological behaviors and therapeutic vulnerabilities. By integrative analysis, we further proposed a clinically and molecularly relevant “seven-mutation and three-marker” based clustering system, which provided framework for a stable, reproducible and clinically applicable ICC clustering strategy.

## Materials and Methods

### ICC patients and genetic mutation data

**Figure 1:**
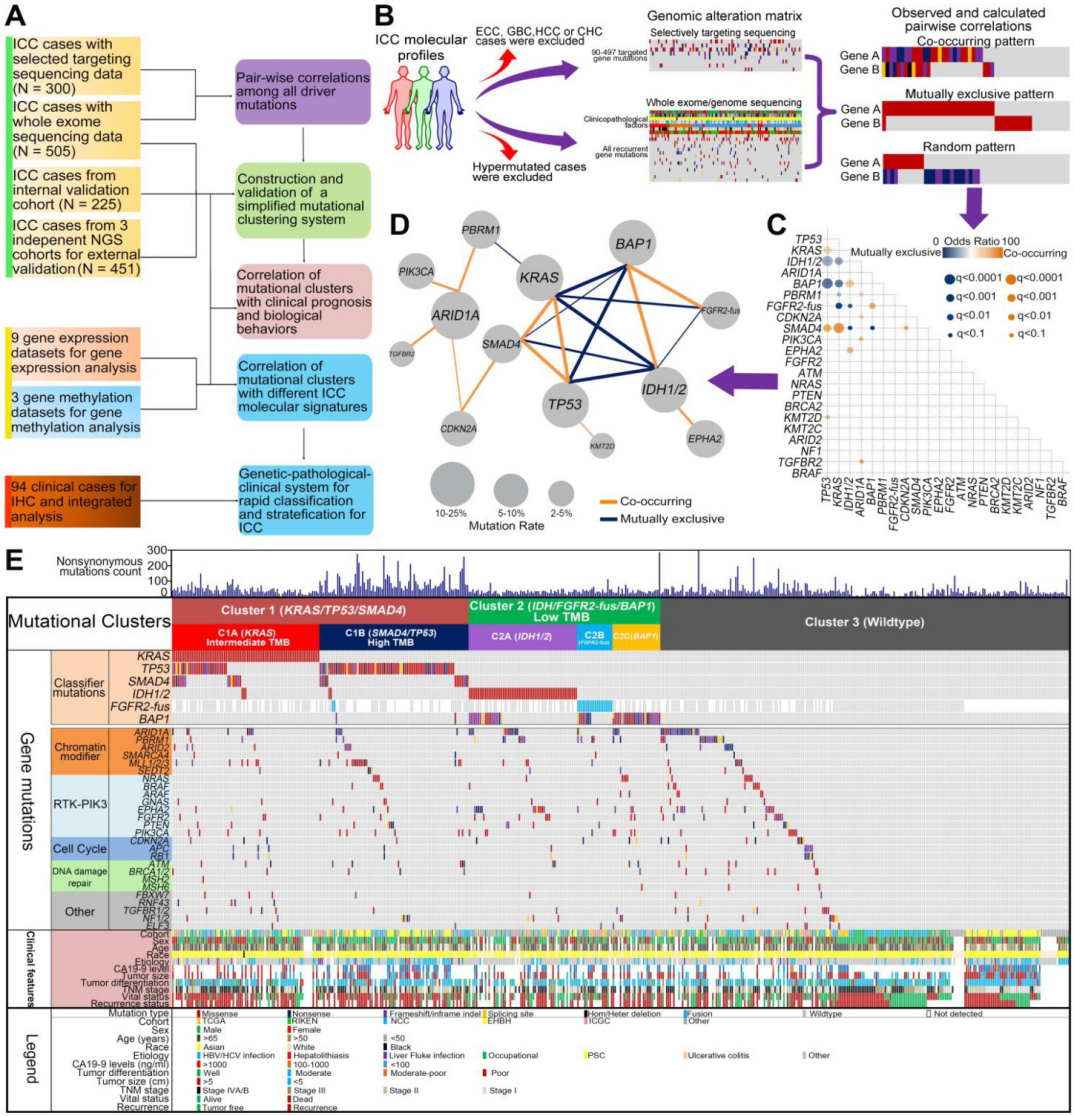
** Co-occurrence and mutual exclusivity analysis of driver gene mutations identified 3 clusters of ICC. (A)** Schematic overview of the study design. **(B)** Schematic of the gene-gene correlation algorithm. **(C)** Correlation between mutations found in 22 genes associated with ICC pathogenesis. Correlation coefficients and associated *q* values are indicated by the size of circles and color gradient as indicated. Because the status of *FGFR2*-fus was not detected for all samples, so the results regarding the correlation of *FGFR2*-fus with other mutations should still be interpreted with caution. **(D)** The co-mutated network modules of the 21 significantly correlated gene mutation pairs. Within the network, the nodes represent mutant genes and the edges between pathways represent their co-mutation relationship. The size of a node is proportional to the mutation rate of this gene. The thickness of an edge is proportional to the significance level (*q* value) of co-mutation between the two genes. **(E)** Construction of 3 mutational clusters based on the co-occurring and mutual exclusivity of driver mutations in WES/WGS cohort including 505 patients. Key clinical characteristics are indicated, including original cohort, age, gender, race, etiology, CA19-9, tumor size, AJCC stage and outcome.

In order to develop a reliable and clinically applicable mutational clustering system for ICC, we conducted a network-based analyses in search for the cooperative or antagonistic mutational pairs, with a discovery and validation design (Figure 1A-B).

The discovery cohort included 805 ICCs from 24 recent publications (2013-2020) with various etiologies and races (Table S1). Any suspected ECC (including perihilar cholangiocarcinoma or distal cholangiocarcinoma), gallbladder cancer (GBC), HCC or combined hepatocellular-cholangiocarcinoma (CHC) cases were excluded from the final analysis. The detection methods for somatic mutations included selected targeting sequencing (STS), whole exome sequencing (WES) and whole genome sequencing (WGS). For STS cohort, only samples with > 50 cancer-related genes detected were included, and samples without detailed information on specific gene mutations were excluded. For WES/WGS cohort, all the mutated genes were collected, and the number of non-synonymous mutations was calculated. More detailed information about sequencing data processing is described in the online supplementary materials and methods. To avoid the undue effects of samples with high mutation burden on subsequent analyses, samples with hyper-mutational phenotype (non-synonymous mutations > 300/sample) in the WES/WGS cohort were also excluded from further analysis. For cases with clinical information available, individual level clinicopathological parameters (including sex, age, CA19-9 level, UICC/AJCC Stage and tumor size) and clinical outcomes (including overall survival and recurrence-free survival) were also acquired from the original publications (Table S2). Finally, genes sequenced in at least 70% of samples with mutation frequency > 2% in the discovery cohort were included for further analyses in search for the cooperative or antagonistic mutational pairs.

Internal validation cohort included 225 ICC patients who underwent surgical resection (n = 217) or tissue biopsy (n = 8) at the authors' institute. Two pathologists independently evaluated hematoxylin and eosin-stained slides and confirmed the diagnosis of ICC. The clinicopathological features were summarized in Table S2. Tumor stage was classified according to the 8th TNM stage of the UICC/AJCC. The mutational status of clustering genes was detected using NGS platform (n = 102) (Table S3) or Sanger sequencing (n = 123). More detailed information about sequencing data processing is described in the online supplementary materials and methods. This study was approved by the Ethics Committee of Huashan Hospital, Fudan University, and all subjects agreed with informed consent to participate in the study.

Two independent ICC cohorts with distinct ethnic backgrounds were recruited for external validation. Cohort 1 including 212 ICCs from Memorial Sloan-Kettering Cancer Center (MSKCC) was from the 'Genomics, Evidence, Neoplasia, Information, Exchange' (GENIE version 3.0.0) project initiated by the American Association for Cancer Research (AACR) 39. For 158 cases in this cohort, individual level clinical and outcome information was download from the cbioportal database (https://www.cbioportal.org/study/summary?id=chol_msk_2018). Cohort 2 including 239 ICC patients mainly from Eastern Asian countries was obtained from the Thailand Initiative in Genomics and Expression Research for Liver Cancer (TIGER-LC) and International Cancer Genome Consortium (ICGC) projects. Their genetic profiles and clinicopathological features were acquired from the original publications (Table S2) 19, 20.

An ECC cohort including 178 ECCs with survival data available from The Cancer Genome Atlas (TCGA) and ICGC projects was used as a control in the subsequent survival analysis (Table S2) 18, 19.

### Gene expression profiling datasets of CCAs

A total of nine gene expression profiling datasets of CCA were enrolled for this study. Various techniques of the expression profiling were applied in the nine cohorts. For further details regarding the included datasets, please refer to Table S4. Enrichment scores were calculated based on the classifier gene lists to estimated the expression enrichment of different CCA signatures.

### Global DNA methylation profiling datasets of ICCs

Three independent datasets of ICC global gene methylation profile were included, all of which were performed using Human Methylation450 BeadChip assays (Illumina, USA). The first combined cohort including 56 ICCs from Fudan University and Mayo Clinic (GSE32079) as we previously reported 40. The second cohort including 56 ICCs was from Singapore and Romania (GSE49656) 11. The third cohort was from ICGC cohort including 88 ICCs from multiple regions (GSE89803) 19. The details of the above included DNA methylation profiling datasets for analysis were summarized in Table S4. After the normalization step, probes that were differentially methylated between *IDH1*/*2* mutant and wild-type ICCs were obtained using the standard two-sample t-test with unequal variance and sample size. To adjust for multiple comparisons, we applied the Benjamini-Hochberg method to control the False Discovery Rate at 5%. We further filtered the list of significant CpGs by retaining those which exhibited at least 20% difference in methylation β-value between mutant and wild-type in our final comparisons.

### Statistical analysis

Analysis was performed with the Statistical Package for the Social Sciences (SPSS) v 15.0 software for Windows (SPSS, Chicago, IL, USA). All categorical results were presented as number and percentage and all continuous variables were presented as the median and range. The continuous and categorical variables were compared using the Fisher's exact test and Manne Whitney U test. OS and RFS were compared with the Kaplan-Meier method, and the significance was determined by the log-rank test. The Cox regression model was applied to evaluate the effect of each clinical variable and the mutational cluster on OS and RFS. Hazard ratios were calculated with adjustments for clinicopathological characteristics. Significance was determined at a two-sided p level of 0.05, except for p values in multiple comparisons, which was adjusted according to the method described by Benjamini-Hochberg.

## Results

### The interactive landscape of driver gene mutations in ICC

The discovery cohort included 505 WES/WGS and 300 STS cases (covering 90-497 genes). In total, 22 genes tested in at least 70% of samples and with mutation frequency > 2% were selected for further analysis (Figure S1 and Table S5). Among them, the most recurrently mutated genes were *TP53*,* KRAS*,* IDH1*/*2*,* ARID1A*,* BAP1, PBRM1, FGFR2 fusion, CDKN2A*, *SMAD4*, *PIK3CA, EPHA2* (mutation frequency > 5%). We used Fisher's exact test among all pairs of the 22 driver genes to search for statistically significant mutual exclusivity and co-occurrence. All pairs of these mutations were tested without imposing any prior biological knowledge (Figure 1B). To this end, statistically significant mutation-pairs (co-occurrences or mutual exclusivity) were found across 21 combinations of mutated genes, including 12 positive and 9 negative associations (Figure 1C-D).

### Pair-wise correlations of seven driver genes defined three mutational clusters

We then focused on the correlations of the most recurrently mutated genes. Among them, we found a strong tendency towards pair-wise co-occurrence of *TP53, KRAS* and *SMAD4* mutations. Strikingly, *IDH1/2, BAP1* mutations and *FGFR2-fusion* tend to be mutually exclusive with *TP53*, *KRAS* and *SMAD4* mutations, while *BAP1* mutations showed a strong tendency towards co-occurrence with *IDH1/2* mutations and *FGFR2*-fusions. By contrast, no such pair-wise associations were found in other recurrently mutated genes such as *ARID1A*, *PBRM1*, *CDKN2A*, *PIK3CA* and *EPHA2*. Given their high mutation frequencies and strong pair-wise associations, the seven genes (*TP53*, *KRAS*, *SMAD4*, *IDH1*/2, *FGFR2-fus* and *BAP1*) were selected to construct the basis of a mutational clustering system, Cluster1 with at least one mutation of *KRAS*/*TP53*/*SMAD4* mutations, irrespective of other mutations; Cluster2 with at least one mutation of *IDH*/*FGFR2-fus*/*BAP1* mutations and without *KRAS*/*TP53*/*SMAD4* mutations; Cluster3 with all the above genes being wild-type.

Then we clustered the 505 cases with WES/WGS data from the discovery cohort according to the proposed strategy. 167 (33.1%) and 117 (23.2%) cases harbored Cluster1 and Cluster2 mutations, respectively (Table S6). Cluster1 mutation and Cluster2 mutation show mutual exclusivity with strong statistical significance (OR = 0.0903, *P* < 0.0001). Other significantly mutated pathways or complexes, such as PI3K/AKT pathway (*PIK3CA*/*PTEN*) and SWI/SNF complex (*ARID1A, PBRM1, ARID2* and* SMARCA4*) mutations, tended to universally distribute among the clusters and were not appropriate for mutational clustering (Figure 1E). In Cluster1 cases, we observed a significantly higher tumor mutation burden (TMB) in *TP53*/*SMAD4* mutant cases but not in *KRAS* mutant cases (Figure S2). This prompted us to further divide Cluster1 into two sub-clusters, Cluster1A with *KRAS* mutation, irrespective of other mutations (intermediate TMB), and Cluster1B with *TP53*/*SMAD4* mutations and wild-type* KRAS* (high TMB). For Cluster2 mutant cases, depending on targetable *IDH1*/*2* mutations or *FGFR2-fus*, we further divide Cluster2 into three sub-clusters, Cluster2A with *IDH1*/*2* mutation, irrespective of other mutations, Cluster2B with *FGFR2-fus* and Cluster2C with *BAP1* mutation and wild-type* IDH1*/*2 or FGFR2.*

### The robustness of the mutational cluster was validated in different cohorts

To validate the robustness of the mutational clustering system, we first investigated the cases with STS data from the discovery cohort. Of 263 ICC cases with all the seven genes detected in this cohort, the mutation frequency of Cluster1 and Cluster2 genes was 36.9% (97/263) and 27.8% (73/263), respectively. Cluster1 mutation and Cluster2 mutation also tend to be mutually exclusive with statistical significance (OR = 0.1629, *P* < 0.0001, Figure S3A).

Then we validated this result in 3 additional cohorts. The internal validation cohort including 225 ICCs from our institute, with 41.3% (93/225) harboring Cluster1 mutations and 17.8% (40/225) harboring Cluster2 mutations (Table S7). The mutually exclusive pattern of Cluster1 and Cluster2 genes was statistically significant (OR = 0.0259, *P* < 0.0001, Figure S3B). The second cohort included NGS-based sequencing data of 212 ICC patients from Western countries (MSKCC cohort), and the third cohort included NGS-panel based sequencing data of 239 ICCs from the TIGER-LC and ICGC projects mostly from the Eastern countries (T-I cohort). Consistently, Cluster1 and Cluster2 mutations still showed strong mutually exclusivity with statistical significance (Figure S3C, OR = 0.1542, *P* < 0.0001 in the MSKCC cohort; Figure S3D, OR = 0.1361, *P* = 0.0004 in the T-I cohort). By contrast, *PTEN*/*PIK3CA*, SWI/SNF complex mutations were distributed universally among these two clusters. In these two NGS-based cohorts (MSKCC and T-I cohort), Cluster1B ICCs also exhibited the highest TMB level compared to Cluster2 and Cluster3 cases (Figure S4).

### Impact of clinical factors on the mutual exclusivity between Cluster1 and Cluster2 mutations

We further investigated the impact of major clinical factors on the mutual exclusivity between Cluster1 and Cluster2 mutations using the combined cohort. Cluster1 and Cluster2 mutations showed similar odds ratios (ORs) of mutual exclusivity among different gender (male/female), age (≥ 65 / < 65), and etiologies (Table S8). The OR of mutual exclusivity was higher in Western population than Eastern population (OR = 0.16 vs OR = 0.09, Table S8). Notably, tumor stage showed the highest impact on the mutual exclusivity between Cluster1 and Cluster2 mutations. The OR of Cluster1 and Cluster2 mutations in stage III/IV cases were more than 3 times higher than stage I/II cases (OR = 0.13 vs OR = 0.04, Table S8). For 16 Cluster1/2 co-mutant cases with clinical staging information available, 14 cases were stage III/IV (87.5%) (Table S9), which is significantly higher than cases harboring Cluster1 mutations (66.2%) or Cluster2 mutations (55%) only, indicating this rare Cluster1/2 co-mutation pattern may be the consequence during tumor evolution.

### Different mutational clusters exhibit distinct clinicopathological features

**Figure 2:**
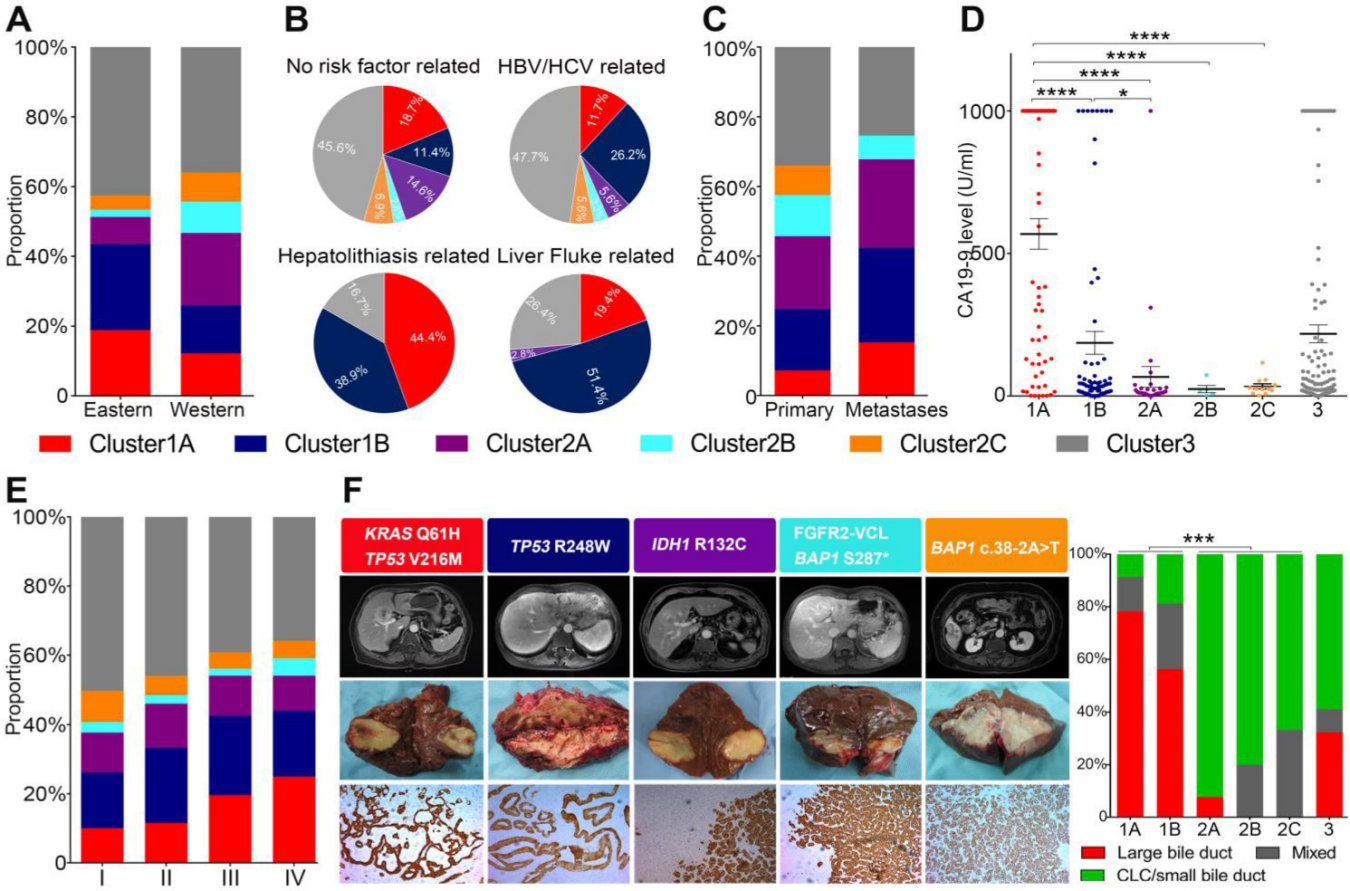
** Correlation of mutational clusters with clinicopathological factors, histological morphologies in ICC. (A)** Population distributuion,** (B)** etiological factors, **(C)** primary/metastases distribution, **(D)** CA19-9 levels and **(E)** AJCC staging for ICC between different mutational clusters. **(F)** Representative macroscopic and microscopic image of ICC cases with different mutational clusters.

Next, we compared the clinicopathological features of different clusters. Cluster1 mutations were more frequently found in Eastern populations, while Cluster2 mutations were more prevalent in Western populations (Figure 2A). Etiologically, Cluster2 was more often found in ICCs without clear risk factors and viral hepatitis related ICCs, but rarely found in cases with liver fluke infection or hepatolithiasis. while Cluster1A and Cluster1B were more frequently found in cases with liver diseases such as viral hepatitis, liver fluke infection and hepatolithiasis (Figure 2B). We also compared the prevalence of different mutational clusters between unpaired primary tumors and metastases in 212 ICC cases from the MSKCC cohort (145 primary tumors and 53 metastases). As shown in Figure 2C, Cluster1 mutations were significantly enriched in metastatic cases (*P* = 0.018). Cluster2 mutations showed similarly frequency between primary tumors and metastases, and Cluster3 mutations were more prevalent in primary than metastases. In addition, Cluster1A ICCs showed the highest levels of CA19-9, while Cluster2 ICCs exhibited much lower levels of CA19-9 than Cluster1A and Cluster1B (Figure 2D). Cluster1A ICCs were more enriched in stage III/IV cases compared with other clusters (Figure 2E).

According to histological morphology, ICC can be classified into small ductular and large bile duct subtypes. We thus explored the histological phenotypes of different mutational clusters in 94 ICC cases from the internal validation cohort. It was interesting to note that most of Cluster1 ICCs, especially Cluster1A cases showed large bile duct microscopical manifestations, while Cluster2 ICCs dominantly showed the phenotype of small duct (Figure 2F).

### Different mutational clusters of ICCs showed distinct clinical outcomes

**Figure 3:**
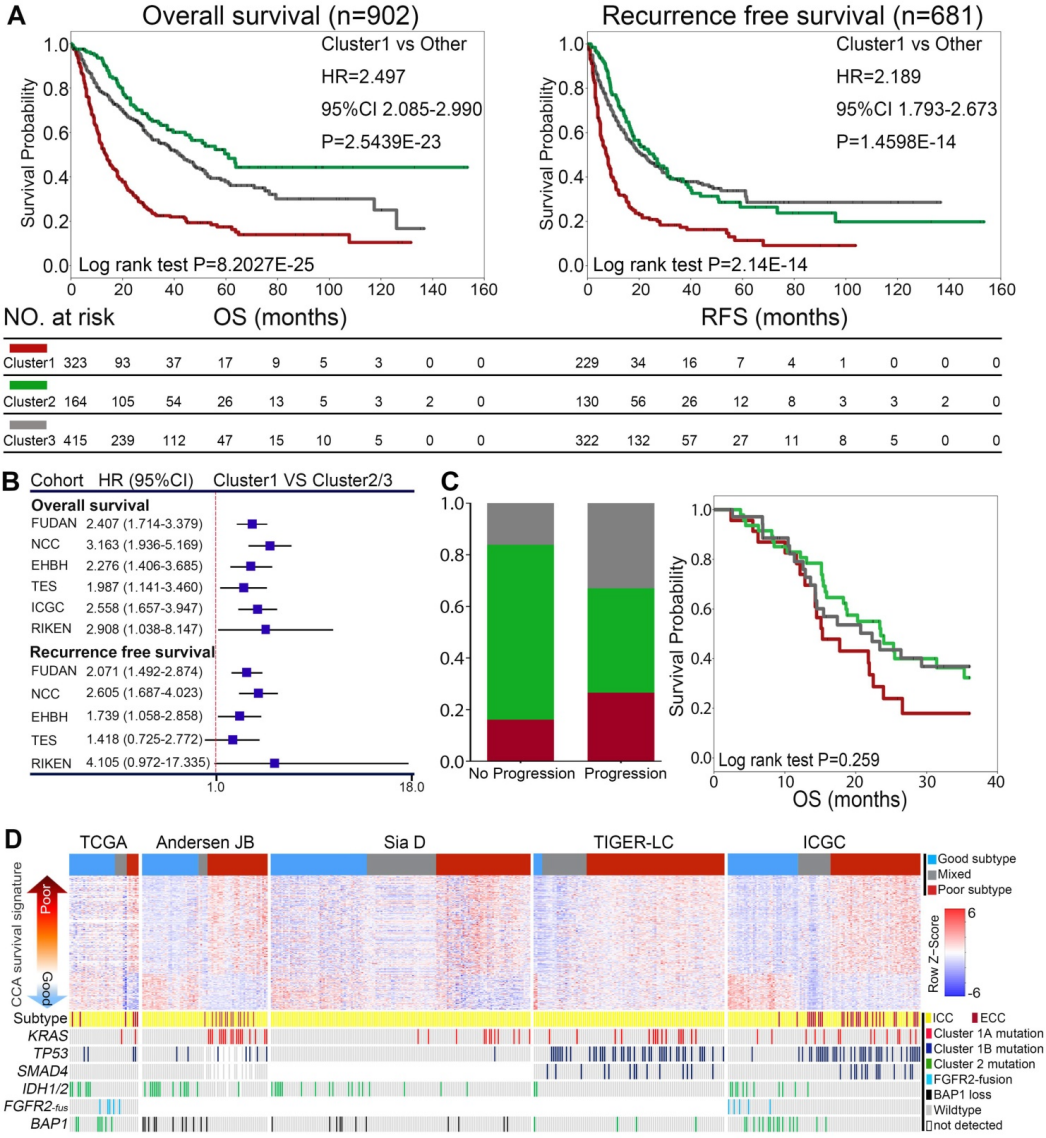
** Correlation of mutational clusters with prognosis in ICC. (A)** OS and RFS of surgical resected ICCs from the combined cohort showed different prognosis between mutational clusters. Log-rank test and Cox regression were used for the analysis. **(B)** Cluster1 showed significantly shorter OS and RFS than Cluster2/3 from the major subcohorts. **(C)** Cluster1 cases showed higher rate of progression on first line gemcitabine/platinum-based treatment (n = 104) and relatively worse OS compared with Cluster2/3 cases in metastatic/recurrent ICCs. **(D)** The distribution of mutational clusters in the “poor” and “good” signatures from independent gene expression profiling datasets.

We first evaluated the correlates of mutational clusters with clinical outcomes in surgical resected ICCs from the combined cohort. As shown in Figure 3A, Cluster1 mutations was significantly associated with worse overall survival (OS) and recurrence-free survival (RFS). In contrast, cases with Cluster2 mutations showed similar RFS but relative longer OS compared with Cluster3 ICCs. Among Cluster1 cases, Cluster1A and 1B ICC cases showed similarly poor prognosis (Figure S5A-B). The prognostic value of Cluster1 mutations were highly consistent in the major sub-cohorts (Figure 3B and Figure S6A-G). Consistently, the univariate and multivariate analysis showed that Cluster1 mutation status was an independent risk factor for OS and RFS in 345 surgical resected ICC cases from China (FUDAN and EHBH cohorts) (Figure S7A-B). Differently, Cluster1 mutation status did not show significant prognostic value in surgically resected ECCs (Figure S6H), suggesting that the observed prognostic value of the mutational clusters may be ICC specific.

Then we also investigated the prognostic values of different mutational clusters in advanced ICCs receiving palliative therapy. Among 104 metastatic/recurrent ICCs receiving first-line treatment (gemcitabine/platinum based) from the MSKCC cohort, Cluster1 cases showed higher rate of progression on first line treatment and relatively worse OS compared with Cluster2/3 cases, though the survival difference was not statistically significant (Figure 3C).

Together, the above results support an ICC specific prognostic value of Cluster1 mutations, while Cluster2 mutations show more favorable outcome.

### Deleterious partnering of Cluster1 mutations defined more invasive behaviors and a “poor prognosis” molecular signature

To investigate the biological basis of this prognostic distinction among the mutational clusters, we then analyzed the mutational status of 15 cholangiocarcinoma cell lines. All the tested cell lines harbored at least one *KRAS*, *TP53*, *SMAD4* or *IDH1* mutation and showed Cluster1 or Cluster2 mutational pattern, except for RBE cells which harbor a rare concurrent *KRAS* and *IDH1* mutations (Figure S8A). Cluster1 ICC cells showed significantly higher proliferative ability than Cluster2A ICC cells both *in vitro* and *in vivo* (Figure S8B-C).

A previously reported 238-gene classifier has classified cholangiocarcinoma into two prognostically different subclasses ('good prognosis' and 'poor prognosis') 41. Using supervised clustering based on the enrichment score of the 238 genes, we were able to divide cholangiocarcinoma cases into three (poor/good/mixed) groups in five independent gene expression profiling datasets (Figure 3D). Interestingly, we found that Cluster1 ICCs and ECC cases were mostly enriched in the “poor prognosis” subtype, while Cluster2 ICCs was mostly grouped in “good prognosis” subtype.

Together, Cluster1 ICC showed more aggressive biological behaviors and molecular features, correlated with the poorest clinical prognosis.

### Different mutational clusters demonstrated distinct histology and lineage related molecular features

**Figure 4:**
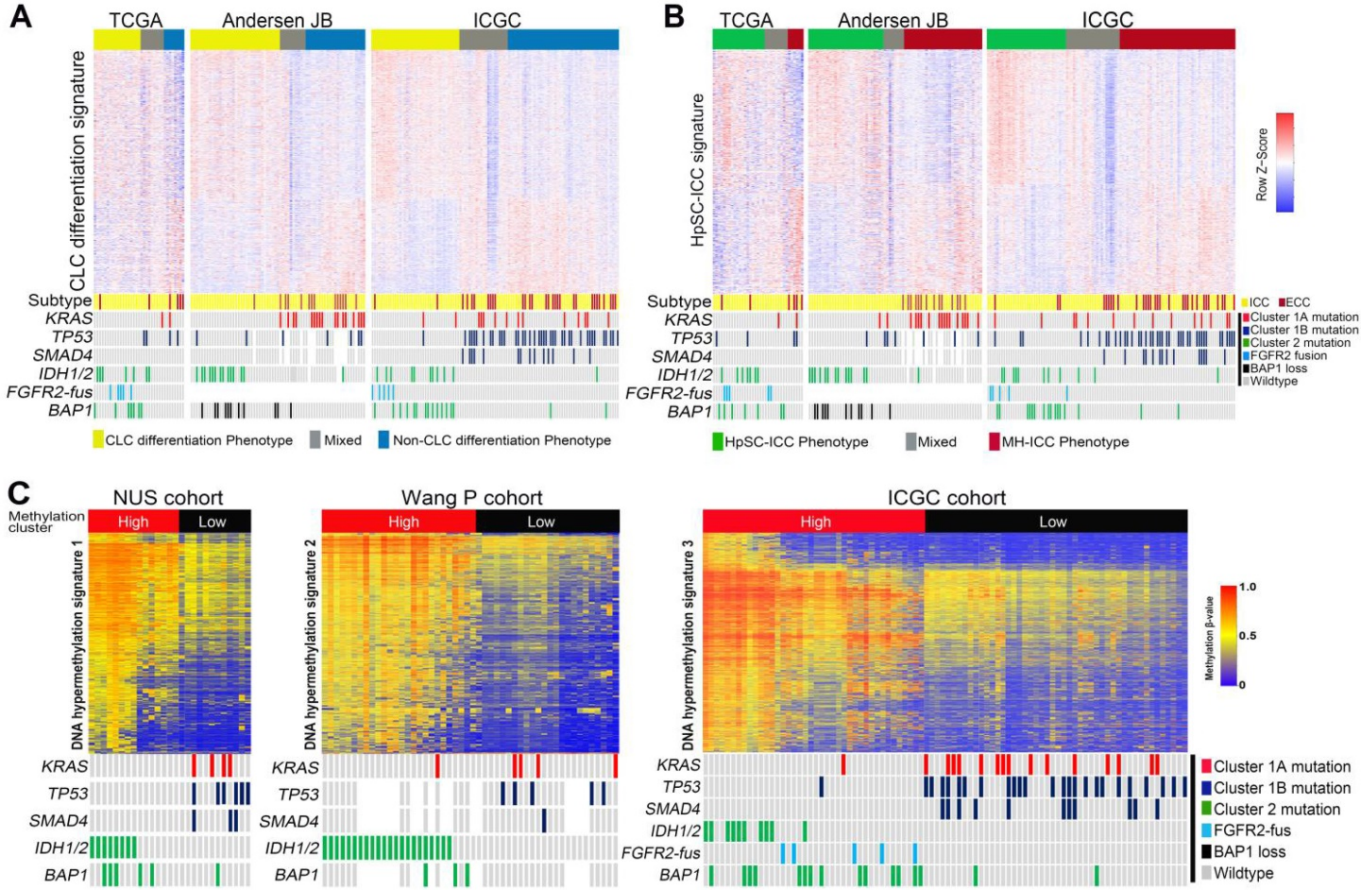
** Correlation of different mutation clusters with ICC gene expression signatures. (A)** The differential distribution of mutational clusters in CLC differentiation signatures in 3 different gene expression profiling datasets. **(B)** The differential distribution of mutational clusters in HpSC-ICC signatures in 3 different gene expression profiling datasets. **(C)** The differential distribution of mutational clusters in *IDH1*/*2* mutation-like methylation signatures in 3 different gene methylation profiling datasets.

Using a recently proposed cholangiolocellular-differentiation gene expression signature (CD signature) 42, we were able to divide CCA cases into CD phenotype, non-CD phenotype or mixed phenotype in three independent cohorts (Figure 4A). Interestingly, most Cluster2 ICCs were belong to the CD subtype, whereas Cluster1 ICCs and ECCs were mostly clustered into non-CD phenotype or mixed phenotype (Figure 4A). It was previously reported that a subset of ICC harbor stem cell features and is originated from liver progenitor cells 43. Using a hepatic stem cell-like (HpSC) gene expression signature described previously 44, we further divided CCAs into three (HpSC/non-HpSC/mixed) subtypes in the above three cohorts (Figure 4B). Most of Cluster2 ICC cases were enriched in the HpSC subtype, while Cluster1 ICCs and ECCs were mostly divided into non-HpSC or mixed phenotype. On the basis of DNA methylation, we found that Cluster2 ICCs exhibited significantly higher DNA methylation levels than Cluster1 ICCs, and that in Cluster2 ICCs, *FGFR2-fus* or *BAP1*-mutated ICCs (without *IDH1*/*2* mutation) showed similar DNA methylation pattern with *IDH-*mutated ICCs (Figure 4C).

In addition, we also identified distinct oncogenic gene expression signatures among different clusters. For instance, Cluster1 showed the highest expression level of a series of previously reported metastasis or progression related genes in ICC, such as *TMPRSS4, S100A4, S100P, PTEGS, MUC1, COX-2, IL-6* and *CEACAM6* (Figure S9) 45-50. In contrast, Cluster2 ICCs showed higher expression of a series of growth factors or receptors, such as* PDGFD* and *FGFR2*/*3*/*4*, all of which are known to play important roles in supporting ICC cell growth by mediating cancer-stromal interaction (Figure S10A) 51, 52. Notably, higher expression of FGFR2/3/4 and its downstream factors (GAB1, GRB2 and PTPN11) was observed in Cluster2 ICCs compared with Cluster1/3 cases (Figure S10B). This indicated that FGFR2/3/4 pathway activation exist across the Cluster2 ICCs, and FGFR2/3/4 targeted therapy may be potential for all Cluster2 ICCs, not only limited to *FGFR2-fus* (Cluster2B) ICCs.

### *IDH*/*FGFR2-fus*/*BAP1* and *TP53*/*SMAD4* co-mutation pattern further defined two prognostically different subsets of CCA-like HCC

**Figure 5:**
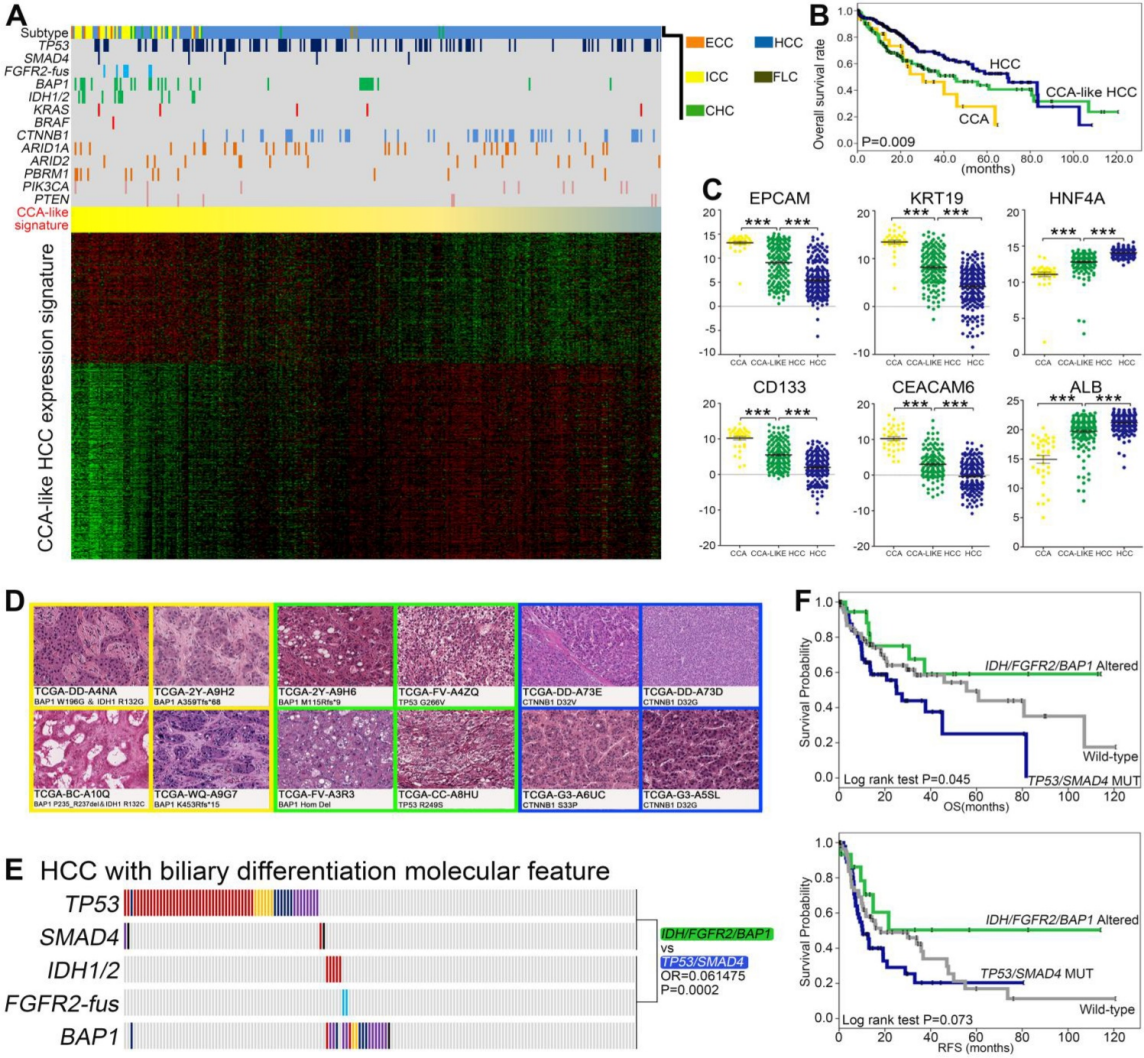
** The existence of Cluster1B (*TP53*/*SMAD4*) and Cluster2 (*IDH*/*FGFR2-fus*/*BAP1*) mutations in CCA-like HCC. (A)** Supervised clustering of CCA, CHC and HCC based on CCA-like HCC expression signature. **(B)** Kaplan-Meir plot analyses for OS between CCA, HCC and CCA-like HCC. **(C)** Analysis of HCC and ICC marker gene expression in CCA, CLHCC and HCC, respectively. Statistical significance was determined by Mann-Whitney test. **(D)** The representative histological characteristics from the group of CCA-like HCC samples, poor differentiated HCC and well differentiated HCC from TCGA cohort. **(E)** The mutually exclusive pattern of *TP53*/*SMAD4* mutations with *IDH*/*FGFR2-fus*/*BAP1* mutations in CCA-like HCC cohort. **(F)** Kaplan-Meir plot analyses for OS and RFS between Cluster1B mutations (*TP53*/*SMAD4*) and Cluster2 mutations (*IDH*/*FGFR2-fus*/*BAP1*) in CCA-like HCC.

As the three most common primary liver cancer (PLC) subtypes, HCC, ICC and CHC sometimes showed overlapping phenotypes, such as a previously proposed cholangiocarcinoma-like HCC (CLHCC) subtype 53. Using the CCA like traits (CC signature), we divided 407 TCGA-PLC (373 HCC, 36 CCA and 10 CHC) cohorts into a continuous liver cancer spectrum. Notably, 175 HCC/CHC cases showed CCA-like signature and clustered together with the 36 CCA cases, indicating that they intrinsically mimicked CCA molecular features (Figure 5A). Consistent with previous studies 53, the CCA, CLHCC and classical HCC showed different prognosis (Figure 5B), and the lineage specific markers (EPCAM, CD133, KRT19, CEACAM6, HNF4A and ALB) were also differentially expressed among these three subtypes (Figure 5C). Consistently, the microscopic phenotypes of these CLHCCs turned out to be “CCA-like” compared with the histological morphology of well differentiated HCCs (Figure 5D). Interestingly, we found significantly higher percentage of *TP53*/*SMAD4* mutations and *IDH*/*FGFR2-fus*/*BAP1* mutations, while lower mutation rate of *CTNNB1* in CLHCC subtype than classical HCC subtype (Figure 5A). Notably in CLHCC, four of the five *IDH1*/*2* mutant cases and both the two *FGFR2-fus* cases harbored concurrent *BAP1* mutations, and two of the four *SMAD4* mutations were co-mutated with *TP53* mutations. Consistent with in ICC, these two clusters also tend to be mutually exclusive (OR = 0.061475, *P* = 0.0002, Figure 5E), and Cluster1B CLHCCs also showed significantly shorter OS and RFS compared with Cluster2 CLHCCs (Figure 5F).

### The ICC cells bearing different clustering mutations demonstrated different sensitivities to molecular targeted treatments

**Figure 6:**
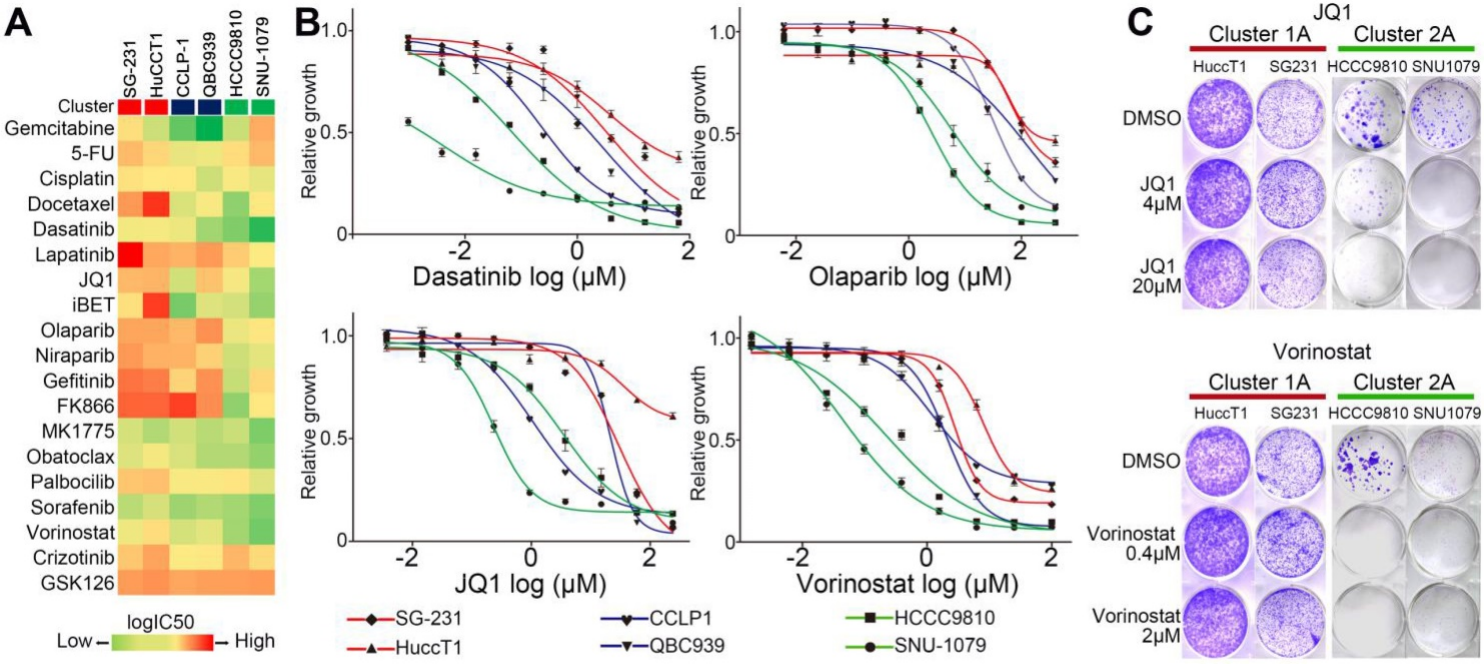
** Different responses to small molecular drugs among ICC cell lines with different mutation clusters. (A)** Heat map illustrating the median-centered Log(IC50) of 6 ICC cell lines screened across 19 clinically relevant compounds. **(B)** Cluster1 and Cluster2 cell lines were treated with dasatinib, olaparib, JQ1 and vorinostat; log(IC50) was determined at day 3 post-treatment. **(C)** Crystal violet staining of viable cells treated with JQ1 and vorinostat.

Considering the molecular distinctions between Cluster1 and Cluster2 ICCs, it is potentially interesting to compare their responses to molecular targeted treatments. We screened six ICC cell lines (including two Cluster1A, two Cluster1B and two Cluster2A mutant cell lines) against a panel of compounds which are being tested in clinical trials or FDA-approved, including ones targeting tyrosine kinase receptors, epigenetic regulation, metabolism, DNA damage, and cell cycle (Figure 6A and Table S10). Cell viability assays demonstrated that no compound could selectively decrease the viability of Cluster1A and 1B mutant ICC cells (Figure 6A). Strikingly, Cluster2A mutant ICC cells exhibited increased sensitivity to Dasatinib, Vorinostat, JQ1, iBET, Olaparib, and Niraparib than Cluster1A mutant cells (Figure 6A-B). Considering that Dasatinib and PARP1 inhibitors have been reported in *IDH1*/*2* mutant ICC or other cancers 54-56, we tested the effect of JQ1 and Vorinostat on clone formation ability in four ICC cell lines. As shown in Figure 6C, JQ1 and Vorinostat significantly inhibited Cluster2A (*IDH1* mutant) ICC cells, but not Cluster1A (*KRAS*/*TP53* mutant) ICC cells.

The results from this preliminary study suggest that the molecular subtypes of ICCs may be related to distinct sensitivities to certain targeted therapies. A larger scale drug screening based on different mutational clusters of ICC deserves further investigation.

### Gene expression profiles integration converged on S100P and KRT17 as specific biomarkers for mutation-free ICCs

**Figure 7:**
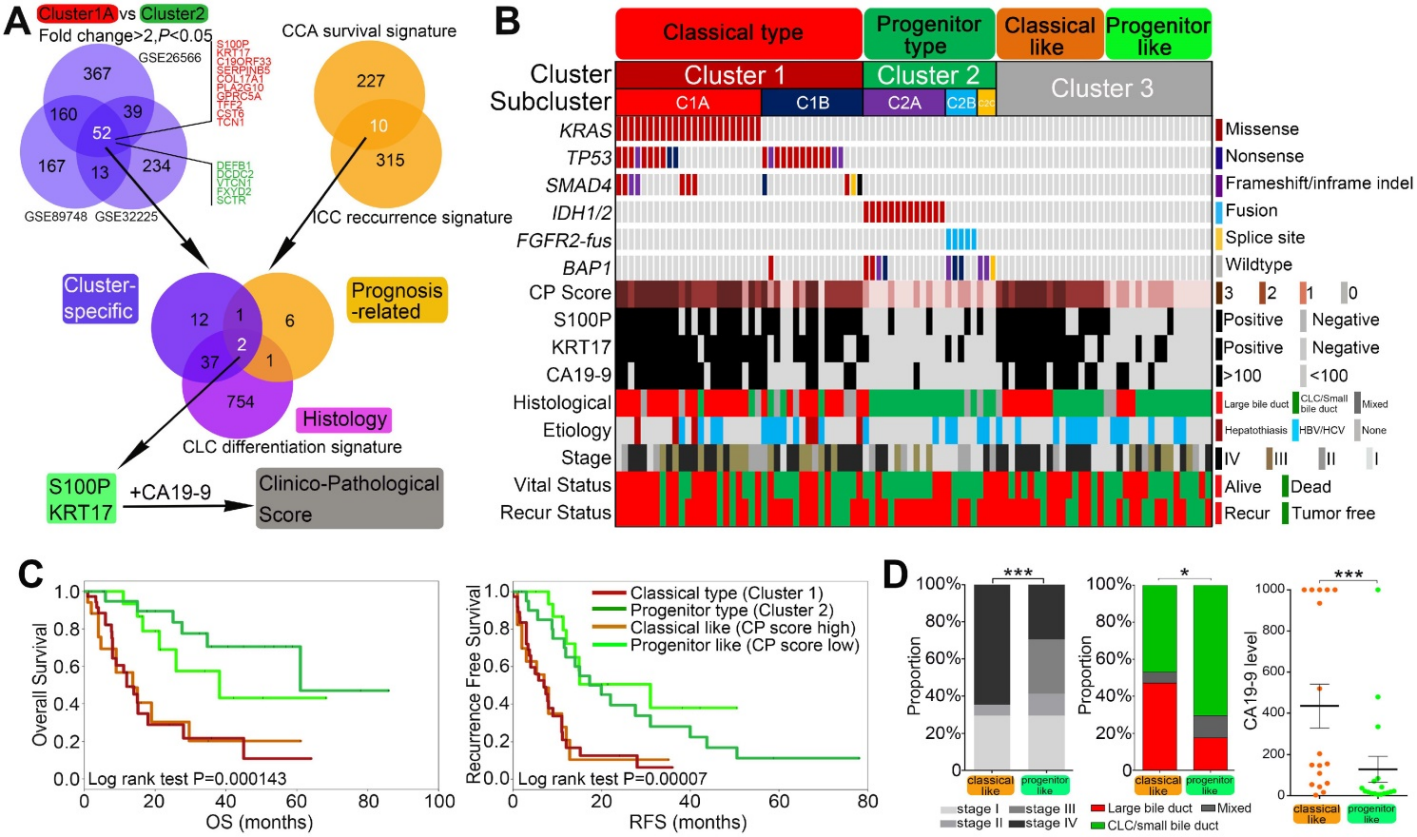
** Integrated clinico-pathological score (CP Score) further stratified mutational clusters into biological relevant subtypes (modified clusters). (A)** Venn diagram 1 showing overlaps of differential expressed genes between Cluster1A and Cluster2 mutations from 3 independent cohorts. Venn diagram 2 showing overlap of 10 prognosis related genes from 2 well established gene signatures. Venn diagram 3 showing overlap of cluster specific, prognosis related and histological relevant genes. Then a clinicopathological score (CP score) comprising S100P, KRT17 and CA19-9 was constructed. **(B)** A modified clustering system stratified by clinicopathological score could better reflect the biological relevant of the mutational cluster. **(C)** Kaplan-Meir plot analyses for OS and RFS among different modified clusters. **(D)** AJCC 8^th^ staging, microscopic morphology and CA19-9 levels for ICC between classical-like and progenitor-like subclusters from Cluster3 patients.

As mentioned above, Cluster1A and Cluster2 ICCs show distinct clinical features, cell of origins, and therapeutic responses. By comparing the gene expression profiles of Cluster1A and Cluster2 from three independent ICC gene expression profiling datasets (Figure 7A), we obtained a 52-gene set that was universally differentially expressed between Cluster1A and Cluster2 ICCs (with average fold change > 2 and *P* < 0.05 in all three datasets) (Table S11). Among the genes (average fold change > 4) that mostly discriminated Cluster1A and Cluster2 (Figure 7A), the top two genes S100P and KRT17 were both prognostic markers in Andersen's CCA survival signature and Sia D's ICC recurrence signature, as well as the CD signature (Figure 7A). We found that S100P and KRT17 were positively correlated with each other in all nine ICC gene expression datasets (Figure S11). Moreover, ICCs with high expression of both S100P and KRT17 was associated with worse prognosis than those with low expression of both S100P and KRT17 (Figure S12). We then assessed the protein levels of S100P and KRT17 in 94 patients from the internal validation cohort, including 39 cases of Cluster1, 21 cases of Cluster2, and 34 cases of Cluster3. In according with mRNA expression result, IHC staining revealed that Cluster1A ICC showed significantly higher proportion of S100P- and KRT17-positive staining than the other clusters (Figure S13), indicating that S100P and KRT17 may become the cluster-specific, prognosis and histology-relevant biomarkers in ICC.

In addition, previous studies 57 and our present study indicated that high CA19-9 level was also cluster-specific, prognostic and histology relevant. To construct a clinicopathological score (CP score) system that permit simple and cost-effective classification of ICC in clinical practice, we finally evaluated the clinical application potential of S100P (positive/negative) and KRT17 (positive/negative) in combination with CA19-9 (≥ 100 U/ml / < 100U/ml). Low CP score (0 and 1) could well defined Cluster2 ICCs, while Cluster1 ICCs were mostly enriched in CP score high (2 and 3) (Figure 7B). ROC curve analysis suggested that the sensitivity and specificity of CP score for cluster prediction was much higher than that of CA19-9 (sensitivity 84.6% vs 66.7%; specificity 100% vs 95.2%) (Figure S14). This indicated that CP score had a better performance compared with CA19-9 alone for distinguishing Cluster1 ICCs from Cluster2 ICCs. Furthermore, this score system could also stratify Cluster3 cases into two subsets with distinct clinical and biological relevance. Cluster3 cases with high CP scores showed dismal outcomes, high TNM stages, large bile duct phenotype and high CA19-9 level compared with cases with low CP scores (Figure 7C-D).

**Figure 8:**
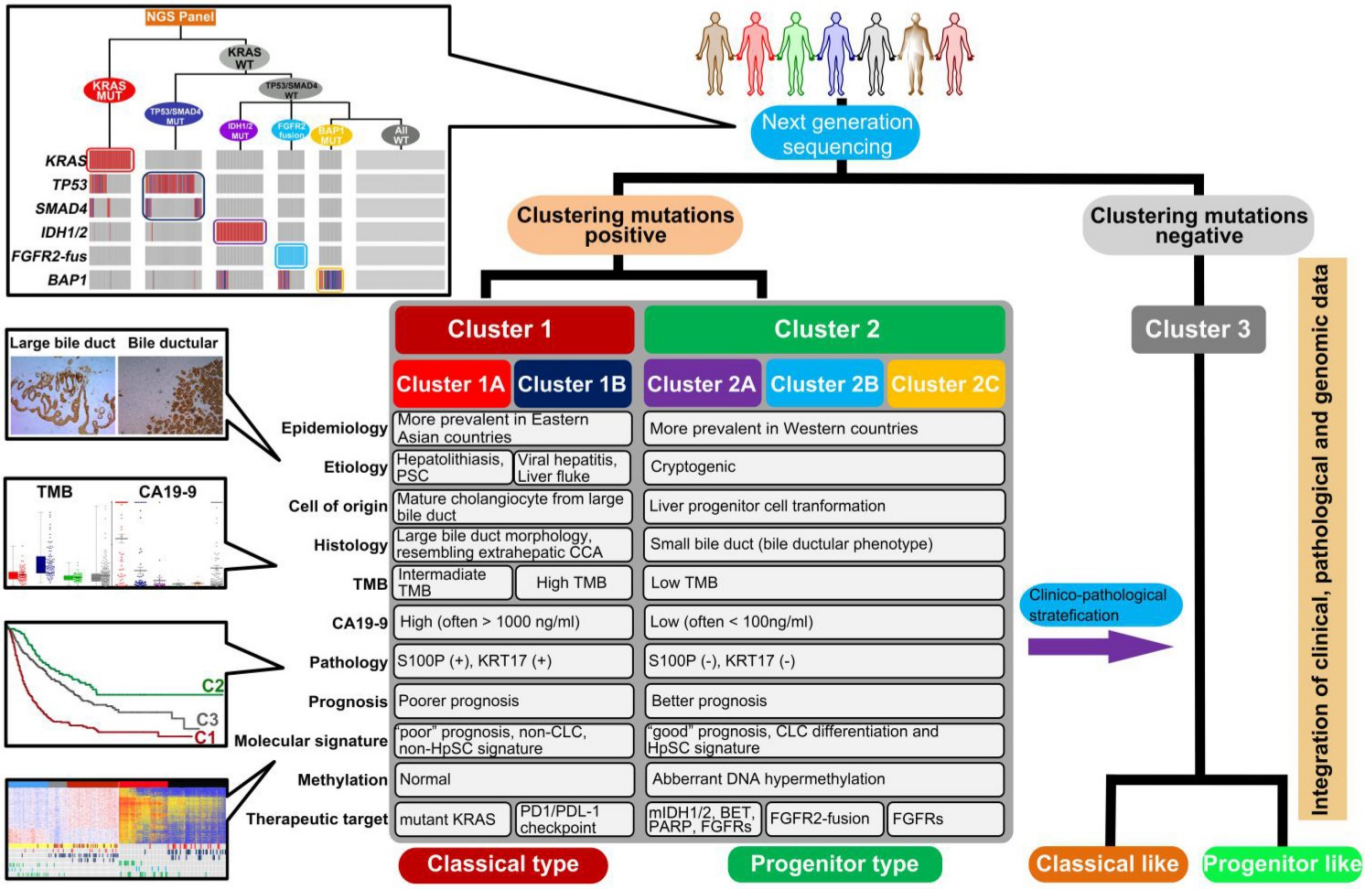
Summary of clinical management procedure and characteristics of the subtypes of ICC

Taken together, these results suggest that S100P and KRT17 combined with CA19-9 may act as lineage dictating markers helping to differentiate mutational clusters in ICC. Combining clustering mutations and subtype-specific biomarkers, we finally propose a clinically applicable clustering strategy, which can be instructive to ICC prognostic stratification, molecular classification, and therapeutic optimization (Figure 8).

## Discussion

In this study, by investigating the pair-wise co-occurrence or mutual exclusivity of driver mutations, we described the cooperative landscape of the driver mutations and uncovered the mutational basis of ICC diversity. Notably, most ICCs mutually exclusively harbor Cluster1 or Cluster2 mutations, which will be conducive to establish a clinically applicable strategy to rapidly and precisely identify different prognostic and biological subtypes of ICCs.

With the purpose of better understanding the molecular heterogeneity of ICC, several gene expression profiling-based subtyping models have been proposed to classify ICC into putative discrete subtypes, such as the “good and poor” signature 41, “proliferation and inflammation” signature 58, “HpSC” signature 43 or others 16, 20, 42, 59. More recently, integrative multi-omics strategies were also used to establish subtyping system for cholangiocarcinoma 18, 19, 60. All these studies contributed to a more comprehensive understanding of the molecular heterogeneity of ICC from different perspectives. However, these gene expression or DNA methylation based subtyping signatures included hundreds of genes, which greatly limited their translation into clinical practice. In addition to the intrinsic molecular traits of malignant cells, tumor transcriptional subclasses differ according to the composition of the tumor microenvironment, especially the existence of prominent stromal cells 61, 62. These disadvantages may restrict their applications in the clinical practice, and a clinically applicable, molecular relevant clustering strategy is still lacking in ICC.

Malignant transformation is a highly cooperative process, during which functionally synergistic gene mutations are frequently co-selected and observed together in the same tumor subtypes, and their cooperative effects are associated with disease progression and clinical outcome. Supporting this notion, the co-association or mutual exclusivity of *IDH1*/*2*, *TP53* and *CIC*/*FUBP1* mutations construct the basis of a new molecular pathological classification in gliomas 63, 64. Similarly, according to the mutational status of TP53, MDM2, RAS, and FGFR3, non-hypermutated upper urinary tract urothelial carcinoma (UTUC) was classified into four subtypes showing unique co-alteration/mutually exclusive patterns, and different mutational subtypes have discrete profiles of gene expression, tumor location/histology, and clinical outcome 65. Using the mutation relevant clustering strategy, ICC was previously divided into three subgroups based on the genomic perturbation of *KRAS*, *TP53* and *IDH1* 66. Based on comprehensive analysis, we uncovered the mutational basis of ICC diversity and divided ICCs into 3 mutational clusters by the mutation status of seven driver genes. Traditionally, *TP53*, *KRAS* and *SMAD4* mutations are classical gene mutations that are ubiquitously distributed among pancreatobiliary malignancies. Previous preclinical studies have also revealed that these gene mutations corroborated to promote the initiation and metastasis in these cancer types 67-69. Supporting this, our result revealed that these mutations tended to be co-occurring with each other in ICC. Compared to the trans-cancer type distributing pattern of Cluster1 mutations, *IDH1*/*2*, *BAP1* mutations and *FGFR2* fusions in Cluster2 were predominantly identified in ICC but rare in ECC or pancreatic ductal adenocarcinoma (PDAC), and our result revealed that all of the three genomic alterations tended to be mutually exclusive with Cluster1 mutations with extremely low overlapping frequencies. Notably, our mutational clusters were well relevant to most of the previously proposed gene expression signatures, the prognostic signature 41, the the CD signature 42 and the HpSC signature 43 as we demonstrated above.

Although the mutually exclusive of Cluster1 and Cluster2 mutations tend to be extremely significant, there are a still mall proportion of cases harboring both Cluster1 and Cluster2 mutations (31/1481, 2.1%). For 16 Cluster1/2 co-mutant cases with clinical staging information available, we found that most cases were stage III/IV (14/16, 87.5%). The proportion of stage III/IV was significantly higher in these Cluetr1/2 co-mutated cases than those with Cluster1 or Cluster2 mutations only. Consistently, in one study not included in the current research showing the highest Cluster1/2 co-mutation rate (7/55, 12.7%), although the individual clinical staging information was not available, 51/55 (92.7%) cases were stage III/IV ICCs 70. These results indicated that this rare Cluster1/2 co-mutation pattern may be the consequence during tumor evolution and more enriched in advanced stage ICCs.

Clinically, the prognostication of ICC has long been relegated to clinical staging/scoring systems or nomograms 71-74, with an absence of stable molecular markers. A major clinically relevant finding of this study is that clustering of ICC based on Cluster1 mutation status may be predictive of prognosis (OS and RFS), providing the potential of adding a reliable “biological” parameter to improve the accuracy of currently used clinical prognostic systems. Previous studies have already revealed that activating mutations in the *KRAS* oncogene and inactivating mutations/deletions in *SMAD4* and *TP53* tumor suppressor genes are significantly correlated with poor clinical outcomes in ICC 13, 16. Using independent datasets, we show here that the combination of Cluster1 mutation status is also a strong prognostic marker for poor prognosis (shorter OS and RFS after surgical resection), even adjusting for TNM stage and CA19-9 level. We also found that Cluster1 mutant ICCs were more prevalent in metastatic lesions compared with Cluster2/3 mutations. At molecular levels, we found that Cluster1 mutant ICC and ECC cases were mostly enriched in the 'poor prognosis' group, while Cluster2 mutations were mostly clustered into the 'good prognosis' group. Taken together, we propose that the Cluster1 mutation status is a strong predictor for poor prognosis, including survival, recurrence and metastasis. High-risk clinicopathological factors (e.g. CA19-9 level, TNM stage and metastasis), highly proliferative and invasive biological behavior, and the enrichment of 'poor prognosis molecular signature' can well explain the poor prognosis of Cluster1 mutations from clinical, biological and molecular perspectives. We believe that adding biological (non-anatomic) factors such as Cluster1 mutations may provide an important optimization for currently used ICC prognosis prediction models. Although the mutational clusters also existed in ECC, here we found that the prognostic value of Cluster1 mutations was not applicable, indicating ECC and ICC were not only distinct in location, but also in intrinsically biological behaviors.

The first-line therapeutic choice for advanced-stage ICC is extremely lacking except gemcitabine/cisplatin combined chemotherapy 75. In the second-line setting, although several targeting therapeutic drugs (ivosidenib and pemigatinib) have been approved for clinical treatment, the low response rate (objective response rate = 2% for ivosidenib from the ClarIDHy study) 76 or high rate of primary/acquired resistance still restrain their therapeutic efficacy 77. In this study, we employed a panel of small molecular drug screening to search for potential cluster-specific dependencies of ICC cell lines, and several potential therapeutic targets were identified in Cluster2A (IDH1 mutant) ICC cell lines. Among them, we found increased sensitivity of Cluster2A mutant ICC cells to Dasatinib and PARP inhibitors as previously reported in ICC or other cancer types 54, 56. Moreover, we also found increased sensitivity of Cluster2A mutant cell lines to BET inhibitors and HDACs inhibitors. Considering the low response rate of IDH1 mutant CCA to ivosidenib, the therapeutic vulnerabilities of IDH1 mutant CCA to these drugs deserve further investigation.

In the field of oncoimmunology, extensive efforts have been made recently to identify robust predictive markers of the therapeutic response to immune checkpoint inhibitors. Several markers had been proposed, including tumor PD-L1 expression, high TMB, POLE mutation and DNA mismatch repair deficiency. In the current study, higher TMB was observed in Cluster1 gene mutant tumor tissues compared with Cluster2 ICCs. A positive correlation between PDL-1 level and *TP53* mutation in CCA has also been reported in a recent study 78. Interestingly, the positive relation of PD-L1 level and *TP53*/*KRAS* mutation has also been reported in lung cancer 79, and recent studies has found potential predictive value of *TP53* and *KRAS* mutation status for response to PD-1 blockade immunotherapy in lung adenocarcinoma 80, 81 Mechanistically, recent studies have revealed that PD-L1 mRNA is regulated by oncogenic RAS signaling and *TP53* mutation 82, 83. The above evidence indicated that the mutational clusters of ICCs may have potential roles in predicting the therapeutic response to anti-PD-1 therapies, which deserves further investigation.

Another clinically relevant finding of this study is the identification of two biomarkers (S100P and KRT17) derived from integrated gene expression profiling analysis. Furthermore, we propose that S100P and KRT17 in combination with CA19-9 may serve as cluster-specific, prognosis and histology relevant biomarkers to optimize the mutational classification and prognostication. These three markers are chosen for the following reasons, Firstly, they are the most prominent pathological or clinical markers to discriminate Cluster1A and Cluster2 ICCs (with > 8-fold change). Secondly, they are all independent prognostic factors in ICCs as reported by us and others 41, 46, 57, 58. Thirdly, they are all parameters that are differentially expressed in CLC and large bile duct type ICC 42. Furthermore, S100P and KRT17 have been reported to play critical roles in malignant progression and metastasis in ICC and other malignancies 46, 84. In our current study, the CP-score can further divide the “mutation-free” Cluster3 ICC into CP-high and CP-low subtypes, which display distinct prognosis and histological features. The S100P(+)/KRT17(+)/CA19-9(high) group showed Cluster1A-like prognostic and histological features, such as large bile duct differentiation phenotype. In contrast, the S100P(-)/KRT17(-)/CA19-9(low) group showed more similarities with Cluster2 ICC, with CLC differentiation phenotype and better prognosis.

In conclusion, our study provides evidence that routine gene test for the seven mutated genes, together with the evaluation of S100P/KRT17 expression and CA19-9 level, represent robust biomarkers to identify clinically and biologically distinct subtypes of ICC. Moreover, we show that different mutational clusters undergo distinct mechanisms of tumorigenesis and thereby differ in drug responsiveness, which would open the door for a more precise therapeutic strategy for this refractory malignancy. In the future, selectively prognostic and therapeutic stratification may be suggested in clinical practice for ICC patients with different mutational clusters.

## Supplementary Material

Supplementary materials and methods, figures, tables 2-11.

Supplementary table 1.

## Abbreviation

CCA: cholangiocarcinoma
ICC: intrahepatic cholangiocarcinoma
BTC: biliary tract cancer
ECC: extrahepatic cholangiocarcinoma
PLC: primary liver cancer
HCC: hepatocellular carcinoma
CHC: combined hepatocellular-cholangiocarcinoma
PDAC: pancreatic ductal adenocarcinoma
TP53: tumor protein p53
KRAS: Kirsten rat sarcoma oncogene homolog
SMAD4: SMAD family member 4
IDH1: isocitrate dehydrogenase 1
IDH2: isocitrate dehydrogenase 2
BAP1: BRCA1 associated protein 1
FGFR2: fibroblast growth factor receptor 2
PBRM1: polybromo 1
CA19-9: Carbohydrate Antigen 19-9
S100P: S100 calcium binding protein P
KRT17: keratin 17
T2DM: type 2 diabetes mellitus
PSC: primary sclerosing cholangitis
STS: selected targeting sequencing
WES: whole exome sequencing
WGS: whole genome sequencing
TMB: tumor mutation burden
NGS: next generation sequencing
AJCC: American Joint Committee on Cancer
TNM: tumor-node-metastasis
GENIE: Genomics, Evidence, Neoplasia, Information, Exchange
AACR: American Association for Cancer Research
ICGC: International Cancer Genome Consortium
TIGER-LC: Thailand Initiative in Genomics and Expression Research for Liver Cancer
TCGA: The Cancer Genome Atlas
MSKCC: memorial sloan-kettering cancer center
FFPE: formalin-fixed, paraffin-embedded
IHC: immunohistochemistry
