# Supplementary material for: Driver mutations of intrahepatic cholangiocarcinoma shape clinically relevant genomic clusters with distinct molecular features and therapeutic vulnerabilities
Source: Theranostics. 2022 Jan 1;12(1):260–76. doi: 10.7150/thno.63417 (PMC8690927; doi:10.7150/thno.63417)
Supplement: Supplementary file 1 — Supplementary materials and methods, figures, tables 2-11. [file thnov12p0260s1.pdf]

Driver mutations of intrahepatic cholangiocarcinoma shape clinically relevant genomic clusters with distinct molecular features and therapeutic vulnerabilities

Xiang-Yu Wang, Wen-Wei Zhu, Zheng Wang, Jian-Bo Huang, Sheng-Hao Wang, Fu-Mao Bai, Tian-En Li, Ying Zhu, Jing Zhao, Xin Yang, Lu Lu, Ju-Bo Zhang, Hu-Liang Jia, Qiong-Zhu Dong, Jin-Hong Chen, Jesper B. Andersen, Dan Ye and Lun-Xiu Qin

Supplementary materials and methods

Dataset analyses

For WES/WGS cases, the raw sequencing data of 321 ICC samples and matched normal samples from previous studies (accession ID: SRP025150; SRP045202; SRP050003; EGAS00001000389; EGA00001000950; EGAS00001001653) were downloaded (Table S1). We processed raw sequences (FASTQ files) to filter low quality reads and trimmed adapter sequences. Resulting sequences were mapped to the human genome assembly (hg19) using Burrows-Wheeler Aligner (BWA) software by default parameters [1]. Mapped BAM files were filtered to remove PCR duplicates and excluded multi-mapping reads using samtools [2]. Only unique mapping reads were used for downstream analysis. Somatic substitutions were detected by VarScan[3] and high confident somatic SNVs were called if the following criteria were met (1) both the tumor and normal samples should be covered sufficiently ($\geqslant$ 10 $\times$) at the genomic position; (2) the variants should be supported by at least 10% of the total reads in the tumor while less than 2% in the normal; (3) the variants should be supported by at least three reads in the tumor; (4) distance between adjacent somatic SNV distance should be over 10bp. High confident somatic insertions and deletions (indels) were called using the following steps: (1) candidate somatic indels were predicted with GATK SomaticIndel Detector with default parameters; (2) for each predicted somatic indel, local realignment was performed with combined normal and tumor bam files; (3) high confident somatic indels were defined after filtering germline events. All high confident somatic mutations were filtered out by the dbSNP135 and the remaining mutations were annotated with ANNOVAR and subjected to subsequent analyses. For the remaining 184 WES/WGS cases with raw sequencing data unavailable, processed data was fully retrieved from the supplementary materials of the original publications or the depository databases (Table S1).

For STS cases, processed data was fully retrieved from the supplementary materials of the original publications, and samples without detailed information on specific gene mutations were excluded. All the above mutational data was carefully censored by two investigators (Xiang-Yu Wang and Zheng Wang) independently.

Meta-analysis of mutational heterogeneity across multiple cancers have shown that some members of certain gene families, such as MUCIN, NEUROBLASTOMA BREAKPOINT FAMILY

and TTN are significantly mutated across multiple cancers [4]. These genes are unlikely to have association in the context of ICC pathogenesis and thus excluded from downstream analysis.

DNA extraction and sequencing

Genomic DNA was extracted using the QIAamp DNA mini kit for snap-frozen samples or the Qiagen DNA FFPE tissue kit (Qiagen Inc, Valencia, CA, USA) for paraffin-embedded samples according to the manufacturer's instructions. Non-cancerous liver tissues were used as the normal tissue. Sanger sequencing of all exons of *TP53*, *SMAD4* and *BAP1*, exon 2, 3 and 4 of *KRAS* gene, exon 3 of *IDH1* and exon 4 of *IDH2*, were carried out in 123 snap-frozen tissues as previously described [5]. NGS platform based genomic profiling was performed in a Clinical Laboratory Improvement Amendments-certified and College of American Pathologists-accredited laboratory (3DMedcines Inc., China) for the sequencing of the rest 102 FFPE tissues. DNA extracts (30-200 ng) were sheared into 250-bp fragments using an S220 focused-ultrasonicator (Covaris). Libraries were prepared using the KAPA Hyper Prep Kit (KAPA Biosystems) following the manufacturer's protocol. For targeted capture, indexed libraries were subjected to probe-based hybridization with two customized NGS panels targeting 381 cancer-related genes or 36 ICC-related genes (Table S3), where the probe baits were individually synthesized, 5' biotinylated, 120-bp DNA oligonucleotides (IDT), and repetitive elements were filtered out from intronic baits according to the annotation provided by UCSC Genome RepeatMasker. The captured libraries were loaded onto a NovaSeq 6000 platform (Illumina) for 100-bp paired-end sequencing with a mean sequencing depth of 500×. Raw data of tissue samples were mapped to the reference human genome hg19 using the BWA software. Variant calling was performed only in the targeted regions. Somatic single nucleotide variants (SNVs) were detected using MuTect (v1.1.7) (https://github.com/broadinstitute/mutect), and somatic insertions and deletions (indels) were detected using Pindel (v0.2.5a8) (http://gmt.genome.wustl.edu/packages/pindel) with default parameters. Copy number variations (CNVs) were called by an in-house developed script with a cutoff of 6 copies. Gene rearrangements were identified by analyzing the clipped reads that could be extracted by the tag information of bam files mapped by BWA software. Single-nucleotide polymorphisms (SNPs) and indels were annotated by ANNOVAR against the following databases: dbSNP (v138), 1000 Genomes and ESP6500 (population frequency > 0.015). Only missense, stopgain, frameshift and nonframeshift indel

mutations were retained. This study was approved by the Ethics Committee of Huashan Hospital, Fudan University; and all subjects agreed with informed consent to participate in the study.

Cell lines and cultures

Eight human cholangicarcinoma cell lines were used in our study, including seven human ICC cell lines: HCCC9810 and RBE (Chinese Academy of Sciences Cell Line Bank, China), SNU-1079 (Korean Cell Line Bank, Korea), HuccT1 and Huh28 (Riken BioResource Center, Japan), CC-LP-1 and SG-231 were kind gifts from Dr. Yue Xiong. A perihilar cholangiocarcinoma cell lines QBC-939 was a kind gift from Dr. Shuguang Wang (Chongqing, China). All the cell lines were maintained in 1640 with 10% fetal bovine serum (FBS) and 1% penicillin/streptomycin mixture. All cell lines were authenticated by morphology and biologic behavior, and were routinely tested negative for mycoplasma contamination before experiments.

Cell proliferation

Cell proliferation was determined by direct cell number counting. In brief, cell lines were seeded in triplicate in 6-well plates at initial density of 3*10^4/well. Culture medium was refreshed every day. The cell number was counted at the indicated time points using BioTech Automated Cell Counter System under the manufacture's instruction (Countstar).

Tumor xenograft mouse models

Animal experiments were approved by the Committee on the use of live animals for teaching and research of Fudan University. Different ICC cell lines (10^6 cells) were suspended in 100 μl serum-free RPMI/Matrigel (BD Biosciences; 1:1 volume) and inoculated subcutaneously into the flank of 6- to 8-week-old male NOD/SCID mice under anesthesia. The mice were monitored weekly for palpable tumors for 8-10 weeks.

***In vitro* drug screening**

Cells were plated in 96 well plates (4000 cells/well) in culture medium. The following day, increasing doses of either Gemcitabine (S1714, Selleck Chemicals, USA), 5-FU (S1209, Selleck Chemicals, USA), Cisplatin (S1166, Selleck Chemicals, USA), Oxaliplatin (S1224, Selleck

Chemicals, USA), Docetaxel (S1148, Selleck Chemicals, USA), Sorafenib (S7397, Selleck Chemicals, USA), Dasatinib (S1021, Selleck Chemicals, USA), Gefitinib (S1025, Selleck Chemicals, USA), Lapatinib (S2111, Selleck Chemicals ,USA), JQ1 (S7110, Slleck Chemicals, USA), GSK126 (S7061, Selleck Chemicals, USA), Olaparib (S1060, Selleck Chemicals ,USA) or Niraparib (S2741, Selleck Chemicals, USA) was added and the cells were allowed to grow until DMSO-treated wells reached confluence (three days). To quantify viable cells, MTT (M-6494, Thermo Fisher Scientific, USA) was added to the culture media at a final concentration of 1 mg/ml and incubated for 3 h at 37°C. Formazan crystals were solubilized with 100 µL/well of DMSO and absorbance was read at 490 nm and normalized to DMSO control. All MTT proliferation assays were performed in duplicate and data are represented as mean ± s.e.m. between three independent experiments unless otherwise indicated in the figure legend.

Immunohistochemistry

Immunohistochemical (IHC) staining assays were carried out on 4-mm thick FFPE tissue sections. Tissue sections were deparaffinized three times by xylene and then hydrated in water for 15 min. Hydrogen peroxide (0.6%) was used to eliminate endogenous peroxidase activity. The sections were blocked with goat serum in Tris-buffered saline for 30 min. Sections were then incubated with anti-CK19 antibody (ab52625, Abcam), anti-S100P antibody (ab133554, Abcam) and anti-CK17 antibody (ab109725, Abcam) overnight at 4°C. Secondary antibody was then applied and incubated at 37℃ for 1 h. Sections were developed with diaminobenzidine and stopped with water. Photographs of representative fields were captured using a Leica CCD camera DFC420 connected to a Leica DMIRE2 microscope (Leica Microsystems Imaging Solutions, Cambridge, UK).

The expressions of S100P and KRT17 were examined by two pathologists, who were blinded to the clinical data of the patients. The pathologists inspected the complete tumor sections at 100 and 200 magnifications. The staining intensity was determined as 0 (absent), 1 (weak), 2 (moderate), and 3 (strong). The expression levels of S100P and KRT17 were semi-quantified using an IHC score calculated by multiplication of the staining intensity (0-3) with the percentage of positive tumor cells (0-100%). The ranges of IHC scores for each biomarker in tumor tissue are as follows: 0-225 for S100P (median 95), 0-285 for KRT17 (median 130). The cut-off IHC scores that were considered positive are 70 for S100P and 90 for KRT17. The cases with IHC scores below the cut-off values

were considered negative.

Follow-up

The patients of the internal validation cohort were followed-up using routine blood tests, physical examination, and abdominal ultrasonography every two months in the first two years and every three months thereafter at our hospital after operations. The primary endpoint was overall survival (OS). OS was defined as the interval between the dates of surgery and death. The follow-up information of other cohorts was available in the supplementary data of the original studies, respectively.

Supplementary Figure legends

Figure S1 The most recurrently mutated genes of ICC in the discovery cohort

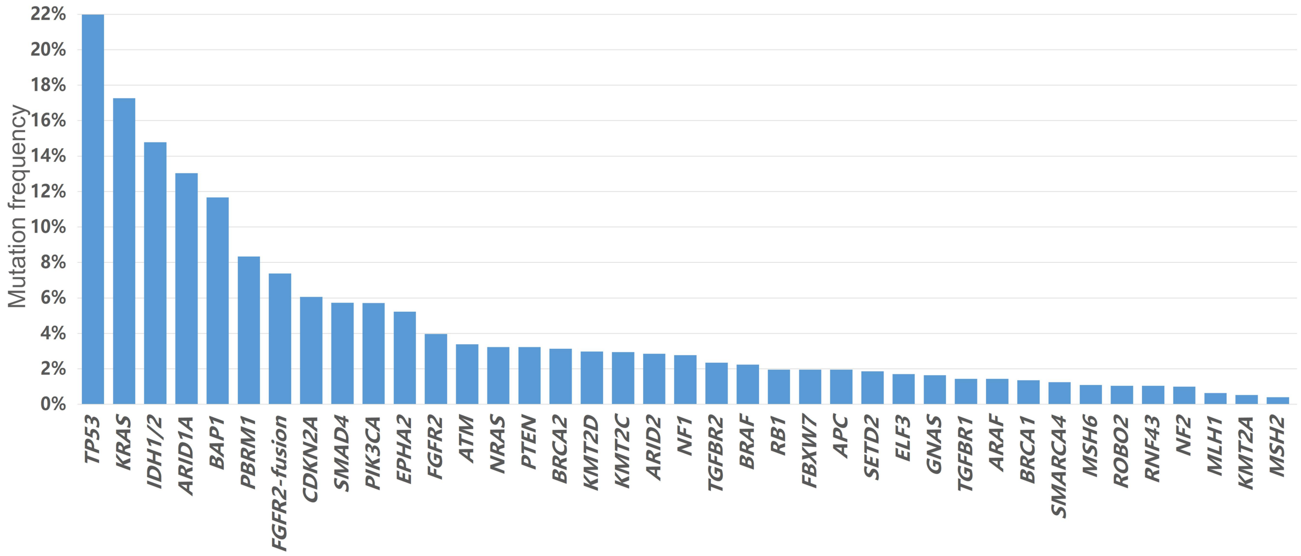

Figure S2 TMB for ICC between different mutational clusters in the WES/WGS cohort

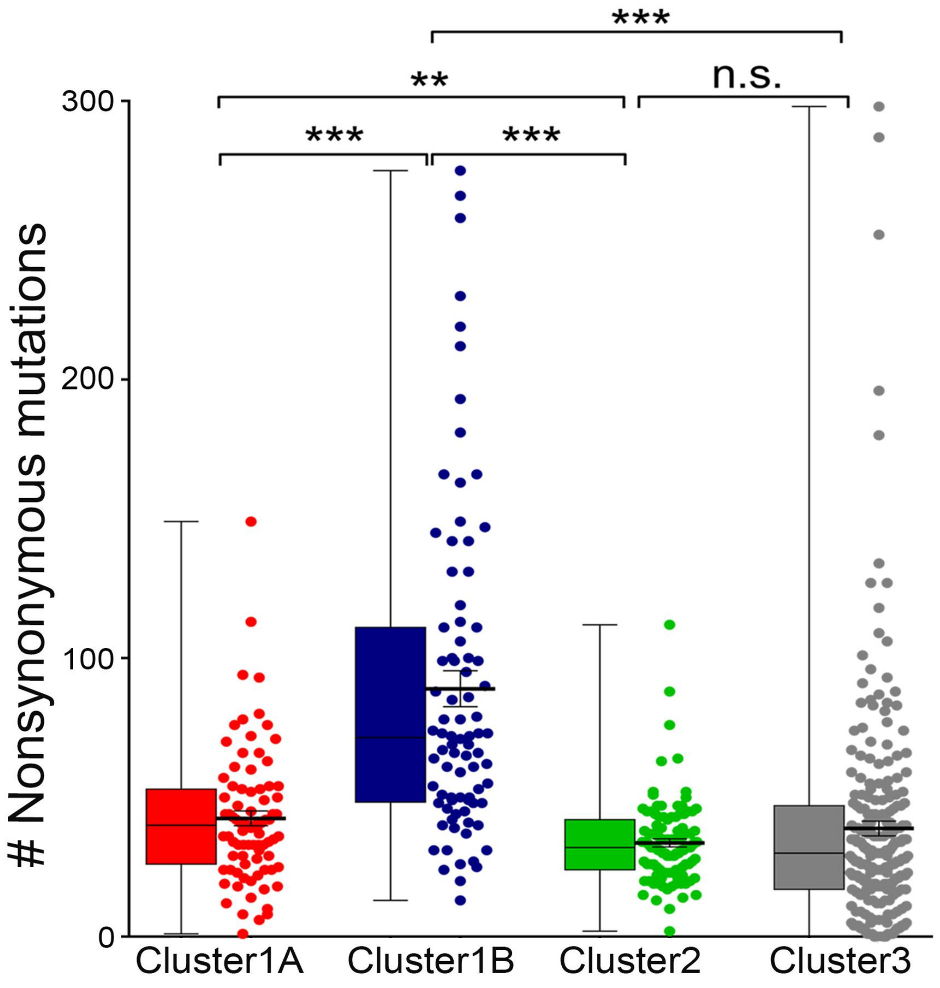

Figure S3 Validation of the robustness of the proposed mutational cluster in independent ICC cohorts. The co-mutation was calculated by chi-square test

(A) STS cohort in the discovery dataset including 263 ICC cases.

(B) Internal validation cohort including 225 ICC cases.

(C) External validation cohort including 212 ICC cases from Western countries using NGS panel from the GENIE database.

(D) External validation cohort including 239 ICC cases from Asian countries (mostly Thailand) using NGS panels from the TIGER-LC and ICGC studies.

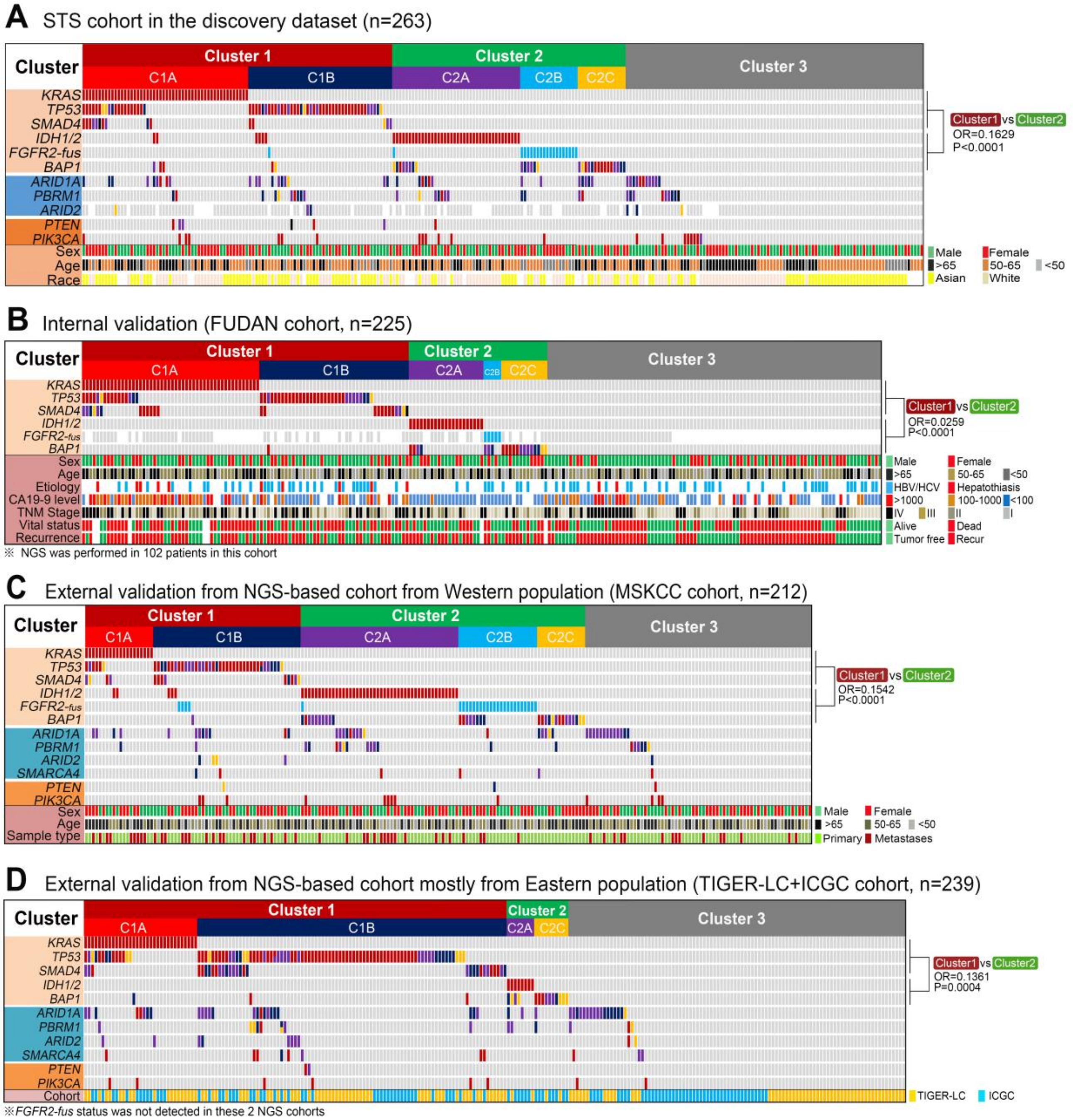

Figure S4 TMB between different mutational clusters from the validation cohorts

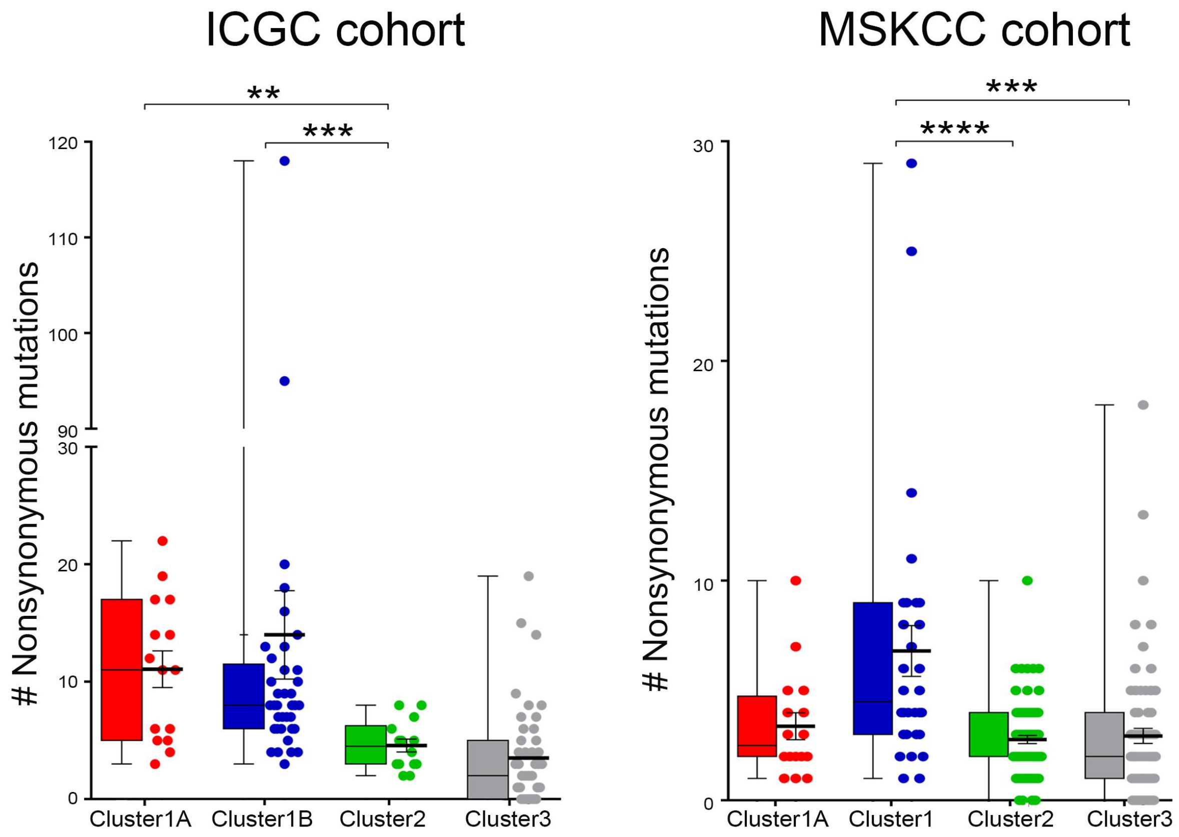

Figure S5 Association of patient survival with different mutational clusters in ICC

(A) OS from the combined cohort showed different prognosis between mutational clusters in ICC.

(B) RFS from the combined cohort showed different prognosis between mutational clusters in ICC.

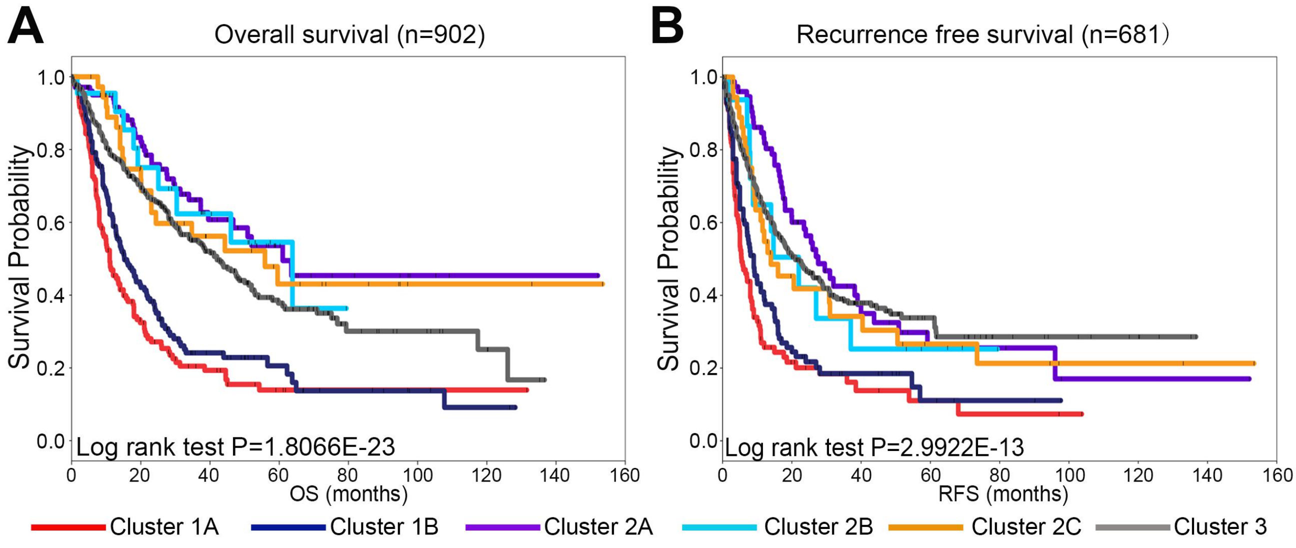

Figure S6 Survival analyses from multi-center cohorts showed different prognosis (OS and RFS) between different mutational clusters

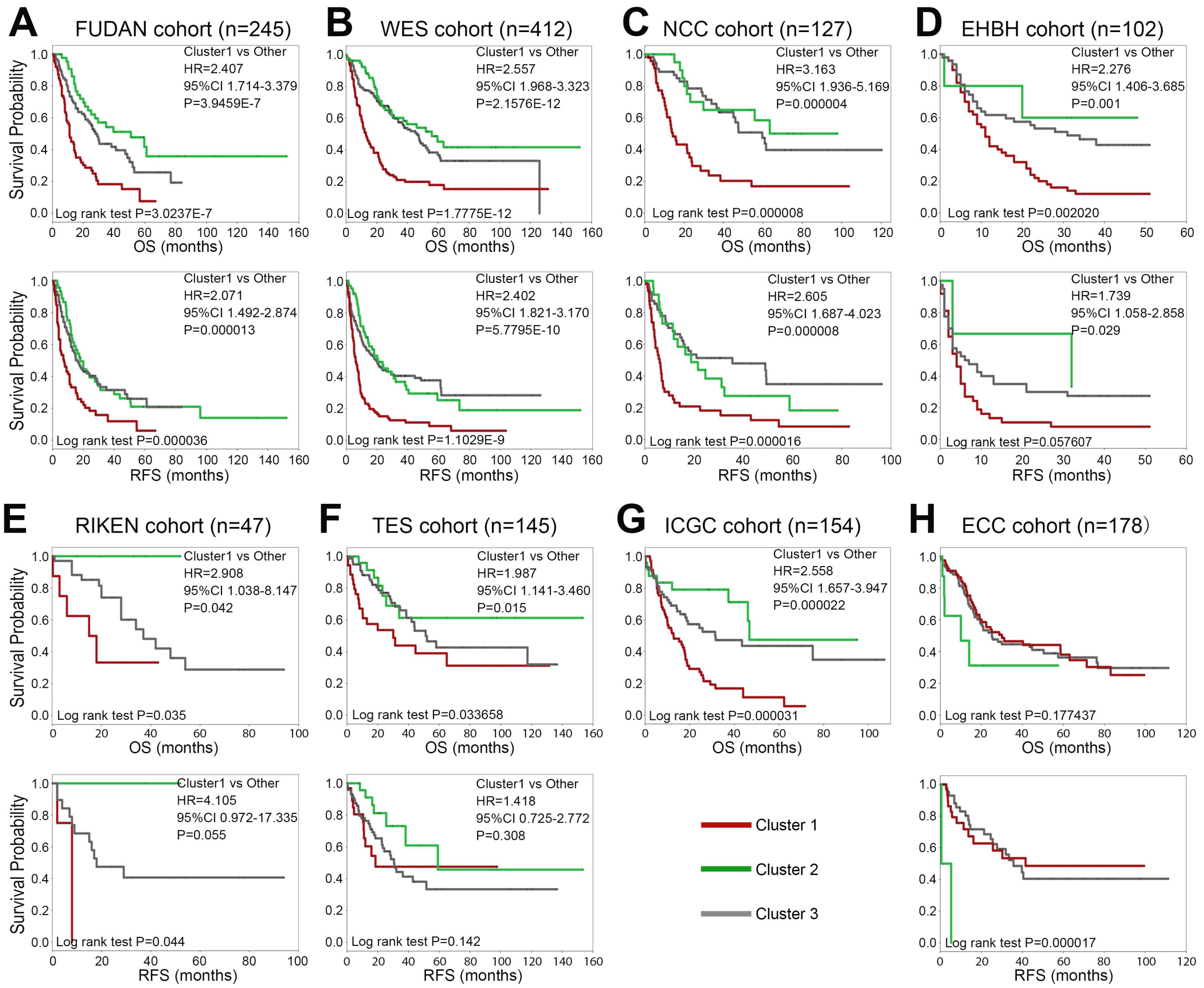

Figure S7 345 ICCs from China (FUDAN and EHBH cohort) were enrolled for univariate (A) and multivariate (B) Cox regression test. Multivariate analysis confirmed the prognostic value of Cluster1 even after accounting for CA19-9 level, clinical staging.

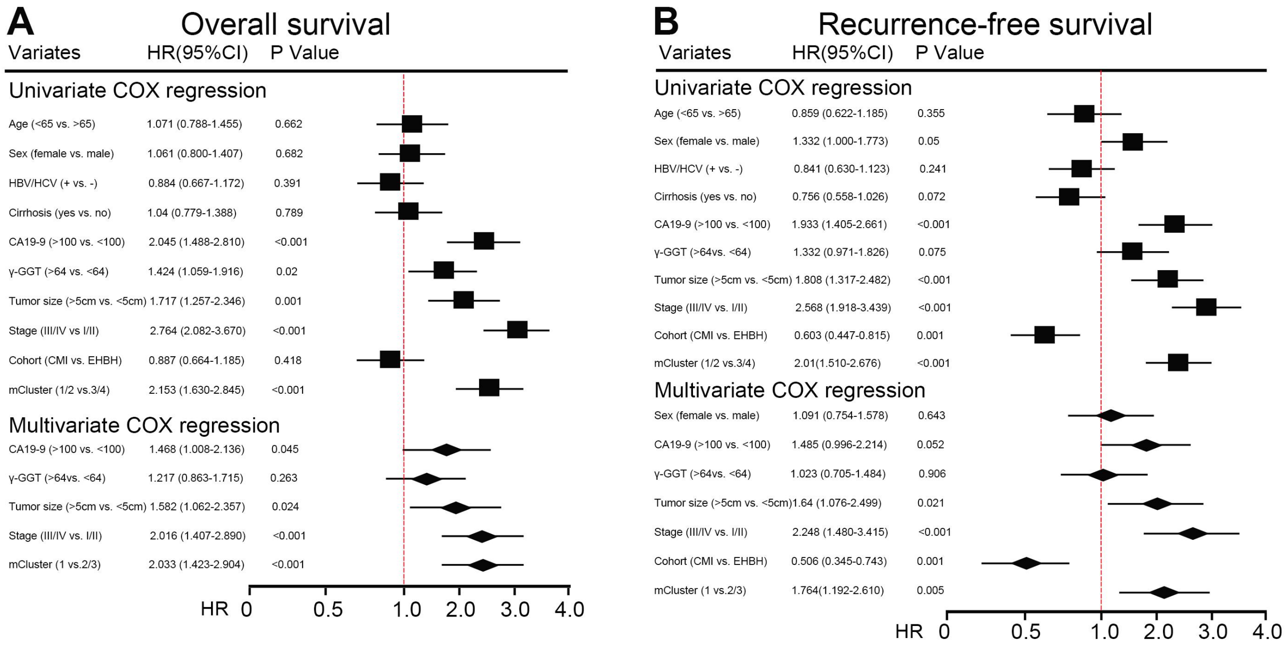

Figure S8 Correlation of different clusters with ICC cell biological behavior

(A) The distribution of mutational clusters in CCA cell lines.

(B) Proliferation ability among ICC cell lines with different mutational clusters.

(C) Tumorigenic ability *in vivo* among ICC cell lines with different mutational clusters (n = 5). Data represent means±SEM. *$P < 0.05$, **$P < 0.01$ and ***$P < 0.001$, Student's t-test.

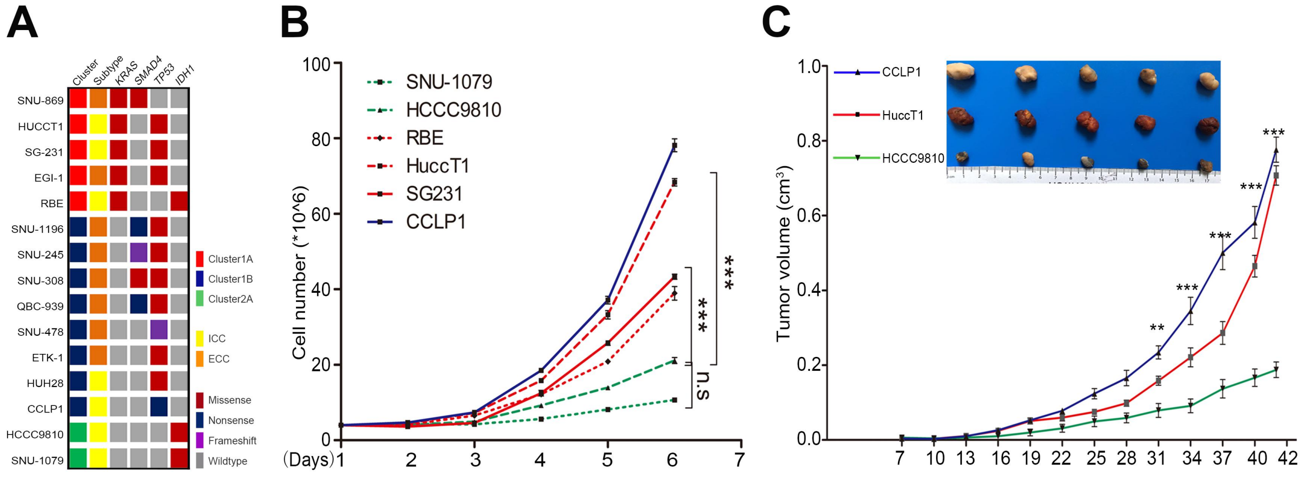

Figure S9 Analysis of ICC progression related genes expression among different mutational clusters. Statistical significance was determined by Mann-Whitney test (2 tailed).

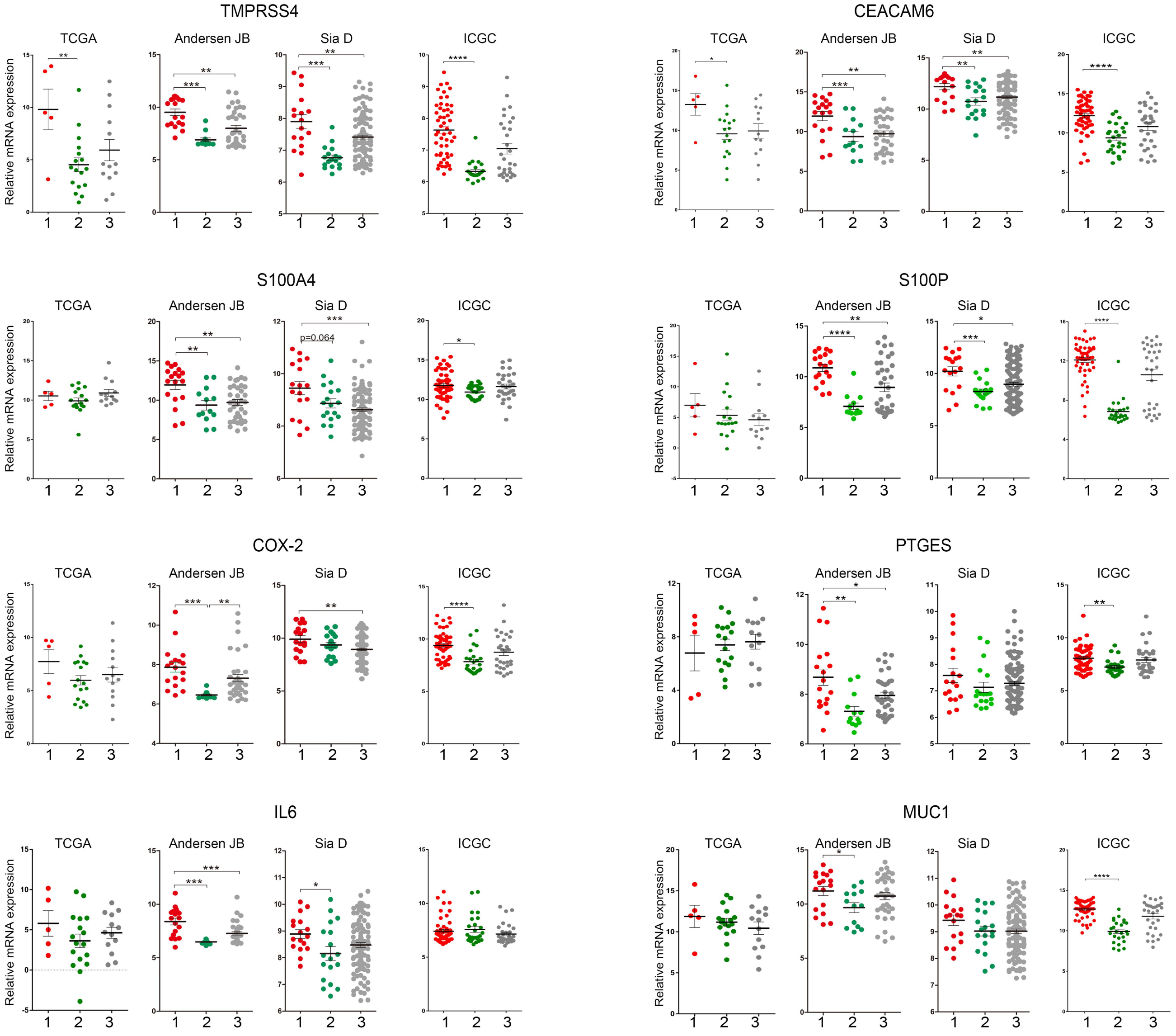

Figure S10 (A) Analysis of PDGFD and FGFR2/3/4 gene expression among different mutational clusters. Statistical significance was determined by Mann-Whitney test (2 tailed). (B) FGFR2/3/4 pathway activation exist across the Cluster 2 ICCs.

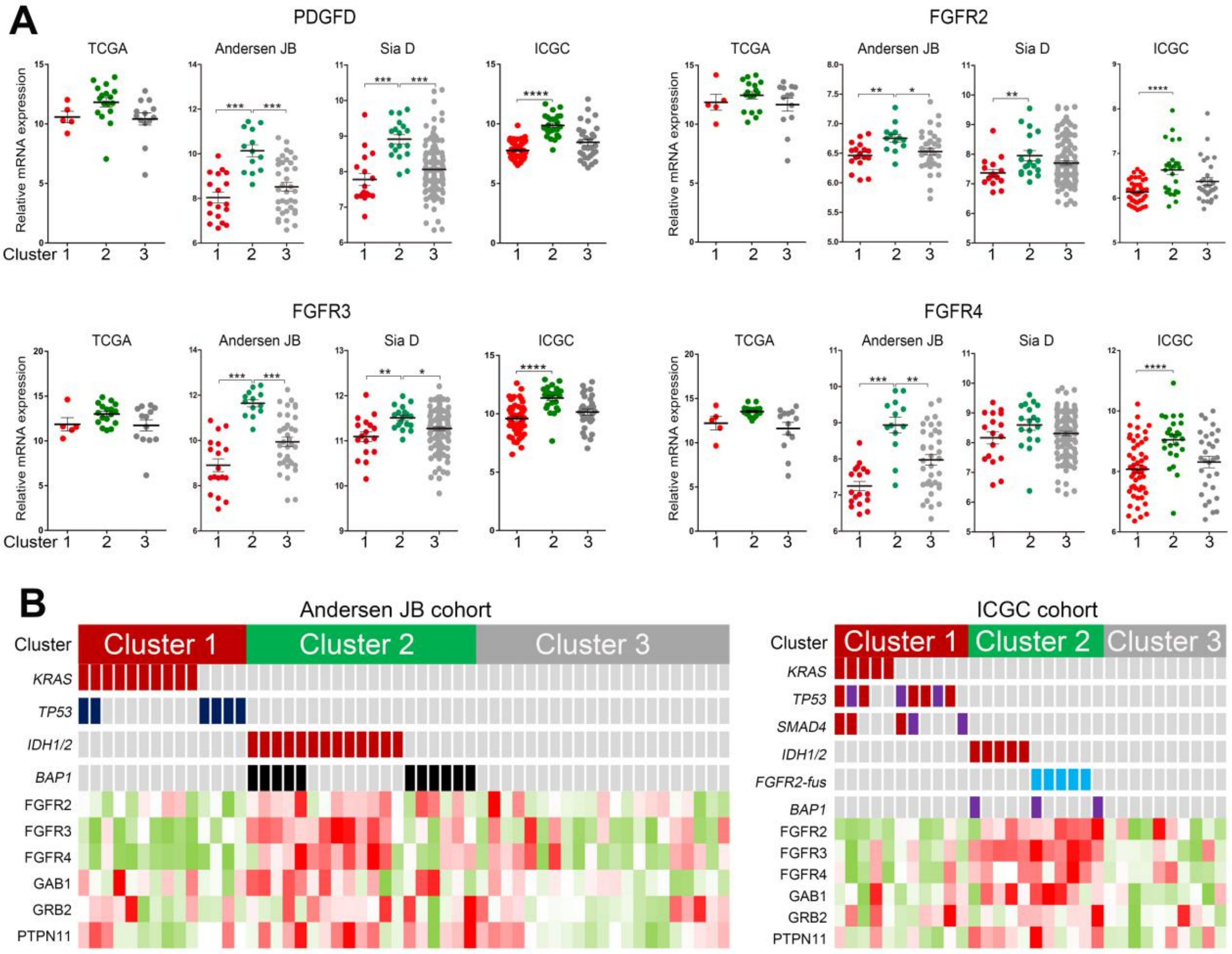

Figure S11 Correlation between S100P and KRT17 mRNA expression in ICC from 9 gene expression profiling datasets.

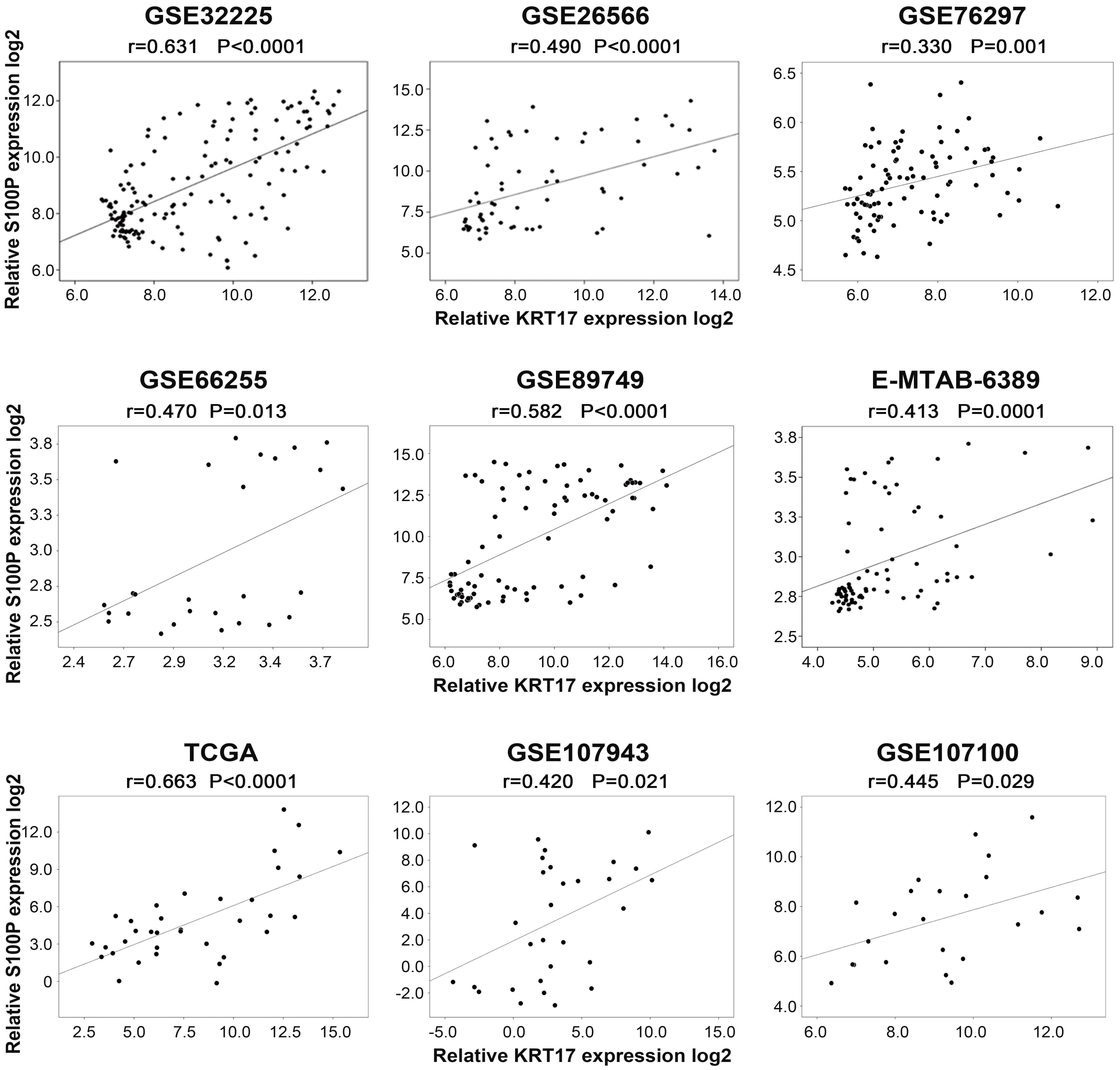

Figure S12 Impact of S100P/KRT17 mRNA expression on OS in ICC.

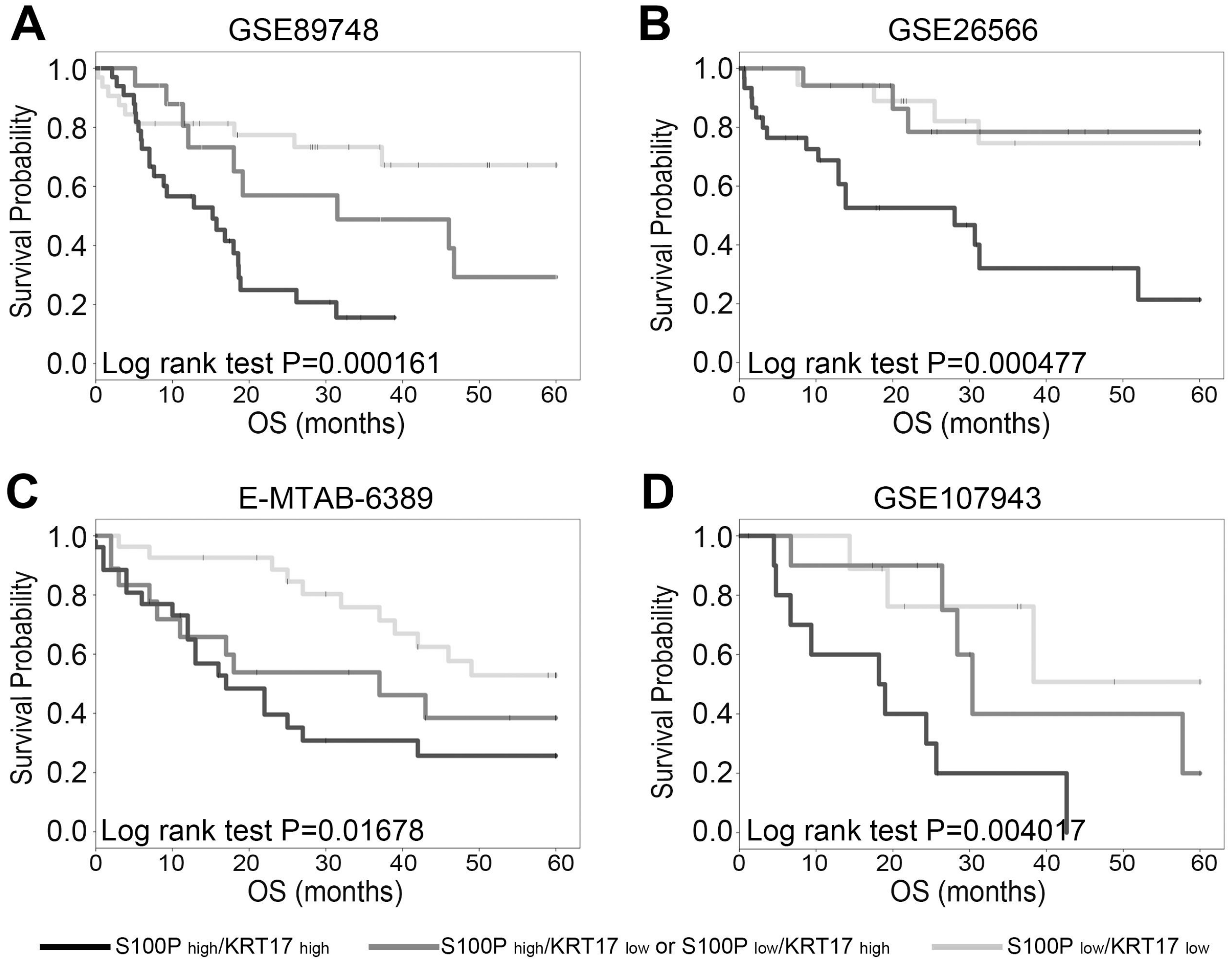

Figure S13 The distribution of S100P/KRT17 expression status (double positive, single positive or double negative) among different mutational clusters of ICC by immunohistochemistry

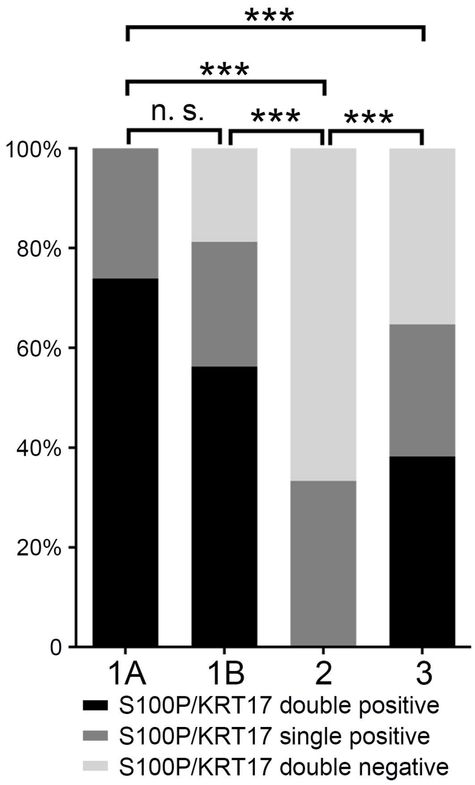

Figure S14 The diagnostic performance between CP score and CA19-9 alone for distinguishing Cluster1 ICCs from Cluster2 ICCs was compared through the analyses of receiver operating characteristic (ROC) curves (n = 94)

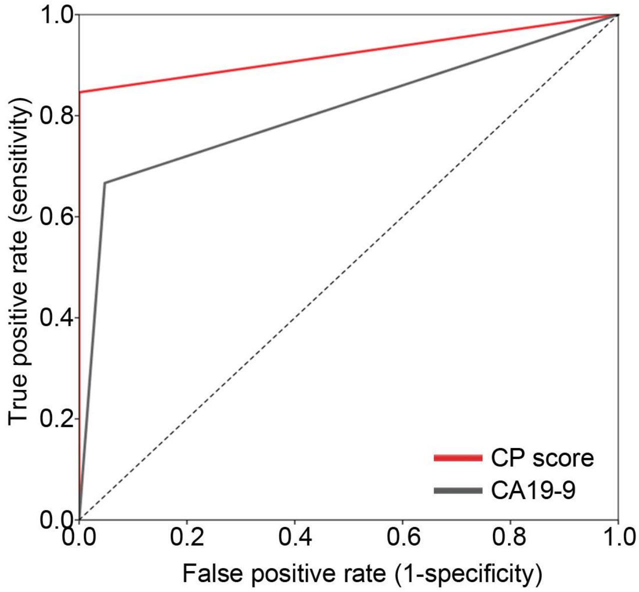

Area under receiver operating characteristic curve (AUROC)					
	AUC	SE	P value	95% CI	
				Lower	Up
CP score	0.923	0.036	<0.0001	0.853	0.993
CA19-9	0.810	0.056	<0.0001	0.699	0.920

Table S2: The clinico-pathological characteristics of ICC patients in the discovery and validation cohorts

Clinical variable		WES/WGS cohort N = 505	FUDAN cohort N = 225	MSKCC cohort N = 212	TIGER-LC cohort N = 129	ICGC cohort N = 110	ECC cohort N = 178
**Demographic**							
Sex							
	Male	270	145	96	43	65	124
	Female	195	80	116	86	45	54
Age (year)							
	Median	62	61	63	N.A.	59.5	63
	≥ 60	267	116	135	N.A.	55	109
	< 60	192	109	77	N.A.	55	66
Race							
	Asia	385	225	N.A.	N.A.	77	145
	White	116	0	N.A.	N.A.	33	33
	Black	4	0	N.A.	N.A.	0	0
**Etiology**							
Smoking							
	Never	135	N.A.	N.A.	N.A.	N.A.	N.A.
	Ever	50	N.A.	N.A.	N.A.	N.A.	N.A.
T2DM							
	No	120	N.A.	N.A.	N.A.	N.A.	N.A.
	Yes	15	N.A.	N.A.	N.A.	N.A.	N.A.
Alcohol consumption							
	Yes	23	N.A.	N.A.	N.A.	N.A.	N.A.
	No	118	N.A.	N.A.	N.A.	N.A.	N.A.
HBV status							
	Positive	99	75	N.A.	N.A.	6	1
	Negative	395	150	N.A.	N.A.	62	124
HCV status							
	Positive	24	2	N.A.	N.A.	3	1
	Negative	470	223	N.A.	N.A.	62	123
Liver fluke infection							

	Positive	14	1	N.A.	N.A.	60	55
	Negative	462	224	N.A.	N.A.	50	123
Hepatolithiasis							
	Yes	7	13	N.A.	N.A.	N.A.	N.A.
	No	469	212	N.A.	N.A.	N.A.	N.A.
Other		3	0	N.A.	N.A.	2	N.A.
**Tumor features**							
CA19-9							
	Normal, ≤ 100U/mL	103	110	N.A.	N.A.	N.A.	N.A.
	Abnormal, > 100U	54	78	N.A.	N.A.	N.A.	N.A.
Tumor size							
	≤ 5 cm	116	125	N.A.	N.A.	N.A.	N.A.
	> 5 cm	125	100	N.A.	N.A.	N.A.	N.A.
Tumor number							
	Single	89	188	N.A.	N.A.	N.A.	N.A.
	Multinodular	41	37	N.A.	N.A.	N.A.	N.A.
Vascular invasion							
	No	153	161	N.A.	N.A.	N.A.	N.A.
	Yes	69	56	N.A.	N.A.	N.A.	N.A.
Perinural invasion							
	No	62	186	N.A.	N.A.	N.A.	N.A.
	Yes	13	31	N.A.	N.A.	N.A.	N.A.
Tumor differentiation							
	Well	49	12	N.A.	N.A.	61	105
	Moderate	276	124	N.A.	N.A.	36	54
	Poor	59	81	N.A.	N.A.	12	15
TNM staging							
	I	103	74	N.A.	N.A.	11	33
	II	103	33	N.A.	N.A.	11	60
	III	67	42	N.A.	N.A.	19	49
	IV	146	76	N.A.	N.A.	46	33
Resection performed							
	Yes	487	217	N.A.	N.A.	110	78

	No	18	8	N.A.	N.A.	0	0
Clinical outcome							
	Median OS (months)	28.4	15	18.78	N.A.	18.1	19.23
	Median RFS (months)	11.9	8	N.A.	N.A.	N.A.	29.97

Abbreviations: N.A., not available; T2DM, type 2 diabetes mellitus; HBV, hepatitis B virus; HCV, hepatitis C virus; ECC, extrahepatic cholangiocarcinoma

Each value represents the number of patients

Table S3: Targeted Gene Panels

Panel A: List of the 381 genes targeted for sequencing

ABL1	ABL2	ACVR1B	ACVR2A	ADAM29	ADGRA2	AKT1	AKT2	AKT3	**ALK**
AMER1	APC	AR	ARAF	ARFRP1	ARID1A	ARID1B	ARID2	ASXL1	ATM
ATR	ATRX	AURKA	AURKB	AXIN1	AXL	BAP1	BARD1	BCL2	BCL2L1
BCL2L11	BCL2L2	BCL6	BCOR	BCORL1	**BCR**	BIRC5	BLK	BLM	BMX
**BRAF**	**BRCA1**	**BRCA2**	**BRD4**	BRIP1	BTG1	BTK	C11orf30	CARD11	CBFB
CBL	CCND1	CCND2	CCND3	CCNE1	CD274	CD79A	CD79B	CDC73	CDH1
CDK12	CDK4	CDK6	CDK8	CDKN1A	CDKN1B	CDKN2A	CDKN2B	CDKN2C	CEBPA
CHD2	CHD4	CHEK1	CHEK2	CIC	CRBN	CREBBP	CRKL	CRLF2	CSF1R
CSK	CSNK1A1	CTCF	CTNNA1	CTNNB1	CUL3	CXCR4	CYLD	CYP2C19	CYP2D6
DAXX	DDR1	**DDR2**	DICER1	DNMT3A	DOT1L	DPYD	EGF	**EGFR**	EP300
EPHA2	EPHA3	EPHA5	EPHA7	EPHB1	ERBB2	ERBB3	ERBB4	ERCC1	ERG
ERRFI1	ESR1	**ETV1**	**ETV4**	**ETV5**	**ETV6**	EZH2	FAM135B	FAM46C	FANCA
FANCC	FANCD2	FANCE	FANCF	FANCG	FANCL	FAS	FAT1	FBXW7	FGF10
FGF14	FGF19	FGF23	FGF3	FGF4	FGF6	**FGFR1**	**FGFR2**	**FGFR3**	FGFR4
FGR	FH	FLCN	FLT1	FLT3	FLT4	FOXL2	FOXP1	FRS2	FUBP1
FYN	GABRA6	GATA1	GATA2	GATA3	GATA4	GATA6	GID4	GLI1	GLI2
GLI3	GNA11	GNA13	GNAQ	GNAS	GRIN2A	GRM3	GSK3B	H3F3A	HCK
HGF	HNF1A	HRAS	HSD3B1	HSP90AA1	IDH1	IDH2	IGF1R	IGF2	IKBKE
IKZF1	IL7R	INHBA	INPP4B	IRF2	IRF4	IRS2	ITK	JAK1	JAK2
JAK3	JUN	KAT6A	KDM5A	KDM5C	KDM6A	KDR	KEAP1	KEL	KIT
KLHL6	KMT2A	KMT2C	KMT2D	KRAS	LCK	LIMK1	LMO1	LRP1	LRP1B
LYN	LZTR1	MAGI2	MAP2K1	MAP2K2	MAP2K4	MAP3K1	MAP4K5	MCL1	MDM2
MDM4	MED12	MEF2B	MEN1	**MET**	MITF	MLH1	MPL	MRE11A	MS4A1

MSH2	*MSH6*	*MST1R*	*MTOR*	*MUTYH*	***MYB***	*MYC*	*MYCL*	*MYCN*	*MYD88*
NEK11	*NF1*	*NF2*	*NFE2L2*	*NFKBIA*	*NKX2-1*	*NOTCH1*	***NOTCH2***	*NOTCH3*	*NPM1*
NRAS	*NRG1*	*NRG3*	*NSD1*	***NTRK1***	***NTRK2***	*NTRK3*	*NUP93*	*PAK3*	*PALB2*
PARK2	*PAX5*	*PBRM1*	*PDCD1LG2*	***PDGFRA***	*PDGFRB*	*PDK1*	*PIK3C2B*	*PIK3CA*	*PIK3CB*
PIK3CD	*PIK3CG*	*PIK3R1*	*PIK3R2*	*PKD2*	*PLA2G1B*	*PLCG2*	*PMS2*	*POLD1*	*POLE*
PPP2R1A	*PRDM1*	*PREX2*	*PRKAR1A*	*PRKCI*	*PRKDC*	*PRSS8*	*PTCH1*	*PTEN*	*PTK2*
PTK6	*PTPN11*	*QKI*	*RAC1*	*RAD50*	*RAD51*	***RAF1***	*RANBP2*	***RARA***	*RB1*
RBM10	***RET***	*RICTOR*	*RIT1*	*RNF43*	*ROCK1*	*ROCK2*	***ROS1***	*RPTOR*	*RUNX1*
RUNX1T1	*RXRA*	*SDHA*	*SDHB*	*SDHC*	*SDHD*	*SETD2*	*SF3B1*	*SIK1*	*SLIT2*
SMAD2	*SMAD3*	*SMAD4*	*SMARCA2*	*SMARCA4*	*SMARCB1*	*SMO*	*SNCAIP*	*SOCS1*	*SOX10*
SOX2	*SOX9*	*SPEN*	*SPOP*	*SPTA1*	*SRC*	*SRMS*	*STAG2*	*STAT3*	*STAT4*
STK11	*STK24*	*SUFU*	*SYK*	*TAF1*	*TBX3*	*TCF7L2*	*TEK*	*TERT*	*TET2*
TGFBR1	*TGFBR2*	*TIE1*	***TMPRSS2***	*TNFAIP3*	*TNFRSF14*	*TNFSF11*	*TNK2*	*TOP1*	*TOP2A*
TP53	*TPMT*	*TSC1*	*TSC2*	*TSHR*	*TYK2*	*U2AF1*	*UGT1A1*	*VEGFA*	*VHL*
WEE1	*WEE2*	*WISP3*	*WT1*	*XIAP*	*XPO1*	*YES1*	*ZBTB2*	*ZNF217*	*ZNF703*
ZNF750									
**Panel B: List of the 35 genes targeted for sequencing**									
APC	*ARAF*	*ARID1A*	*ARID2*	*ATM*	*BAP1*	*BRAF*	*BRCA1*	*BRCA2*	*CDKN2A*
ELF3	*EPHA2*	*FBXW7*	***FGFR2***	*GNAS*	*IDH1*	*IDH2*	*KRAS*	*MLH1*	*MSH2*
MSH6	*NF1*	*NF2*	*NRAS*	*PBRM1*	*PIK3CA*	*PTEN*	*RB1*	*RNF43*	*SETD2*
SMAD4	*SMARCA4*	*TGFBR1*	*TGFBR2*	*TP53*					

In addition to the coding exons, fusion status are also captured for genes highlighted in bold

Table S4: Summary of the included gene expression and DNA methylation profile datasets for analyse

Gene expression profile datasets of CCAs			
Cohort	Platforms	Sample number	Data Accession ID
TCGA-CHOL cohort	Illumina HiSeq 2000	28 ICC and 6 ECC	cbioportal
LEC cohort	Illumina humanRef-8 v2.0	68 ICC and 36 ECC	GSE26566
Sia D cohort	Illumina HumanRef-8 WG-DASL V3.0	149 ICC	GSE32225
TIGER-LC cohort	Affymetrix Human Transcriptome Array 2.0	94 ICC	GSE76297

Rhee H cohort	Illumina HumanHT-12 V4.0	27 ICC	GSE66255
ICGC cohort	Illumina HumanHT-12 V4.0	89 ICC and 29 ECC	GSE89748
CIT cohort	Affymetrix Human Transcriptome Array 2.0	78 ICC	E-MTAB-6389
DSMC cohort	Illumina NextSeq 500	30 ICC	GSE107943
Peraldo-Neia cohort	Illumina HumanHT-12 WG-DASL V4.0	24 ICC	GSE107100
DNA methylation profile datasets of ICCs			
Cohort	Platforms	Sample number	Data Accession ID
Fudan/Mayo cohort	Human Methylation450 BeadChip assays	56 ICC	GSE32079
NUS cohort	Human Methylation450 BeadChip assays	56 ICC	GSE49656
ICGC cohort	Human Methylation450 BeadChip assays	88 ICC	GSE89803

Table S5: The list of frequently mutated genes and mutation frequency in the discovery cohort

Gene	Mutation frequency
TP53	**21.99%**
KRAS	**17.27%**
IDH1/2	**14.78%**
ARID1A	**13.04%**
BAP1	**11.68%**
PBRM1	**8.33%**
FGFR2 fusion	**7.38%**
CDKN2A	**6.06%**
SMAD4	**5.73%**
PIK3CA	**5.71%**
EPHA2	**5.23%**
FGFR2	**3.97%**
ATM	**3.39%**
NRAS	**3.23%**
PTEN	**3.23%**
BRCA2	**3.13%**
KMT2D	**2.98%**
KMT2C	**2.94%**

ARID2	**2.85%**
NF1	**2.78%**
TGFBR2	**2.34%**
BRAF	**2.24%**
RB1	1.95%
FBXW7	1.95%
APC	1.95%
SETD2	1.85%
ELF3	1.71%
GNAS	1.64%
TGFBR1	1.44%
ARAF	1.43%
BRCA1	1.36%
SMARCA4	1.25%
MSH6	1.09%
ROBO2	1.04%
RNF43	1.04%
NF2	1.00%
MLH1	0.64%
KMT2A	0.52%
MSH2	0.41%

Genes with mutation frequency > 2% were highlighted in bold

Table S6: List of somatic nonsilent single nucleotide variations and indels of the selected genes in the WES/WGS cohort

Sample ID	Reference	Sequencing method	Gene Symbol	Chromo some	Start position	End position	Reference Allele	Variant Allele	Nucleotide Change	Amino acid change	Mutation type
T7(8-6)	Zou SS *et al*	WES	*IDH1*	2	209113113	209113113	G	A	SNV	R132C	Missense
T5(8-4)	Zou SS *et al*	WES	*TP53*	17	7578410	7578410	T	A	SNV	R174W	Missense
T5(8-4)	Zou SS *et al*	WES	*ARID2*	12	46245473	46245474	-	G	Insertion	G1190 fs	Frameshift
CT99	Zou SS *et al*	WES	*TP53*	17	7577120	7577120	C	T	SNV	R273H	Missense
CT97	Zou SS *et al*	WES	*TP53*	17	7577534	7577534	C	A	SNV	R249S	Missense
CT95	Zou SS *et al*	WES	*TP53*	17	7576927	7576927	C	A	SNV	Splice site	Splice site
CT95	Zou SS *et al*	WES	*TP53*	17	7578419	7578419	C	A	SNV	E171*	Nonsense
CT94	Zou SS *et al*	WES	*PTEN*	10	89690843	89690843	A	T	SNV	R84*	Nonsense
CT91	Zou SS *et al*	WES	*TGFBR2*	3	30691872	30691872	A	-	Deletion	K128 fs	Frameshift

CT90	Zou SS *et al*	WES	*TP53*	17	7577534	7577534	C	A	SNV	R249S	Missense
CT87	Zou SS *et al*	WES	*TP53*	17	7578226	7578226	T	A	SNV	D208V	Missense
CT86	Zou SS *et al*	WES	*EPHA2*	1	16475486	16475486	G	C	SNV	C70W	Missense
CT81	Zou SS *et al*	WES	*BRAF*	7	140453149	140453149	C	G	SNV	G204R	Missense
CT80	Zou SS *et al*	WES	*NRAS*	1	115256529	115256529	T	C	SNV	Q61R	Missense
CT80	Zou SS *et al*	WES	*BAP1*	3	52443757	52443757	G	-	Deletion	L14 fs	Frameshift
CT74	Zou SS *et al*	WES	*KMT2C*	7	151879655	151879655	G	A	SNV	Q1764*	Nonsense
CT71	Zou SS *et al*	WES	*TP53*	17	7577568	7577568	C	A	SNV	C238F	Missense
CT70	Zou SS *et al*	WES	*BRCA2*	13	32914434	32914434	C	T	SNV	A1981V	Missense
CT69	Zou SS *et al*	WES	*PIK3CA*	3	178936091	178936091	G	A	SNV	E545K	Missense
CT66	Zou SS *et al*	WES	*TP53*	17	7578535	7578535	T	C	SNV	K132R	Missense
CT66	Zou SS *et al*	WES	*SMAD4*	18	48593491	48593494	AGAC	-	Deletion	D415 fs	Frameshift
CT66	Zou SS *et al*	WES	*KRAS*	12	25398284	25398284	C	G	SNV	G12A	Missense
CT63	Zou SS *et al*	WES	*TP53*	17	7577538	7577538	C	T	SNV	R248Q	Missense
CT63	Zou SS *et al*	WES	*NF1*	17	29585521	29585521	G	C	SNV	Splice site	Splice site
CT59	Zou SS *et al*	WES	*TP53*	17	7579346	7579348	AAG	-	Deletion	F113 del	Inframe deletion
CT59	Zou SS *et al*	WES	*KRAS*	12	25398284	25398284	C	T	SNV	G12D	Missense
CT58	Zou SS *et al*	WES	*TP53*	17	7578224	7578224	T	A	SNV	R209*	Nonsense
CT56	Zou SS *et al*	WES	*ARID1A*	1	27092812	27092812	G	T	SNV	G945*	Nonsense
CT53	Zou SS *et al*	WES	*EPHA2*	1	16464659	16464660	-	A	Insertion	Y334 fs	Frameshift
CT53	Zou SS *et al*	WES	*ARID1A*	1	27105550	27105550	C	T	SNV	R1721*	Nonsense
CT51	Zou SS *et al*	WES	*TP53*	17	7577534	7577534	C	A	SNV	R249S	Missense
CT51	Zou SS *et al*	WES	*KMT2C*	7	151904477	151904477	G	C	SNV	S1250C	Missense
CT46	Zou SS *et al*	WES	*PBRM1*	3	52668666	52668666	G	-	Deletion	P418 fs	Frameshift
CT46	Zou SS *et al*	WES	*KRAS*	12	25398284	25398284	C	T	SNV	G12D	Missense
CT46	Zou SS *et al*	WES	*KMT2C*	7	151935871	151935871	C	A	SNV	W858L	Missense
CT46	Zou SS *et al*	WES	*IDH1*	2	209113113	209113113	G	A	SNV	R132C	Missense
CT44	Zou SS *et al*	WES	*PTEN*	10	89690847	89690847	G	A	SNV	Splice site	Splice site
CT42	Zou SS *et al*	WES	*IDH1*	2	209113113	209113113	G	A	SNV	R132C	Missense
CT41	Zou SS *et al*	WES	*TP53*	17	7577534	7577534	C	A	SNV	R249S	Missense
CT41	Zou SS *et al*	WES	*PTEN*	10	89717672	89717672	C	T	SNV	R233*	Nonsense
CT34	Zou SS *et al*	WES	*TP53*	17	7577509	7577509	C	G	SNV	E258Q	Missense
CT34	Zou SS *et al*	WES	*KMT2D*	12	49426729	49426729	A	T	SNV	L3921Q	Missense
CT34	Zou SS *et al*	WES	*BRAF*	7	140453154	140453154	T	C	SNV	D202G	Missense
CT34	Zou SS *et al*	WES	*ATM*	11	108121588	108121588	C	T	SNV	Q466*	Nonsense

CT34	Zou SS *et al*	WES	*ATM*	11	108121601	108121601	C	T	SNV	S470L	Nonsense
CT33	Zou SS *et al*	WES	*KRAS*	12	25398284	25398284	C	A	SNV	G12V	Missense
CT29	Zou SS *et al*	WES	*KRAS*	12	25398284	25398284	C	A	SNV	G12V	Missense
CT28	Zou SS *et al*	WES	*TP53*	17	7577534	7577534	C	A	SNV	R249S	Missense
CT28	Zou SS *et al*	WES	*ARID1A*	1	27106804	27106804	C	-	Deletion	F2141 fs	Frameshift
CT26	Zou SS *et al*	WES	*TP53*	17	7578177	7578177	C	A	SNV	E224D	Missense
CT26	Zou SS *et al*	WES	*KMT2C*	7	151945241	151945241	T	A	SNV	I760L	Missense
CT20	Zou SS *et al*	WES	*TP53*	17	7577534	7577534	C	A	SNV	R249S	Missense
CT17	Zou SS *et al*	WES	*TP53*	17	7578404	7578404	A	T	SNV	C176S	Missense
CT17	Zou SS *et al*	WES	*KRAS*	12	25398284	25398284	C	T	SNV	G12D	Missense
CT16	Zou SS *et al*	WES	*TP53*	17	7574035	7574035	T	A	SNV	Splice site	Splice site
CT16	Zou SS *et al*	WES	*PIK3CA*	3	178937402	178937402	A	C	SNV	Q597P	Missense
CT16	Zou SS *et al*	WES	*KMT2D*	12	49431119	49431119	C	A	SNV	Q3340H	Missense
CT16	Zou SS *et al*	WES	*ARID2*	12	46125092	46125092	C	-	Deletion	L94 fs	Frameshift
CT145	Zou SS *et al*	WES	*TP53*	17	7578235	7578235	T	C	SNV	Y205C	Missense
CT145	Zou SS *et al*	WES	*PTEN*	10	89692794	89692794	A	G	SNV	H93R	Missense
CT144	Zou SS *et al*	WES	*KRAS*	12	25398284	25398284	C	A	SNV	G12V	Missense
CT143	Zou SS *et al*	WES	*TP53*	17	7578530	7578530	A	G	SNV	F134L	Missense
CT143	Zou SS *et al*	WES	*NF1*	17	29554566	29554566	G	A	SNV	W784*	Nonsense
CT141	Zou SS *et al*	WES	*TP53*	17	7577534	7577534	C	A	SNV	R249S	Missense
CT140	Zou SS *et al*	WES	*TP53*	17	7579311	7579311	C	A	SNV	Splice site	Splice site
CT140	Zou SS *et al*	WES	*BRCA2*	13	32954039	32954039	C	T	SNV	Q3036*	Nonsense
CT137	Zou SS *et al*	WES	*KRAS*	12	25398284	25398284	C	A	SNV	G12V	Missense
CT137	Zou SS *et al*	WES	*ATM*	11	108203492	108203492	C	T	SNV	R2598*	Nonsense
CT135	Zou SS *et al*	WES	*TP53*	17	7577018	7577018	C	A	SNV	Splice site	Splice site
CT135	Zou SS *et al*	WES	*SMAD4*	18	48591888	48591888	G	T	SNV	D351Y	Missense
CT134	Zou SS *et al*	WES	*TP53*	17	7577547	7577547	C	T	SNV	G245D	Missense
CT134	Zou SS *et al*	WES	*TP53*	17	7574017	7574017	C	A	SNV	R337L	Missense
CT134	Zou SS *et al*	WES	*PTEN*	10	89711966	89711966	T	C	SNV	F195S	Missense
CT134	Zou SS *et al*	WES	*KRAS*	12	25398284	25398284	C	G	SNV	G12A	Missense
CT133	Zou SS *et al*	WES	*SMAD4*	18	48591919	48591919	G	A	SNV	R361H	Missense
CT133	Zou SS *et al*	WES	*KRAS*	12	25398285	25398285	C	A	SNV	G12C	Missense
CT133	Zou SS *et al*	WES	*APC*	5	112175752	112175752	T	-	Deletion	L1488 fs	Frameshift
CT132	Zou SS *et al*	WES	*IDH1*	2	209113113	209113113	G	A	SNV	R132C	Missense
CT131	Zou SS *et al*	WES	*TP53*	17	7578239	7578239	C	A	SNV	E204*	Nonsense

CT131	Zou SS *et al*	WES	*KMT2B*	19	36213507	36213507	A	T	SNV	H870L	Missense
CT130	Zou SS *et al*	WES	*TP53*	17	7577534	7577534	C	A	SNV	R249S	Missense
CT13	Zou SS *et al*	WES	*TP53*	17	7577534	7577534	C	A	SNV	R249S	Missense
CT13	Zou SS *et al*	WES	*PTEN*	10	89653867	89653867	G	A	SNV	Splice site	Splice site
CT13	Zou SS *et al*	WES	*EPHA2*	1	16464609	16464609	G	A	SNV	P351S	Missense
CT129	Zou SS *et al*	WES	*TP53*	17	7578406	7578406	C	T	SNV	R175H	Missense
CT129	Zou SS *et al*	WES	*KRAS*	12	25398284	25398284	C	T	SNV	G12D	Missense
CT128	Zou SS *et al*	WES	*TP53*	17	7573996	7573996	A	C	SNV	L344R	Missense
CT127	Zou SS *et al*	WES	*TP53*	17	7577498	7577498	C	A	SNV	Splice site	Splice site
CT125(T6)(8-5)	Zou SS *et al*	WES	*TP53*	17	7577534	7577534	C	A	SNV	R249S	Missense
CT123(T3)(8-3)	Zou SS *et al*	WES	*TP53*	17	7573976	7573976	T	C	SNV	K351E	Missense
CT123(T3)(8-3)	Zou SS *et al*	WES	*NF1*	17	29483057	29483071	CAAGGAA TGTCTAAT	-	Deletion	K40_I44 del	Inframe deletion
CT122	Zou SS *et al*	WES	*NF1*	17	29685499	29685499	C	T	SNV	H2658Y	Missense
CT122	Zou SS *et al*	WES	*IDH1*	2	209113112	209113112	C	A	SNV	R132L	Missense
CT121	Zou SS *et al*	WES	*TP53*	17	7578440	7578440	T	A	SNV	K164*	Nonsense
CT120	Zou SS *et al*	WES	*KRAS*	12	25398285	25398285	C	A	SNV	G12C	Missense
CT113	Zou SS *et al*	WES	*TP53*	17	7579406	7579406	G	T	SNV	S94*	Nonsense
CT111	Zou SS *et al*	WES	*TGFBR2*	3	30713853	30713853	G	A	SNV	C393Y	Missense
CT111	Zou SS *et al*	WES	*KRAS*	12	25398284	25398284	C	T	SNV	G12D	Missense
CT111	Zou SS *et al*	WES	*ARID1A*	1	27106504	27106504	C	T	SNV	Q2039*	Nonsense
CT109(45)	Zou SS *et al*	WES	*PIK3CA*	3	178952085	178952085	A	G	SNV	H1047R	Missense
CT108	Zou SS *et al*	WES	*TP53*	17	7577559	7577559	G	A	SNV	S241F	Missense
CT108	Zou SS *et al*	WES	*PIK3CA*	3	178952085	178952085	A	G	SNV	H1047R	Missense
CT108	Zou SS *et al*	WES	*KRAS*	12	25398284	25398284	C	A	SNV	G12V	Missense
CT108	Zou SS *et al*	WES	*ARID1A*	1	27100292	27100292	G	C	SNV	Splice site	Splice site
CT108	Zou SS *et al*	WES	*ARID1A*	1	27100082	27100082	G	A	SNV	G1293E	Missense
CT107	Zou SS *et al*	WES	*KRAS*	12	25398284	25398284	C	A	SNV	G12V	Missense
CT10	Zou SS *et al*	WES	*ARID1A*	1	27057766	27057766	C	T	SNV	Q492*	Nonsense
CT08	Zou SS *et al*	WES	*TP53*	17	7578493	7578493	C	T	SNV	W146*	Nonsense
CT08	Zou SS *et al*	WES	*PTEN*	10	89720857	89720857	C	G	SNV	Y336*	Nonsense
CT07	Zou SS *et al*	WES	*KRAS*	12	25398284	25398284	C	T	SNV	G12D	Missense
CT05	Zou SS *et al*	WES	*TP53*	17	7577568	7577568	C	A	SNV	C238F	Missense
CT03	Zou SS *et al*	WES	*TP53*	17	7578527	7578527	A	-	Deletion	C135 fs	Frameshift

CT03	Zou SS *et al*	WES	*SMAD4*	18	48591930	48591930	G	A	SNV	G365S	Missense
CT03	Zou SS *et al*	WES	*KRAS*	12	25398284	25398284	C	T	SNV	G12D	Missense
CT03	Zou SS *et al*	WES	*BRCA2*	13	32945129	32945129	C	T	SNV	R2842C	Missense
CT03	Zou SS *et al*	WES	*ARID1A*	1	27087458	27087458	C	T	SNV	Q678*	Nonsense
2T-4(8-2)	Zou SS *et al*	WES	*TP53*	17	7577610	7577610	T	A	SNV	Splice site	Splice site
2T-4(8-2)	Zou SS *et al*	WES	*KMT2D*	12	49444860	49444860	T	A	SNV	E869V	Missense
1T-4(8-1)	Zou SS *et al*	WES	*PBRM1*	3	52661288	52661288	C	G	SNV	Splice site	Splice site
pat4-p	Walter D *et al*	WES	*BAP1*	3	NA	NA	NA	NA	NA	N133 fs	Frameshift
pat3-p	Walter D *et al*	WES	*IDH1*	2	209113113	209113113	G	A	SNV	R132C	Missense
pat3-p	Walter D *et al*	WES	*EPHA2*	1	16474910	16474910	G	T	SNV	C262*	Nonsense
pat3-p	Walter D *et al*	WES	*BAP1*	3	NA	NA	NA	NA	NA	Splice site	Splice site
pat2-p	Walter D *et al*	WES	*IDH2*	15	90631839	90631839	T	A	SNV	R172W	Missense
pat2-p	Walter D *et al*	WES	*EPHA2*	1	16456809	16456809	G	A	SNV	R861C	Missense
pat1-p	Walter D *et al*	WES	*KRAS*	12	25398284	25398284	C	G	SNV	G12A	Missense
HCC75	Totoki Y *et al*	WES	*TP53*	17	7577085	7577085	C	T	SNV	E285K	Missense
HCC165	Totoki Y *et al*	WES	*PBRM1*	3	52621474	52621474	GTGGAAT	-	Deletion	T1004 fs	Frameshift
HCC118	Totoki Y *et al*	WES	*IDH2*	15	90631839	90631839	T	A	SNV	R172W	Missense
CCC4	Totoki Y *et al*	WES	*TP53*	17	7577550	7577550	C	T	SNV	G244D	Missense
TCGA-ZU-A 8S4	TCGA	WES	*KMT2C*	7	151935871	151935871	C	A	SNV	W858L	Missense
TCGA-ZU-A 8S4	TCGA	WGS	*CDKN2A*	9						Homo Del	Copy number loss
TCGA-ZU-A 8S4	TCGA	WGS	*BRAF*	7	140453136	140453136	A	T	SNV	V600E	Missense
TCGA-ZU-A 8S4	TCGA	WGS	*ARID1A*	1	27105848	27105852	ATGAT	-	Deletion	N1820 fs	Frameshift
TCGA-ZK-A AYZ	TCGA	WGS	*IDH1*	2	209113113	209113113	G	A	SNV	R132C	Missense
TCGA-ZK-A AYZ	TCGA	WGS	*EPHA2*	1	16474949	16474974	CACTGCAC AGTGCATA CGGGGCTC TT	-	Deletion	E241 fs	Frameshift
TCGA-ZH-A 8Y8	TCGA	WGS	*KRAS*	12	25380277	25380277	G	T	SNV	Q61K	Missense
TCGA-ZH-A 8Y7	TCGA	WGS	*PIK3CA*	3	178916891	178916891	G	A	SNV	R93Q	Missense
TCGA-ZH-A 8Y7	TCGA	WGS	*PBRM1*	3	52696275	52696275	A	T	SNV	Y134*	Nonsense
TCGA-ZH-A	TCGA	WGS	*BRCA2*	13	32911868	32911868	G	A	SNV	E1126K	Missense

8Y7											
TCGA-ZH-A 8Y7	TCGA	WGS	*ARID1A*	1	27101268	27101268	C	-	Deletion	Q1519 fs	Frameshift
TCGA-ZH-A 8Y5	TCGA	WGS	*FGFR2*	10					Fusion	FGFR2-BICC1	Fusion
TCGA-ZH-A 8Y4	TCGA	WGS	*PBRM1*	3	52662939	52662939	G	A	SNV	R472*	Nonsense
TCGA-ZH-A 8Y4	TCGA	WGS	*BAP1*	3	52437221	52437221	T	-	Deletion	D608 fs	Frameshift
TCGA-ZH-A 8Y3	TCGA	WGS	*PBRM1*	3	52620649	52620656	TCACAGAC AAAAACA TCC	-	Deletion	D1055_E1060 del	Inframe deletion
TCGA-ZH-A 8Y3	TCGA	WGS	*IDH2*	15	90631838	90631838	C	T	SNV	R172K	Missense
TCGA-ZH-A 8Y2	TCGA	WGS	*CDKN2A*	9						Homo Del	Copy number loss
TCGA-ZH-A 8Y2	TCGA	WGS	*BAP1*	3	52442599	52442599	A	G	SNV	L49P	Missense
TCGA-ZH-A 8Y2	TCGA	WGS	*ATM*	11	108121693	108121693	C	A	SNV	Q501K	Missense
TCGA-ZH-A 8Y1	TCGA	WGS	*PIK3CA*	3	178936091	178936091	G	A	SNV	E545K	Missense
TCGA-ZH-A 8Y1	TCGA	WGS	*PBRM1*	3	52678790	52678790	T	A	SNV	K277*	Nonsense
TCGA-ZH-A 8Y1	TCGA	WGS	*KMT2C*	7	151945667	151945668	-	T	Insertion	Q618 fs	Frameshift
TCGA-ZH-A 8Y1	TCGA	WGS	*FGFR2*	10					Fusion	FGFR2-FRK	Fusion
TCGA-ZD-A 8I3	TCGA	WGS	*CDKN2A*	9	21971108	21971108	C	A	SNV	D84Y	Missense
TCGA-WD-A 7RX	TCGA	WGS	*ARID1A*	1	27101387	27101394	CCCTCTGC	-	Deletion	S1558 fs	Frameshift
TCGA-W6-A A0T	TCGA	WGS	*NF1*	17	29664894	29664894	C	T	SNV	Q2234*	Nonsense
TCGA-W6-A A0S	TCGA	WGS	*KMT2C*	7	151962254	151962254	C	T	SNV	G351E	Missense
TCGA-W6-A A0S	TCGA	WGS	*IDH2*	15	90631838	90631838	C	T	SNV	R172K	Missense
TCGA-W5-A A38	TCGA	WGS	*PBRM1*	3	52661362	52661362	T	A	SNV	R490*	Nonsense
TCGA-W5-A A34	TCGA	WGS	*IDH1*	2	209113113	209113113	G	A	SNV	R132C	Missense
TCGA-W5-A	TCGA	WGS	*FGFR2*	10	123279605	123279605	A	C	SNV	F276C	Missense

A34											
TCGA-W5-A A31	TCGA	WGS	*NRAS*	1	115256529	115256529	T	C	SNV	Q61R	Missense
TCGA-W5-A A31	TCGA	WGS	*BAP1*	3	52440917	52440917	C	T	SNV	p.W196*	Nonsense
TCGA-W5-A A30	TCGA	WGS	*PBRM1*	3	52651334	52651334	C	A	SNV	E588*	Nonsense
TCGA-W5-A A30	TCGA	WGS	*BRCA2*	13	32913032	32913032	G	C	SNV	E1514Q	Missense
TCGA-W5-A A2Z	TCGA	WGS	*FGFR2*	10					Fusion	FGFR2-CCDC 186	Fusion
TCGA-W5-A A2W	TCGA	WGS	*PBRM1*	3	52661362	52661362	T	A	SNV	R490*	Nonsense
TCGA-W5-A A2W	TCGA	WGS	*FGFR2*	10					Fusion	FGFR2-BICC1	Fusion
TCGA-W5-A A2Q	TCGA	WGS	*FGFR2*	10					Fusion	FGFR2-KIAA1 598	Fusion
TCGA-W5-A A2O	TCGA	WGS	*PBRM1*	3	52651334	52651334	C	A	SNV	E588*	Nonsense
TCGA-W5-A A2O	TCGA	WGS	*BRCA2*	13	32913032	32913032	G	C	SNV	E1514Q	Missense
TCGA-W5-A A2I	TCGA	WGS	*TP53*	17	7578509	7578509	A	G	SNV	C141R	Missense
TCGA-W5-A A2I	TCGA	WGS	*PBRM1*	3	52621438	52621439	-	AAT	Insertion	V1017 insL	Inframe Insertion
TCGA-W5-A A2I	TCGA	WGS	*EPHA2*	1	16451813	16451813	T	A	SNV	D943V	Missense
TCGA-W5-A A2G	TCGA	WGS	*IDH1*	2	209113113	209113113	G	A	SNV	R132C	Missense
TCGA-W5-A A2G	TCGA	WGS	*EPHA2*	1	16462176	16462176	T	A	SNV	K468*	Nonsense
TCGA-W5-A A2G	TCGA	WGS	*CDKN2A*	9						Homo Del	Copy number loss
TCGA-W5-A A2G	TCGA	WGS	*BAP1*	3	52437564	52437565	-	A	Insertion	G533 fs	Frameshift
TCGA-FV-A 3I0	TCGA	WGS	*PBRM1*	3	52584506	52584506	A	G	SNV	Y1610H	Missense
TCGA-FV-A 3I0	TCGA	WGS	*FGFR2*	10	123325033	123325033	G	A	SNV	P99S	Missense
TCGA-BC-A 10Q	TCGA	WGS	*IDH1*	2	209113113	209113113	G	A	SNV	R132C	Missense
TCGA-BC-A 10Q	TCGA	WGS	*BAP1*	3	52440346	52440349	CGGG	-	Deletion	P235 fs	Frameshift

TCGA-5A-A8ZG	TCGA	WGS	*TP53*	17	7577102	7577102	C	T	SNV	G279E	Missense
TCGA-5A-A8ZG	TCGA	WGS	*ARID2*	12	46246471	46246471	C	-	Deletion	T1522 fs	Frameshift
TCGA-5A-A8ZF	TCGA	WGS	*SMAD4*	18	48593445	48593462	TCAGGTGCCTTAGTGACC	-	Deletion	V399_H404 delinsD	Inframe deletion
TCGA-5A-A8ZF	TCGA	WGS	*PIK3CA*	3	178936091	178936091	G	A	SNV	E545K	Missense
TCGA-5A-A8ZF	TCGA	WGS	*KRAS*	12	25398284	25398284	C	T	SNV	G12D	Missense
TCGA-5A-A8ZF	TCGA	WGS	*ARID1A*	1	27105967	27105967	G	T	SNV	E1860*	Nonsense
TCGA-4G-AAZT	TCGA	WGS	*PIK3CA*	3	178952085	178952085	A	T	SNV	H1047L	Missense
TCGA-4G-AAZT	TCGA	WGS	*PBRM1*	3	52651496	52651496	G	A	SNV	R534*	Nonsense
TCGA-4G-AAZT	TCGA	WGS	*BAP1*	3	52439916	52439916	T	G	SNV	T266P	Missense
TCGA-4G-AAZT	TCGA	WGS	*ARID1A*	1	26772807	26772827	TGGAGCAGGAGCAATTCAGTT	-	Deletion	Splice site	Splice site
TCGA-4G-AAZO	TCGA	WGS	*BAP1*	3	52437801	52437802	-	T	Insertion	E454 fs	Frameshift
TCGA-4G-AAZO	TCGA	WGS	*ARID1A*	1	27107135	27107136	-	A	Insertion	E2250 fs	Frameshift
TCGA-4G-AAZG	TCGA	WGS	*FGFR2*	10	123239535	123239535	C	A	SNV	E768*	Nonsense
TCGA-4G-AAZG	TCGA	WGS	*BRAF*	7	140481411	140481411	C	G	SNV	G466A	Missense
TCGA-4G-AAZG	TCGA	WGS	*ARID1A*	1	27089711	27089711	C	-	Deletion	M890*	Nonsense
TCGA-4G-AAZF	TCGA	WGS	*BAP1*	3	52441235	52441235	G	A	SNV	R179W	Missense
TCGA-3X-AAVA	TCGA	WGS	*IDH2*	15	90631837	90631837	C	A	SNV	R172S	Missense
TCGA-3X-AAVA	TCGA	WGS	*EPHA2*	1	16459707	16459707	T	A	SNV	N674I	Missense
TCGA-3X-AAV9	TCGA	WGS	*CDKN2A*	9						Homo Del	Copy number loss
ICC24	Sia D *et al*	WES	*FGFR2*	10					Fusion	FGFR2-PPHLN1	Fusion
ICC12	Sia D *et al*	WES	*EPHA2*	1	16461605	16461605	T	C	SNV	Y503C	Missense

ICC12	Sia D *et al*	WES	*ARID1A*	1	27106426	27106426	A	T	SNV	K1630*	Nonsense
ICC12	Sia D *et al*	WES	*ARID1A*	1	27106432	27106432	A	T	SNV	I1632F	Missense
QX037	Sheng YY *et al*	WES	*BAP1*	3	52437276	52437276	G	A	SNV	Q590*	Nonsense
QX036	Sheng YY *et al*	WES	*EPHA2*	1	16477392	16477392	C	A	SNV	G51V	Missense
QX036	Sheng YY *et al*	WES	*ARID2*	12	46230376	46230376	G	A	SNV	W237*	Nonsense
QX035	Sheng YY *et al*	WES	*PIK3CA*	3	178936073	178936073	C	A	SNV	P539T	Missense
QX035	Sheng YY *et al*	WES	*PIK3CA*	3	178936095	178936095	A	G	SNV	Q546R	Missense
QX035	Sheng YY *et al*	WES	*PIK3CA*	3	178936083	178936083	A	G	SNV	E542G	Missense
QX034	Sheng YY *et al*	WES	*BAP1*	3	52441246	52441246	G	A	SNV	P175L	Missense
QX032	Sheng YY *et al*	WES	*KRAS*	12	25398284	25398284	C	T	SNV	G12D	Missense
QX032	Sheng YY *et al*	WES	*ATM*	11	108143523	108143523	A	T	SNV	Q1076H	Missense
QX029	Sheng YY *et al*	WES	*NF1*	17	29654857	29654857	G	A	SNV	R1849Q	Missense
QX024	Sheng YY *et al*	WES	*PBRM1*	3	52663002	52663002	C	T	SNV	D451N	Missense
QX024	Sheng YY *et al*	WES	*FGFR2*	10	123274794	123274794	T	C	SNV	Y263C	Missense
QX024	Sheng YY *et al*	WES	*ARID1A*	1	27099976	27099976	T	G	SNV	Y1285*	Nonsense
QX023	Sheng YY *et al*	WES	*TP53*	17	7577127	7577127	C	A	SNV	E271*	Nonsense
QX023	Sheng YY *et al*	WES	*SMAD4*	18	48584504	48584504	C	-	Deletion	A226 fs	Frameshift
QX022	Sheng YY *et al*	WES	*BAP1*	3	52437267	52437267	G	A	SNV	Q593*	Nonsense
QX020	Sheng YY *et al*	WES	*TP53*	17	7577539	7577539	G	A	SNV	R116W	Missense
QX020	Sheng YY *et al*	WES	*KRAS*	12	25398285	25398285	C	A	SNV	G12C	Missense
QX020	Sheng YY *et al*	WES	*ATM*	11	108124614	108124614	G	T	SNV	D658Y	Missense
QX019	Sheng YY *et al*	WES	*TP53*	17	7578535	7578535	T	G	SNV	K132T	Missense
QX018	Sheng YY *et al*	WES	*PBRM1*	3	52588830	52588830	T	C	SNV	M1420V	Missense
QX018	Sheng YY *et al*	WES	*EPHA2*	1	16458245	16458245	G	A	SNV	R816W	Missense
QX018	Sheng YY *et al*	WES	*CDKN2A*	9	21971028	21971028	C	T	SNV	W110*	Nonsense
QX018	Sheng YY *et al*	WES	*ARID1A*	1	27106732	27106732	C	T	SNV	Q2115*	Nonsense
QX015	Sheng YY *et al*	WES	*TP53*	17	7578271	7578271	T	C	SNV	H61R	Missense
QX015	Sheng YY *et al*	WES	*BRCA2*	13	32907455	32907455	A	G	SNV	I614V	Missense
QX015	Sheng YY *et al*	WES	*ARID2*	12	46244283	46244283	C	G	SNV	Q793E	Missense
QX013	Sheng YY *et al*	WES	*BAP1*	3	52437163	52437163	G	C	SNV	Y627*	Nonsense
QX013	Sheng YY *et al*	WES	*ATM*	11	108213964	108213964	C	T	SNV	Q2762*	Nonsense
QX012	Sheng YY *et al*	WES	*IDH1*	2	209113112	209113112	C	A	SNV	R132L	Missense
QX012	Sheng YY *et al*	WES	*FBXW7*	4	153259054	153259054	T	A	SNV	D136V	Missense
QX011	Sheng YY *et al*	WES	*TP53*	17	7573982	7573982	C	A	SNV	E217*	Nonsense
QX011	Sheng YY *et al*	WES	*KMT2D*	12	49424678	49424678	G	A	SNV	Q4557*	Nonsense

QX010	Sheng YY *et al*	WES	*IDH2*	15	90631839	90631839	T	A	SNV	R172W	Missense
QX010	Sheng YY *et al*	WES	*ATM*	11	108216575	108216575	C	A	SNV	P2842T	Missense
QX009	Sheng YY *et al*	WES	*IDH2*	15	90631838	90631838	C	T	SNV	R172K	Missense
QX009	Sheng YY *et al*	WES	*IDH2*	15	90631837	90631837	C	A	SNV	R172S	Missense
QX009	Sheng YY *et al*	WES	*EPHA2*	1	16458872	16458872	C	A	SNV	Splice site	Splice site
QX007	Sheng YY *et al*	WES	*PBRM1*	3	52621446	52621446	C	T	SNV	E984K	Missense
QX007	Sheng YY *et al*	WES	*IDH1*	2	209113113	209113113	G	A	SNV	R132C	Missense
QX005	Sheng YY *et al*	WES	*PBRM1*	3	52610716	52610716	T	C	SNV	Splice site	Splice site
QX005	Sheng YY *et al*	WES	*IDH1*	2	209113113	209113113	G	A	SNV	R132C	Missense
QX005	Sheng YY *et al*	WES	*FGFR2*	10	123274794	123274794	T	C	SNV	Y263C	Missense
QX005	Sheng YY *et al*	WES	*EPHA2*	1	16475508	16475524	GGCATGTC ATTCATGA T	-	Deletion	I58 fs	Frameshift
QX005	Sheng YY *et al*	WES	*BAP1*	3	52442606	52442606	T	A	SNV	I47F	Missense
QX004	Sheng YY *et al*	WES	*IDH2*	15	90631839	90631839	T	A	SNV	R172W	Missense
QX004	Sheng YY *et al*	WES	*EPHA2*	1	16460017	16460017	A	T	SNV	I608N	Missense
QX003	Sheng YY *et al*	WES	*IDH1*	2	209113113	209113113	G	C	SNV	R132G	Missense
QX003	Sheng YY *et al*	WES	*BAP1*	3	52439196	52439196	T	-	Deletion	N349 fs	Frameshift
QX001	Sheng YY *et al*	WES	*PBRM1*	3	52620470	52620470	G	A	SNV	R1088*	Nonsense
QX001	Sheng YY *et al*	WES	*KMT2D*	12	49434490	49434490	C	A	SNV	A2355S	Missense
QX001	Sheng YY *et al*	WES	*IDH1*	2	209113113	209113113	G	A	SNV	R132C	Missense
QX001	Sheng YY *et al*	WES	*ARID1A*	1	27092858	27092858	G	C	SNV	Splice site	Splice site
TP_2038	Robinson DR *et al*	WGS	*PIK3CA*	3	178952085	178952085	A	G	SNV	H1047R	Missense
TP_2038	Robinson DR *et al*	WGS	*EPHA2*	1	16464480	16464480	G	-	Deletion	R394 fs	Frameshift
TP_2038	Robinson DR *et al*	WGS	*BAP1*	3	52443622	52443622	C	A	SNV	V24F	Missense
TP_2038	Robinson DR *et al*	WGS	*ARID1A*	1	27102103	27102103	A	T	SNV	K1677*	Nonsense
TP_2008	Robinson DR *et al*	WGS	*CDKN2A*	9						Homo Del	Copy number loss
TP_2008	Robinson DR *et al*	WGS	*BRAF*	7	140477861	140477861	T	C	SNV	K483E	Missense
MO_1552	Robinson DR *et al*	WGS	*PBRM1*	3	52712520	52712520	G	A	SNV	R78*	Nonsense
MO_1552	Robinson DR *et al*	WGS	*IDH1*	2	209113112	209113112	C	A	SNV	R132L	Missense
MO_1552	Robinson DR	WGS	*FGFR2*	10	123274774	123274774	A	G	SNV	C382R	Missense

	et al										
MO_1550	Robinson DR *et al*	WGS	*KRAS*	12	25398285	25398285	C	A	SNV	G12C	Missense
MO_1550	Robinson DR *et al*	WGS	*IDH1*	2	209113113	209113113	G	A	SNV	R132C	Missense
MO_1550	Robinson DR *et al*	WGS	*CDKN2A*	9						Homo Del	Copy number loss
MO_1448	Robinson DR *et al*	WGS	*FGFR2*	10	123310908	123310928	GTTGGCCG CAGGCAC AGCATG	-	Deletion	H167 del	Inframe deletion
MO_1402	Robinson DR *et al*	WGS	*IDH1*	2	209113113	209113113	G	A	SNV	R132C	Missense
MO_1402	Robinson DR *et al*	WGS	*ARID1A*	1						Homo Del	Copy number loss
MO_1369	Robinson DR *et al*	WGS	*TP53*	17	7577538	7577538	C	T	SNV	R248Q	Missense
MO_1369	Robinson DR *et al*	WGS	*IDH1*	2	209113113	209113113	G	A	SNV	R132C	Missense
MO_1338	Robinson DR *et al*	WGS	*TP53*	17	7579394	7579394	G	-	Deletion	P98 fs	Frameshift
MO_1338	Robinson DR *et al*	WGS	*SMAD4*	18						Homo Del	Copy number loss
MO_1203	Robinson DR *et al*	WGS	*PBRM1*	3	52621445	52621446	TC	-	Deletion	E991 fs	Frameshift
MO_1203	Robinson DR *et al*	WGS	*FGFR2*	10					Fusion	FGFR2-KIAA1 967	Fusion
MO_1203	Robinson DR *et al*	WGS	*BAP1*	3	52441252	52441252	T	C	SNV	Y173C	Missense
MO_1147	Robinson DR *et al*	WGS	*NRAS*	1	115256529	115256529	T	C	SNV	Q61R	Missense
MO_1147	Robinson DR *et al*	WGS	*ARID1A*	1	27023559	27023560	-	C	Insertion	P225 fs	Frameshift
MO_1039	Robinson DR *et al*	WGS	*TP53*	17	7577139	7577139	G	A	SNV	R267W	Missense
MO_1039	Robinson DR *et al*	WGS	*FGFR2*	10					Fusion	FGFR2-BICC1	Fusion
MO_1039	Robinson DR *et al*	WGS	*ATM*	11	108122687	108122687	A	-	Deletion	K578 fs	Frameshift
LEC148 (11)	Nepal C *et al*	WES	*TP53*	17	7577121	7577121	G	A	SNV	R273C	Missense
LEC148 (11)	Nepal C *et al*	WES	*NRAS*	1	115258747	115258747	C	A	SNV	G12V	Missense
LEC148 (11)	Nepal C *et al*	WES	*EPHA2*	1	16474976	164749767	-	C	Insertion	E241*	Nonsense
LEC143(8)	Nepal C *et al*	WES	*KRAS*	12	25398281	25398281	C	T	SNV	G13D	Missense

LEC143(8)	Nepal C *et al*	WES	*FGFR2*	10	123263395	123263395	G	A	SNV	R451C	Missense
15	Nepal C *et al*	WES	*TGFBR2*	3	30732969	30732969	C	T	SNV	R553C	Missense
15	Nepal C *et al*	WES	*FBXW7*	4	153245377	153245377	A	T	SNV	I605N	Missense
15	Nepal C *et al*	WES	*BRAF*	7	140481411	140481411	C	A	SNV	G466V	Missense
15	Nepal C *et al*	WES	*ARID1A*	1	27105756	27105757	-	T	Insertion	S1791 fs	Frameshift
13	Nepal C *et al*	WES	*PTEN*	10	89720833	89720833	A	-	Deletion	N329 fs	Frameshift
13	Nepal C *et al*	WES	*IDH2*	15	90631837	90631837	C	A	SNV	R172S	Missense
13	Nepal C *et al*	WES	*ARID1A*	1	27057919	27057919	C	A	SNV	Q543K	Missense
12	Nepal C *et al*	WES	*ARID1A*	1	27057937	27057937	C	-	Deletion	Y551 fs	Frameshift
10	Nepal C *et al*	WES	*KRAS*	12	25398284	25398284	C	T	SNV	G12D	Missense
9	Nepal C *et al*	WES	*KRAS*	12	25398284	25398284	C	T	SNV	G12D	Missense
7	Nepal C *et al*	WES	*KRAS*	12	25398284	25398284	C	T	SNV	G12D	Missense
6	Nepal C *et al*	WES	*KRAS*	12	25398284	25398284	C	T	SNV	G12D	Missense
5	Nepal C *et al*	WES	*IDH1*	2	209113113	209113113	G	A	SNV	R132C	Missense
5	Nepal C *et al*	WES	*EPHA2*	1	16462176	16462176	T	A	SNV	K468*	Nonsense
5	Nepal C *et al*	WES	*BAP1*	3	52437565	52437565	A	-	Deletion	F532 fs	Frameshift
4	Nepal C *et al*	WES	*IDH1*	2	209113113	209113113	G	C	SNV	R132G	Missense
3	Nepal C *et al*	WES	*IDH1*	2	209113113	209113113	G	A	SNV	R132C	Missense
3	Nepal C *et al*	WES	*BAP1*	3	52439921	524399212	-	T	Insertion	R264 fs	Frameshift
3	Nepal C *et al*	WES	*ARID1A*	1	27106829	27106830	-	G	Insertion	Y2148 fs	Frameshift
2	Nepal C *et al*	WES	*PIK3CA*	3	178952085	178952085	A	G	SNV	H1047R	Missense
2	Nepal C *et al*	WES	*IDH1*	2	209113113	209113113	G	C	SNV	R132G	Missense
2	Nepal C *et al*	WES	*BAP1*	3	52441470	52441470	C	G	SNV	G128R	Missense
1	Nepal C *et al*	WES	*IDH1*	2	209113113	209113113	G	C	SNV	R132G	Missense
1	Nepal C *et al*	WES	*BAP1*	3	52441470	52441470	C	G	SNV	G128R	Missense
BD95	Nakamura H *et al*	WES	*NRAS*	1	115256529	115256529	T	C	SNV	Q61R	Missense
BD92	Nakamura H *et al*	WES	*EPHA2*	1	16462161	16462161	A	-	Deletion	Y473 fs	Frameshift
BD92	Nakamura H *et al*	WES	*BAP1*	3	52436631	52436632	-	A	Insertion	S680 fs	Frameshift
BD88	Nakamura H *et al*	WES	*TP53*	17	7576927	7576927	C	A	SNV	Splice site	Splice site
BD88	Nakamura H *et al*	WES	*TP53*	17	7576926	7576926	G	A	SNV	A307V	Missense
BD88	Nakamura H *et al*	WES	*KRAS*	12	25398284	25398284	C	T	SNV	G12D	Missense
BD86	Nakamura H *et*	WES	*TP53*	17	7576855	7576855	G	A	SNV	Q331*	Nonsense

	al										
BD86	Nakamura H *et al*	WES	*KRAS*	12	25398284	25398284	C	A	SNV	G12V	Missense
BD86	Nakamura H *et al*	WES	*FBXW7*	4	153249510	153249510	C	A	SNV	G423V	Missense
BD84	Nakamura H *et al*	WES	*NRAS*	1	115256529	115256529	T	C	SNV	Q61R	Missense
BD84	Nakamura H *et al*	WES	*EPHA2*	1	16464404	16464404	G	C	SNV	S419*	Nonsense
BD84	Nakamura H *et al*	WES	*BRAF*	7	140453146	140453146	G	C	SNV	L597V	Missense
BD82	Nakamura H *et al*	WES	*SMAD4*	18	48603120	48603120	C	A	SNV	S474*	Nonsense
BD81	Nakamura H *et al*	WES	*IDH1*	2	209113112	209113112	C	A	SNV	R132L	Missense
BD81	Nakamura H *et al*	WES	*ARID1A*	1	27101711	27101711	G	T	SNV	G1665*	Nonsense
BD8	Nakamura H *et al*	WES	*IDH1*	2	209113113	209113113	G	A	SNV	R132C	Missense
BD8	Nakamura H *et al*	WES	*BAP1*	3	52437557	52437557	T	-	Deletion	N435 fs	Frameshift
BD79	Nakamura H *et al*	WES	*FGFR2*	10					Fusion	FGFR2-BICC1	Fusion
BD79	Nakamura H *et al*	WES	*BAP1*	3	52441226	52441226	C	A	SNV	E182*	Nonsense
BD78	Nakamura H *et al*	WES	*ARID2*	12	46243920	46243920	C	T	SNV	Q672*	Nonsense
BD74	Nakamura H *et al*	WES	*TP53*	17	7578529	7578529	A	C	SNV	F134C	Missense
BD74	Nakamura H *et al*	WES	*KRAS*	12	25398284	25398284	C	G	SNV	G12A	Missense
BD69	Nakamura H *et al*	WES	*ARID1A*	1	27106106	27106106	G	-	Deletion	R1906 fs	Frameshift
BD66	Nakamura H *et al*	WES	*KRAS*	12	25398284	25398284	C	T	SNV	G12D	Missense
BD58	Nakamura H *et al*	WES	*SMAD4*	18	48604788	48604788	A	G	SNV	D537G	Missense
BD58	Nakamura H *et al*	WES	*EPHA2*	1	16464358	16464358	G	-	Deletion	N435 fs	Frameshift
BD58	Nakamura H *et al*	WES	*ARID1A*	1	27105541	27105541	G	T	SNV	E1718*	Nonsense
BD54	Nakamura H *et al*	WES	*EPHA2*	1	16461569	16461569	T	A	SNV	Q515L	Missense

BD50	Nakamura H *et al*	WES	*BAP1*	3	52437677	52437678	-	T	Insertion	D494 fs	Frameshift
BD5	Nakamura H *et al*	WES	*PIK3CA*	3	178936082	178936082	G	A	SNV	E542K	Missense
BD5	Nakamura H *et al*	WES	*KRAS*	12	25398284	25398284	C	T	SNV	G12D	Missense
BD47	Nakamura H *et al*	WES	*KRAS*	12	25380275	25380275	T	G	SNV	Q61H	Missense
BD47	Nakamura H *et al*	WES	*APC*	5	112175729	112175729	C	T	SNV	Q1480*	Nonsense
BD41	Nakamura H *et al*	WES	*ARID2*	12	46244946	46244946	C	T	SNV	Q1014*	Nonsense
BD38	Nakamura H *et al*	WES	*TP53*	17	7578369	7578369	A	G	SNV	Splice site	Splice site
BD38	Nakamura H *et al*	WES	*PIK3CA*	3	178941873	178941873	A	G	SNV	Q731R	Missense
BD38	Nakamura H *et al*	WES	*NRAS*	1	115258747	115258747	C	T	SNV	G12D	Missense
BD33	Nakamura H *et al*	WES	*PBRM1*	3	52661289	52661290	-	T	Insertion	S514 fs	Frameshift
BD33	Nakamura H *et al*	WES	*FGFR2*	10					Fusion	FGFR2-BICC1	Fusion
BD33	Nakamura H *et al*	WES	*EPHA2*	1	16474965	16474966	-	G	Insertion	R244 fs	Frameshift
BD33	Nakamura H *et al*	WES	*BAP1*	3	52441333	52441333	C	A	SNV	Splice site	Splice site
BD33	Nakamura H *et al*	WES	*ARID1A*	1	27059264	27059264	C	G	SNV	S634*	Nonsense
BD31	Nakamura H *et al*	WES	*PBRM1*	3	52675988	52675988	C	A	SNV	E357*	Nonsense
BD30	Nakamura H *et al*	WES	*KRAS*	12	25398284	25398284	C	A	SNV	G12V	Missense
BD29	Nakamura H *et al*	WES	*TP53*	17	7578265	7578265	A	G	SNV	I195T	Missense
BD29	Nakamura H *et al*	WES	*PIK3CA*	3	178936091	178936091	G	A	SNV	E545K	Missense
BD29	Nakamura H *et al*	WES	*KRAS*	12	25398284	25398284	C	T	SNV	G12D	Missense
BD28	Nakamura H *et al*	WES	*NRAS*	1	115258747	115258747	C	T	SNV	G12D	Missense
BD28	Nakamura H *et al*	WES	*BAP1*	3	52441252	52441252	T	C	SNV	Y173C	Missense
BD25	Nakamura H *et al*	WES	*TP53*	17	7578406	7578406	C	T	SNV	R175H	Missense

BD25	Nakamura H *et al*	WES	*KRAS*	12	25398284	25398284	C	A	SNV	G12V	Missense
BD247	Nakamura H *et al*	WES	*KRAS*	12	25398284	25398284	C	T	SNV	G12D	Missense
BD245	Nakamura H *et al*	WES	*PBRM1*	3	52643726	52643727	-	A	Insertion	M724 fs	Frameshift
BD245	Nakamura H *et al*	WES	*IDH1*	2	209113113	209113113	G	C	SNV	R132G	Missense
BD244	Nakamura H *et al*	WES	*TP53*	17	7577094	7577094	G	A	SNV	R282W	Missense
BD243	Nakamura H *et al*	WES	*TP53*	17	7578234	7578240	ATACTCC	-	Deletion	E204 fs	Frameshift
BD243	Nakamura H *et al*	WES	*KRAS*	12	25398284	25398284	C	T	SNV	G12D	Missense
BD239	Nakamura H *et al*	WES	*TGFBR2*	3	30729874	30729874	A	G	SNV	Splice site	Splice site
BD239	Nakamura H *et al*	WES	*PTEN*	10	89717708	89717708	C	T	SNV	Q245*	Nonsense
BD239	Nakamura H *et al*	WES	*BRAF*	7	140453193	140453193	T	C	SNV	N581S	Missense
BD237	Nakamura H *et al*	WES	*ARID1A*	1	27102084	27102084	G	A	SNV	W1670*	Nonsense
BD234	Nakamura H *et al*	WES	*ARID2*	12	46246372	46246372	C	G	SNV	S1489*	Nonsense
BD234	Nakamura H *et al*	WES	*ARID1A*	1	27106648	27106648	G	A	SNV	G2087R	Missense
BD233	Nakamura H *et al*	WES	*KRAS*	12	25378562	25378562	C	T	SNV	A146T	Missense
BD231	Nakamura H *et al*	WES	*TP53*	17	7578271	7578271	T	C	SNV	H193R	Missense
BD230	Nakamura H *et al*	WES	*SMAD4*	18	48604802	48604803	-	C	Insertion	M543 fs	Frameshift
BD230	Nakamura H *et al*	WES	*KRAS*	12	25398284	25398284	C	T	SNV	G12D	Missense
BD23	Nakamura H *et al*	WES	*TP53*	17	7578190	7578190	T	C	SNV	Y220C	Missense
BD23	Nakamura H *et al*	WES	*FGFR2*	10					Fusion	FGFR2-KCTD1	Fusion
BD229	Nakamura H *et al*	WES	*TP53*	17	7577539	7577539	G	C	SNV	R248G	Missense
BD229	Nakamura H *et al*	WES	*KRAS*	12	25398285	25398285	C	A	SNV	G12C	Missense
BD228	Nakamura H *et al*	WES	*NF1*	17	29667664	29667664	G	T	SNV	Splice site	Splice site

BD227	Nakamura H *et al*	WES	*TP53*	17	7578265	7578265	A	T	SNV	I195N	Missense
BD226	Nakamura H *et al*	WES	*BAP1*	3	52441312	52441312	G	-	Deletion	P153 fs	Frameshift
BD225	Nakamura H *et al*	WES	*BRCA2*	13	32912159	32912159	C	A	SNV	H1223N	Missense
BD224	Nakamura H *et al*	WES	*PIK3CA*	3	178921553	178921553	T	A	SNV	N345K	Missense
BD223	Nakamura H *et al*	WES	*SMAD4*	18	48593557	48593557	G	T	SNV	K436N	Missense
BD223	Nakamura H *et al*	WES	*KMT2D*	12	49418460	49418460	A	G	SNV	L5318S	Missense
BD223	Nakamura H *et al*	WES	*ATM*	11	108155085	108155085	A	G	SNV	N1293S	Missense
BD222	Nakamura H *et al*	WES	*PIK3CA*	3	178952085	178952085	A	G	SNV	H1047R	Missense
BD222	Nakamura H *et al*	WES	*EPHA2*	1	16475279	16475279	G	-	Deletion	F139 fs	Frameshift
BD221	Nakamura H *et al*	WES	*KRAS*	12	25398284	25398284	C	A	SNV	G12V	Missense
BD221	Nakamura H *et al*	WES	*FBXW7*	4	153247204	153247204	C	A	SNV	C533F	Missense
BD220	Nakamura H *et al*	WES	*TP53*	17	7578550	7578550	G	A	SNV	S127F	Missense
BD219	Nakamura H *et al*	WES	*IDH2*	15	90631838	90631838	C	T	SNV	R172K	Missense
BD218	Nakamura H *et al*	WES	*NRAS*	1	115256529	115256529	T	C	SNV	Q61R	Missense
BD217	Nakamura H *et al*	WES	*CDKN2A*	9	21971096	21971096	C	A	SNV	E88*	Nonsense
BD216(BD195)	Nakamura H *et al*	WES	*KRAS*	12	25398284	25398284	C	T	SNV	G12D	Missense
BD215(BD196)	Nakamura H *et al*	WES	*SMAD4*	18	48591903	48591903	C	T	SNV	P356S	Missense
BD215(BD196)	Nakamura H *et al*	WES	*KRAS*	12	25380275	25380275	T	G	SNV	Q61H	Missense
BD214	Nakamura H *et al*	WES	*TP53*	17	7577557	7577557	A	C	SNV	C242G	Missense
BD214	Nakamura H *et al*	WES	*FGFR2*	10	123258035	123258035	T	C	SNV	N549S	Missense
BD214	Nakamura H *et al*	WES	*BRAF*	7	140453193	140453193	T	C	SNV	N581S	Missense
BD213	Nakamura H *et al*	WES	*TP53*	17	7577106	7577106	G	T	SNV	P278T	Missense

BD213	Nakamura H *et al*	WES	*KRAS*	12	25398284	25398284	C	T	SNV	G12D	Missense
BD213	Nakamura H *et al*	WES	*EPHA2*	1	16455977	16455977	A	T	SNV	M926K	Missense
BD212	Nakamura H *et al*	WES	*IDH1*	2	209113113	209113113	G	A	SNV	R132C	Missense
BD212	Nakamura H *et al*	WES	*APC*	5	112177484	112177484	C	T	SNV	P2065S	Missense
BD211	Nakamura H *et al*	WES	*TP53*	17	7578191	7578191	A	T	SNV	Y220N	Missense
BD211	Nakamura H *et al*	WES	*PBRM1*	3	52598156	52598156	T	C	SNV	N1237S	Missense
BD211	Nakamura H *et al*	WES	*KRAS*	12	25398284	25398284	C	T	SNV	G12D	Missense
BD211	Nakamura H *et al*	WES	*CDKN2A*	9	21974689	21974690	CC	-	Deletion	R46 fs	Frameshift
BD210	Nakamura H *et al*	WES	*SMAD4*	18	48591918	48591918	C	T	SNV	R361C	Missense
BD21	Nakamura H *et al*	WES	*PIK3CA*	3	178952085	178952085	A	T	SNV	H1047L	Missense
BD21	Nakamura H *et al*	WES	*FGFR2*	10	123274794	123274794	T	C	SNV	Y376C	Missense
BD21	Nakamura H *et al*	WES	*BAP1*	3	52439913	52439913	G	A	SNV	Q267*	Nonsense
BD203	Nakamura H *et al*	WES	*TP53*	17	7577534	7577534	C	A	SNV	R249S	Missense
BD200	Nakamura H *et al*	WES	*IDH1*	2	209113113	209113113	G	A	SNV	R132C	Missense
BD199	Nakamura H *et al*	WES	*BRCA2*	13	32913837	32913838	-	A	Insertion	N1784 fs	Frameshift
BD199	Nakamura H *et al*	WES	*BAP1*	3	52441448	52441448	G	-	Deletion	P135 fs	Frameshift
BD197	Nakamura H *et al*	WES	*FGFR2*	10					Fusion	FGFR2-BICC1	Fusion
BD197	Nakamura H *et al*	WES	*BAP1*	3	52441990	52441990	T	-	Deletion	K120 fs	Frameshift
BD19	Nakamura H *et al*	WES	*FGFR2*	10					Fusion	FGFR2-TXLNA	Fusion
BD18	Nakamura H *et al*	WES	*TP53*	17	7577022	7577022	G	A	SNV	R306*	Nonsense
BD18	Nakamura H *et al*	WES	*KRAS*	12	25398284	25398284	C	T	SNV	G12D	Missense
BD169	Nakamura H *et al*	WES	*PIK3CA*	3	178952074	178952074	G	T	SNV	M1043I	Missense

BD169	Nakamura H *et al*	WES	*IDH1*	2	209113113	209113113	G	C	SNV	R132G	Missense
BD169	Nakamura H *et al*	WES	*BRAF*	7	140501350	140501350	G	A	SNV	T241M	Missense
BD169	Nakamura H *et al*	WES	*BAP1*	3	52442512	52442512	T	-	Deletion	N78 fs	Frameshift
BD169	Nakamura H *et al*	WES	*ARID1A*	1	27089727	27089727	C	T	SNV	Q895*	Nonsense
BD167	Nakamura H *et al*	WES	*PBRM1*	3	52668670	52668671	-	T	Insertion	Y417 fs	Frameshift
BD167	Nakamura H *et al*	WES	*IDH1*	2	209113113	209113113	G	A	SNV	R132C	Missense
BD167	Nakamura H *et al*	WES	*ARID1A*	1	27105978	27105979	AG	-	Deletion	E1864 fs	Frameshift
BD157	Nakamura H *et al*	WES	*SMAD4*	18	48591918	48591918	C	G	SNV	R361G	Missense
BD157	Nakamura H *et al*	WES	*KRAS*	12	25398284	25398284	C	T	SNV	G12D	Missense
BD156	Nakamura H *et al*	WES	*TGFBR2*	3	30691929	30691929	A	G	SNV	N144S	Missense
BD154	Nakamura H *et al*	WES	*TP53*	17	7576852	7576852	C	A	SNV	Splice site	Splice site
BD154	Nakamura H *et al*	WES	*NF1*	17	29560168	29560168	G	T	SNV	M1215I	Missense
BD154	Nakamura H *et al*	WES	*KRAS*	12	25398284	25398284	C	A	SNV	G12V	Missense
BD154	Nakamura H *et al*	WES	*ARID2*	12	46287497	46287505	GGTCGCAGG	-	Deletion	Splice site	Splice site
BD154	Nakamura H *et al*	WES	*ARID2*	12	46243890	46243891	-	TT	Insertion	T663 fs	Frameshift
BD154	Nakamura H *et al*	WES	*ARID1A*	1	27092809	27092809	C	T	SNV	Q944*	Nonsense
BD153	Nakamura H *et al*	WES	*KRAS*	12	25398284	25398284	C	T	SNV	G12D	Missense
BD152	Nakamura H *et al*	WES	*SMAD4*	18	48591855	48591865	AAGGTTCCTTC	-	Deletion	V341 fs	Frameshift
BD152	Nakamura H *et al*	WES	*BRCA2*	13	32969041	32969042	-	C	Insertion	F3159 fs	Frameshift
BD152	Nakamura H *et al*	WES	*ATM*	11	108100026	108100026	T	G	SNV	Y103D	Missense
BD152	Nakamura H *et al*	WES	*ATM*	11	108199928	108199928	G	T	SNV	V2424L	Missense
BD15	Nakamura H *et al*	WES	*NRAS*	1	115256530	115256530	G	T	SNV	Q61K	Missense

BD149	Nakamura H *et al*	WES	*IDH2*	15	90631838	90631838	C	T	SNV	R172K	Missense
BD148	Nakamura H *et al*	WES	*KRAS*	12	25398284	25398284	C	A	SNV	G12V	Missense
BD148	Nakamura H *et al*	WES	*KMT2D*	12	49426862	49426862	G	C	SNV	L3876V	Missense
BD148	Nakamura H *et al*	WES	*ARID1A*	1	27057916	27057916	C	T	SNV	Q542*	Nonsense
BD147	Nakamura H *et al*	WES	*PBRM1*	3	52696293	52696293	C	T	SNV	Splice site	Splice site
BD146	Nakamura H *et al*	WES	*KMT2D*	12	49420661	49420661	G	A	SNV	R5030C	Missense
BD143	Nakamura H *et al*	WES	*TP53*	17	7578550	7578550	G	A	SNV	S127F	Missense
BD143	Nakamura H *et al*	WES	*KRAS*	12	25380275	25380275	T	G	SNV	Q61H	Missense
BD141	Nakamura H *et al*	WES	*IDH1*	2	209113112	209113112	C	A	SNV	R132L	Missense
BD141	Nakamura H *et al*	WES	*BAP1*	3	52443599	52443601	CTC	-	Deletion	E31 del	Inframe deletion
BD141	Nakamura H *et al*	WES	*ARID1A*	1	27101048	27101048	G	T	SNV	E1444*	Nonsense
BD140	Nakamura H *et al*	WES	*TP53*	17	7578517	7578517	G	A	SNV	A138V	Missense
BD14	Nakamura H *et al*	WES	*KMT2C*	7	151873888	151873888	G	A	SNV	R2884*	Nonsense
BD14	Nakamura H *et al*	WES	*ATM*	11	108188098	108188098	A	C	SNV	Splice site	Splice site
BD14	Nakamura H *et al*	WES	*ATM*	11	108202248	108202248	G	A	SNV	M2531I	Missense
BD138	Nakamura H *et al*	WES	*ARID2*	12	46254641	46254641	C	T	SNV	Q1611*	Nonsense
BD137	Nakamura H *et al*	WES	*NF1*	17	29667611	29667611	T	C	SNV	L2337P	Missense
BD134	Nakamura H *et al*	WES	*TP53*	17	7577538	7577538	C	A	SNV	R248L	Missense
BD134	Nakamura H *et al*	WES	*NRAS*	1	115256530	115256530	G	T	SNV	Q61K	Missense
BD132	Nakamura H *et al*	WES	*NF1*	17	29509684	29509684	G	A	SNV	Splice site	Splice site
BD130	Nakamura H *et al*	WES	*TP53*	17	7578212	7578212	G	A	SNV	R213*	Nonsense
BD130	Nakamura H *et al*	WES	*PIK3CA*	3	178916863	178916863	G	A	SNV	D84N	Missense

BD130	Nakamura H *et al*	WES	*PIK3CA*	3	178936082	178936082	G	A	SNV	E542K	Missense
BD130	Nakamura H *et al*	WES	*ARID1A*	1	27099947	27099947	C	T	SNV	R1276*	Nonsense
BD129	Nakamura H *et al*	WES	*TP53*	17	7577580	7577580	T	C	SNV	Y234C	missense
BD129	Nakamura H *et al*	WES	*SMAD4*	18	48575202	48575202	C	G	SNV	H132Q	Missense
BD129	Nakamura H *et al*	WES	*KRAS*	12	25398281	25398281	C	T	SNV	G13D	Missense
BD127	Nakamura H *et al*	WES	*KRAS*	12	25398284	25398284	C	T	SNV	G12D	Missense
BD127	Nakamura H *et al*	WES	*KMT2D*	12	49445217	49445217	G	A	SNV	P750L	Missense
BD120	Nakamura H *et al*	WES	*TP53*	17	7577538	7577538	C	T	SNV	R248Q	Missense
BD120	Nakamura H *et al*	WES	*ARID1A*	1	27087410	27087417	AGCACATC	-	Deletion	S662 fs	Frameshift
BD12	Nakamura H *et al*	WES	*PBRM1*	3	52595944	52595944	C	-	Deletion	G1376 fs	Frameshift
BD12	Nakamura H *et al*	WES	*NF1*	17	29654748	29654748	T	C	SNV	S1834P	Missense
BD118	Nakamura H *et al*	WES	*KRAS*	12	25380276	25380276	T	C	SNV	Q61R	Missense
BD117	Nakamura H *et al*	WES	*IDH2*	15	90631838	90631838	C	T	SNV	R172K	Missense
BD117	Nakamura H *et al*	WES	*EPHA2*	1	16458620	16458620	A	G	SNV	V755A	Missense
BD115	Nakamura H *et al*	WES	*IDH1*	2	209113113	209113113	G	C	SNV	R132G	Missense
BD115	Nakamura H *et al*	WES	*ATM*	11	108121448	108121448	T	A	SNV	L419*	Nonsense
BD111	Nakamura H *et al*	WES	*TP53*	17	7577085	7577085	C	T	SNV	E285K	Missense
BD111	Nakamura H *et al*	WES	*BRAF*	7	140481397	140481397	C	T	SNV	V471I	Missense
BD109	Nakamura H *et al*	WES	*ARID1A*	1	27099285	27099315	GTTTTCCT CACTCTGG AGCAGGA GCAATTCA	-	Deletion	Splice site	Splice site
BD105	Nakamura H *et al*	WES	*BAP1*	3	52443569	52443569	C	-	Deletion	Splice site	Splice site
BD104	Nakamura H *et al*	WES	*KRAS*	12	25398284	25398284	C	T	SNV	G12D	Missense

BD104	Nakamura H *et al*	WES	*ARID1A*	1	27094291	27094292	-	T	Insertion	S1001 fs	Frameshift
BD10	Nakamura H *et al*	WES	*SMAD4*	18	48575198	48575198	A	C	SNV	Y131S	Missense
BD10	Nakamura H *et al*	WES	*KRAS*	12	25398284	25398284	C	T	SNV	G12D	Missense
BD10	Nakamura H *et al*	WES	*KMT2D*	12	49427309	49427309	G	A	SNV	R3727C	Missense
CHCOSK003	Mimaki S *et al*	WES	*SMAD4*	18	48604701	48604701	G	A	SNV	G508D	Missense
CHCOSK003	Mimaki S *et al*	WES	*PIK3CA*	3	178937819	178937819	A	T	SNV	H665L	Missense
CHCOSK003	Mimaki S *et al*	WES	*ATM*	11	108180923	108180923	G	A	SNV	W1933*	Nonsense
CHCLO004	Mimaki S *et al*	WES	*TP53*	17	7577559	7577559	G	T	SNV	S241Y	Missense
CHCLO004	Mimaki S *et al*	WES	*SMAD4*	18	48591889	48591889	A	T	SNV	D351V	Missense
CHCLO004	Mimaki S *et al*	WES	*KRAS*	12	25378561	25378561	G	A	SNV	A146V	Missense
CHCLO004	Mimaki S *et al*	WES	*ARID2*	12	46231415	46231415	C	T	SNV	L419F	Missense
CHCLO003	Mimaki S *et al*	WES	*TGFBR2*	3	30691871	30691872	-	A	Insertion	P129 fs	Frameshift
CHCLO003	Mimaki S *et al*	WES	*BAP1*	3	52437557	52437557	T	-	Deletion	D535 fs	Frameshift
CHCLO003	Mimaki S *et al*	WES	*ARID1A*	1	27106954	27106954	A	T	SNV	K2189*	Nonsense
CHCLO002	Mimaki S *et al*	WES	*TP53*	17	7578212	7578212	G	A	SNV	R213*	Nonsense
CHCLO002	Mimaki S *et al*	WES	*SMAD4*	18	48575121	48575122	-	A	Insertion	N107 fs	Frameshift
CHCLO002	Mimaki S *et al*	WES	*KRAS*	12	25398284	25398284	C	A	SNV	G12V	Missense
CHCLO001	Mimaki S *et al*	WES	*KRAS*	12	25398284	25398284	C	T	SNV	G12D	Missense
CHCLO001	Mimaki S *et al*	WES	*FGFR2*	10	123274794	123274794	T	C	SNV	Y376C	Missense
ICC6	Kim YH *et al*	WES	*KRAS*	12	25398281	25398281	C	T	SNV	G13D	Missense
ICC31	Kim YH *et al*	WES	*KRAS*	12	25380276	25380276	T	A	SNV	Q61L	Missense
ICC31	Kim YH *et al*	WES	*KMT2C*	7	151945042	151945042	C	T	SNV	G826D	Missense
ICC31	Kim YH *et al*	WES	*APC*	5	112175639	112175639	C	T	SNV	R1450*	Nonsense
ICC30	Kim YH *et al*	WES	*TGFBR2*	3	30691938	30691938	T	A	SNV	I147N	Missense
ICC30	Kim YH *et al*	WES	*TGFBR2*	3	30713543	30713545	AGA	-	Deletion	E168 del	Inframe deletion
ICC30	Kim YH *et al*	WES	*APC*	5	112175951	112175951	G	T	SNV	E1554*	Nonsense
ICC30	Kim YH *et al*	WES	*APC*	5	112174759	112174760	AG	-	Deletion	R1158 fs	Frameshift
ICC29	Kim YH *et al*	WES	*TP53*	17	7578265 7	7578265 7	A	G	SNV	I195T	Missense
ICC26	Kim YH *et al*	WES	*KRAS*	12	25398284	25398284	C	T	SNV	G12D	Missense
ICC25	Kim YH *et al*	WES	*IDH1*	2	209113112	209113112	C	A	SNV	R132L	Missense
ICC25	Kim YH *et al*	WES	*BAP1*	3	52443621	52443621	A	T	SNV	V24D	Missense
ICC19	Kim YH *et al*	WES	*NF1*	17	29552120	29552120	A	G	SNV	D618G	Missense

ICC16	Kim YH *et al*	WES	*TP53*	17	7578263	7578263	G	A	SNV	R196*	Nonsense
ICC10	Kim YH *et al*	WES	*TP53*	17	7578440	7578440	T	C	SNV	K164E	Missense
ICC10	Kim YH *et al*	WES	*CDKN2A*	9	21974726	21974744	CCT CCAG CAGC GCCC GCA	-	Deletion	E27 del	Inframe deletion
CCA_TH_9	Jusakul A *et al*	WGS	*TP53*	17	7577593	7577594	-	A	Insertion	C229 fs	Frameshift
CCA_TH_9	Jusakul A *et al*	WGS	*SMAD4*	18	48593532	48593532	A	C	SNV	K428T	Missense
CCA_TH_8	Jusakul A *et al*	WGS	*TP53*	17	7578527	7578527	A	G	SNV	C135R	Missense
CCA_TH_6	Jusakul A *et al*	WGS	*FGFR2*	10	123298156	123298158	ACT	-	Deletion	V233 del	Inframe deletion
CCA_TH_22	Jusakul A *et al*	WGS	*EPHA2*	1	16475418	16475419	-	GCTCA	Insertion	E92 fs	Frameshift
CCA_TH_21	Jusakul A *et al*	WGS	*APC*	5	112176063	112176064	-	A	Insertion	P1594 fs	Frameshift
CCA_TH_20	Jusakul A *et al*	WGS	*EPHA2*	1	16482365	16482371	GGCCGCG	-	Deletion	A20 fs	Frameshift
CCA_TH_20	Jusakul A *et al*	WGS	*ARID1A*	1	27059183	27059183	C	A	SNV	S607*	Nonsense
CCA_TH_18	Jusakul A *et al*	WGS	*TP53*	17	7578513	7578514	-	T	Insertion	K139 fs	Frameshift
CCA_TH_18	Jusakul A *et al*	WGS	*TP53*	17	7578519	7578525	CAGTTGG	-	Deletion	F134 fs	Frameshift
CCA_TH_18	Jusakul A *et al*	WGS	*KMT2C*	7	151851222	151851222	G	C	SNV	S4050*	Nonsense
CCA_TH_18	Jusakul A *et al*	WGS	*FBXW7*	4	153303421	153303422	-	T	Insertion	K22 fs	Frameshift
CCA_TH_17	Jusakul A *et al*	WGS	*TP53*	17	7577539	7577539	G	A	SNV	R248W	Missense
CCA_TH_17	Jusakul A *et al*	WGS	*SMAD4*	18	48575200	48575200	C	G	SNV	H132D	Missense
CCA_TH_17	Jusakul A *et al*	WGS	*KRAS*	12	25398284	25398284	C	G	SNV	G12A	Missense
CCA_TH_16	Jusakul A *et al*	WGS	*TP53*	17	7578205	7578205	C	T	SNV	S215N	Missense
CCA_TH_16	Jusakul A *et al*	WGS	*KRAS*	12	25398284	25398284	C	T	SNV	G12D	Missense
CCA_TH_14	Jusakul A *et al*	WGS	*SMAD4*	18	48603146	48603149	AGTA	-	Deletion	Splice site	Splice site
CCA_TH_14	Jusakul A *et al*	WGS	*PTEN*	10	89692980	89692980	A	G	SNV	Y155C	Missense
CCA_TH_14	Jusakul A *et al*	WGS	*KRAS*	12	25398281	25398281	C	T	SNV	G13D	Missense
CCA_TH_14	Jusakul A *et al*	WGS	*EPHA2*	1	16464769	16464769	C	T	SNV	Splice site	Splice site
CCA_TH_11	Jusakul A *et al*	WGS	*BRAF*	7	140481417	140481417	C	A	SNV	G464V	Missense
CCA_TH_10	Jusakul A *et al*	WGS	*TP53*	17	7577120	7577120	C	T	SNV	R273H	Missense
CCA_TH_10	Jusakul A *et al*	WGS	*SMAD4*	18	48604764	48604765	-	A	Insertion	H530 fs	Frameshift
CCA_TH_10	Jusakul A *et al*	WGS	*KMT2C*	7	151878863	151878863	G	A	SNV	R2028*	Nonsense
CCA_TH_10	Jusakul A *et al*	WGS	*FGFR2*	10	123279677	123279677	G	C	SNV	S252W	Missense
CCA_TH_1	Jusakul A *et al*	WGS	*SMAD4*	18	48604837	48604837	A	G	SNV	*553W	Stop loss
CCA_TH_1	Jusakul A *et al*	WGS	*PIK3CA*	3	178922324	178922324	G	A	SNV	E365K	Missense
CCA_TH_1	Jusakul A *et al*	WGS	*KMT2D*	12	49416438	49416438	C	T	SNV	E5425K	Missense

CCA_SG_9	Jusakul A *et al*	WGS	*FGFR2*	10	123274794	123274794	T	C	SNV	Y376C	Missense
CCA_SG_9	Jusakul A *et al*	WGS	*EPHA2*	1	16459839	16459860	CCCTTGTACACCTCCCCAAACT	-	Deletion	E623 fs	Frameshift
CCA_SG_8	Jusakul A *et al*	WGS	*KRAS*	12	25398284	25398284	C	T	SNV	G12D	Missense
CCA_SG_6	Jusakul A *et al*	WGS	*FGFR2*	10					Fusion	FGFR2–9p24.2	Fusion
CCA_SG_6	Jusakul A *et al*	WGS	*BAP1*	3	52437627	52437627	G	A	SNV	R512C	Missense
CCA_SG_6	Jusakul A *et al*	WGS	*BAP1*	3	52437801	52437802	-	T	Insertion	E454 fs	Frameshift
CCA_SG_6	Jusakul A *et al*	WGS	*ARID1A*	1	27089777	27089777	G	T	SNV	Splice site	Splice site
CCA_SG_5	Jusakul A *et al*	WGS	*KRAS*	12	25398284	25398284	C	T	SNV	G12D	Missense
CCA_SG_5	Jusakul A *et al*	WGS	*CDKN2A*	9	21971028	21971028	C	T	SNV	W110*	Nonsense
CCA_SG_5	Jusakul A *et al*	WGS	*BRCA2*	13	32907431	32907431	C	A	SNV	P606T	Missense
CCA_SG_4	Jusakul A *et al*	WGS	*BAP1*	3	52437650	52437650	T	-	Deletion	N504 fs	Frameshift
CCA_SG_3	Jusakul A *et al*	WGS	*IDH1*	2	209113113	209113113	G	A	SNV	R132C	Missense
CCA_SG_3	Jusakul A *et al*	WGS	*ARID1A*	1	27097621	27097622	-	A	Insertion	W1073 fs	Frameshift
CCA_SG_20	Jusakul A *et al*	WGS	*IDH2*	15	90631839	90631839	T	A	SNV	R172W	Missense
CCA_SG_2	Jusakul A *et al*	WGS	*IDH1*	2	209113112	209113112	C	A	SNV	R132L	Missense
CCA_SG_2	Jusakul A *et al*	WGS	*EPHA2*	1	16475431	16475431	C	A	SNV	G89*	Nonsense
CCA_SG_2	Jusakul A *et al*	WGS	*BAP1*	3	52439880	52439886	CTTGAGA	-	Deletion	Q277 fs	Frameshift
CCA_SG_19	Jusakul A *et al*	WGS	*FGFR2*	10	123279605	123279605	A	C	SNV	F276C	Missense
CCA_SG_16	Jusakul A *et al*	WGS	*FGFR2*	10					Fusion	FGFR2–10q23.1	Fusion
CCA_SG_15	Jusakul A *et al*	WGS	*IDH1*	2	209113113	209113113	G	A	SNV	R132C	Missense
CCA_SG_15	Jusakul A *et al*	WGS	*FGFR2*	10	123274794	123274794	T	C	SNV	Y376C	Missense
CCA_SG_14	Jusakul A *et al*	WGS	*APC*	5	112175951	112175952	-	A	Insertion	T1556 fs	Frameshift
CCA_SG_12	Jusakul A *et al*	WGS	*FGFR2*	10					Fusion	FGFR2-STK26	Fusion
CCA_SG_10	Jusakul A *et al*	WGS	*FGFR2*	10					Fusion	FGFR2-TBC1D1	Fusion
CCA_SG_1	Jusakul A *et al*	WGS	*TP53*	17	7578457	7578457	C	T	SNV	R158H	Missense
CCA_RO_6	Jusakul A *et al*	WGS	*PTEN*	10	89720712	89720712	A	-	Deletion	E288 fs	Frameshift
CCA_RO_6	Jusakul A *et al*	WGS	*FGFR2*	10					Fusion	FGFR2-WAC	Fusion
CCA_RO_5	Jusakul A *et al*	WGS	*IDH1*	2	209113113	209113113	G	A	SNV	R132C	Missense
CCA_RO_5	Jusakul A *et al*	WGS	*ARID1A*	1	27092770	27092770	G	T	SNV	G931*	Nonsense
CCA_RO_1	Jusakul A *et al*	WGS	*TP53*	17	7577120	7577120	C	T	SNV	R273H	Missense
CCA_RO_1	Jusakul A *et al*	WGS	*KRAS*	12	25398281	25398281	C	T	SNV	G13D	Missense
CCA_IT_4	Jusakul A *et al*	WGS	*SMAD4*	18	48591930	48591930	G	A	SNV	G365S	Missense
CCA_IT_4	Jusakul A *et al*	WGS	*KMT2C*	7	151878785	151878785	G	A	SNV	Q2054*	Nonsense

CCA_IT_3	Jusakul A *et al*	WGS	*TP53*	17	7578370	7578370	C	T	SNV	Splice site	Splice site
CCA_IT_3	Jusakul A *et al*	WGS	*SMAD4*	18	48604764	48604765	-	A	Insertion	H530 fs	Frameshift
CCA_IT_3	Jusakul A *et al*	WGS	*KRAS*	12	25398284	25398284	C	A	SNV	G12V	Missense
CCA_IT_3	Jusakul A *et al*	WGS	*FGFR2*	10	123278289	123278289	C	T	SNV	E332K	Missense
CCA_IT_2	Jusakul A *et al*	WGS	*APC*	5	112175969	112175970	-	A	Insertion	D1562 fs	Frameshift
CCA_IT_1	Jusakul A *et al*	WGS	*TP53*	17	7577022	7577022	G	A	SNV	R306*	Nonsense
CCA_IT_1	Jusakul A *et al*	WGS	*APC*	5	112176020	112176020	G	T	SNV	E1577*	Nonsense
CCA_CH_8	Jusakul A *et al*	WGS	*TP53*	17	7578442	7578442	T	C	SNV	Y163C	Missense
CCA_CH_8	Jusakul A *et al*	WGS	*KRAS*	12	25398284	25398284	C	A	SNV	G12V	Missense
CCA_CH_7	Jusakul A *et al*	WGS	*TP53*	17	7577498	7577498	C	G	SNV	Splice site	Splice site
CCA_CH_7	Jusakul A *et al*	WGS	*SMAD4*	18	48604632	48604632	C	G	SNV	S485*	Nonsense
CCA_CH_7	Jusakul A *et al*	WGS	*KRAS*	12	25398284	25398284	C	T	SNV	G12D	Missense
CCA_CH_7	Jusakul A *et al*	WGS	*KMT2D*	12	49438197	49438197	G	-	Deletion	P1691 fs	Frameshift
CCA_CH_6	Jusakul A *et al*	WGS	*CDKN2A*	9	21968243	21968243	T	C	SNV	Splice site	Splice site
CHOL54	Jiao Y *et al*	WES	*PBRM1*	3	52713603	52713603	G	-	Deletion	P42 fs	Frameshift
CHOL52	Jiao Y *et al*	WES	*IDH2*	15	90631838	90631838	C	T	SNV	R172K	Missense
CHOL52	Jiao Y *et al*	WES	*BAP1*	3	52437492	52437495	TGAC	-	Deletion	V556 fs	Frameshift
CHOL48	Jiao Y *et al*	WES	*PBRM1*	3	52643768	52643768	G	A	SNV	R710*	Nonsense
CHOL44	Jiao Y *et al*	WES	*TGFBR2*	3	30666876	30666876	A	-	Deletion	K128 fs	Frameshift
CHOL44	Jiao Y *et al*	WES	*IDH1*	2	209113113	209113113	G	C	SNV	R132G	Missense
CHOL42	Jiao Y *et al*	WES	*PIK3CA*	3	178916944	178916944	A	G	SNV	K111E	Missense
CHOL42	Jiao Y *et al*	WES	*FGFR2*	10	123274774	123274774	A	G	SNV	C383R	Missense
CHOL42	Jiao Y *et al*	WES	*EPHA2*	1	16464417	16464417	G	-	Deletion	R415 fs	Frameshift
CHOL42	Jiao Y *et al*	WES	*BAP1*	3	52437846	52437846	C	A	SNV	V439L	Missense
CHOL37	Jiao Y *et al*	WES	*BAP1*	3	52441263	52441263	G	C	SNV	H169Q	Missense
CHOL37	Jiao Y *et al*	WES	*ARID1A*	1	27023543	27023547	CCCAA	-	Deletion	N218 fs	Frameshift
CHOL28	Jiao Y *et al*	WES	*FGFR2*	10	123247623	123247625	GAT	-	Deletion	H624 del	Inframe deletion
CHOL26	Jiao Y *et al*	WES	*BAP1*	3	52438487	52438488	-	T	Insertion	N411 fs	Frameshift
CHOL24	Jiao Y *et al*	WES	*PIK3CA*	3	178952085	178952085	A	T	SNV	H1047L	Missense
CHOL24	Jiao Y *et al*	WES	*EPHA2*	1	16464672	16464672	A	-	Deletion	S330 fs	Frameshift
CHOL24	Jiao Y *et al*	WES	*ARID1A*	1	27089489	27089489	T	G	SNV	Y815*	Nonsense
CHOL20	Jiao Y *et al*	WES	*PBRM1*	3	52620696	52620696	A	-	Deletion	F1044 fs	Frameshift
CHOL19	Jiao Y *et al*	WES	*PTEN*	10	89682884	89682884	C	T	SNV	R130*	Nonsense
CHOL18	Jiao Y *et al*	WES	*PBRM1*	3	52643782	52643785	TCCA	-	Deletion	M704 fs	Frameshift
CHOL18	Jiao Y *et al*	WES	*ARID1A*	1	27101541	27101541	A	G	SNV	H1608R	Missense

CHOL15	Jiao Y *et al*	WES	*IDH1*	2	209113113	209113113	G	A	SNV	R132C	Missense
CHOL15	Jiao Y *et al*	WES	*ARID1A*	1	27105553	27105553	C	T	SNV	R1722*	Nonsense
CHOL14	Jiao Y *et al*	WES	*PTEN*	10	89720833	89720833	A	-	Deletion	N329 fs	Frameshift
CHOL14	Jiao Y *et al*	WES	*IDH2*	15	90631837	90631837	C	A	SNV	R172S	Missense
CHOL14	Jiao Y *et al*	WES	*ARID1A*	1	27057919	27057919	C	A	SNV	Q543K	Missense
CHOL13	Jiao Y *et al*	WES	*KRAS*	12	25398284	25398284	C	T	SNV	G12D	Missense
CHOL12	Jiao Y *et al*	WES	*TP53*	17	7577084	7577084	T	A	SNV	E285V	Missense
CHOL12	Jiao Y *et al*	WES	*ATM*	11	108216576	108216576	C	T	SNV	P2842L	Missense
CHOL09	Jiao Y *et al*	WES	*IDH1*	2	209113113	209113113	G	T	SNV	R132S	Missense
CHOL09	Jiao Y *et al*	WES	*FGFR2*	10	123274794	123274794	T	C	SNV	Y376C	Missense
CHOL09	Jiao Y *et al*	WES	*BAP1*	3	52443857	52443857	C	T	SNV	Splice site	Splice site
CHOL08	Jiao Y *et al*	WES	*TP53*	17	7578232	7578232	A	T	SNV	L206*	Nonsense
CHOL08	Jiao Y *et al*	WES	*PBRM1*	3	52610608	52610609	-	T	Insertion	E1214 fs	Frameshift
CHOL08	Jiao Y *et al*	WES	*BAP1*	3	52440354	52440354	A	-	Deletion	V233 fs	Frameshift
CHOL08	Jiao Y *et al*	WES	*ARID1A*	1	27058057	27058057	C	T	SNV	Q589*	Nonsense
CHOL07	Jiao Y *et al*	WES	*IDH1*	2	209113113	209113113	G	A	SNV	R132C	Missense
CHOL07	Jiao Y *et al*	WES	*FGFR2*	10	123279605	123279605	A	C	SNV	F276C	Missense
CHOL06	Jiao Y *et al*	WES	*ATM*	11	108114819	108114819	T	G	SNV	F213L	Missense
CHOL04	Jiao Y *et al*	WES	*CDKN2A*	9	21971209	21971209	G	C	SNV	Splice site	Splice site
CHOL04	Jiao Y *et al*	WES	*BAP1*	3	52441999	5242002	TCC	-	Deletion	K116_D117 delinsN	Inframe deletion
CHOL01	Jiao Y *et al*	WES	*NRAS*	1	115258745	115258745	C	G	SNV	G13R	Missense
CHOL01	Jiao Y *et al*	WES	*NF1*	17	29509536	29509536	A	-	Deletion	K248 fs	Frameshift
CHOL01	Jiao Y *et al*	WES	*BAP1*	3	52439929	52439929	C	T	SNV	Splice site	Splice site
7913T	Gao Q *et al*	WES	*FGFR2*	10	123246869	123246869	C	T	SNV	V687I	Missense
4139T	Gao Q *et al*	WES	*FBXW7*	4	153252012	153252013	-	TGAA	Insertion	E214 fs	Frameshift
RK312	Fujimoto A *et al*	WGS	*PTEN*	10	89720778	89720778	A	G	SNV	D310G	Missense
RK312	Fujimoto A *et al*	WGS	*IDH1*	2	209113113	209113113	G	A	SNV	R132C	Missense
RK312	Fujimoto A *et al*	WGS	*EPHA2*	1	16459707	16459708	-	A	Insertion	N674 fs	Frameshift
RK310	Fujimoto A *et al*	WGS	*PIK3CA*	3	178936082	178936082	G	A	SNV	E542K	Missense
RK310	Fujimoto A *et al*	WGS	*IDH2*	15	90631839	90631839	T	A	SNV	R172W	Missense
RK310	Fujimoto A *et al*	WGS	*BAP1*	3	52440866	52440887	CGCTCCAT GATGACCC	-	Deletion	I214 fs	Frameshift

							GCCGGG				
RK303	Fujimoto A *et al*	WGS	*BRCA2*	13	32937642	32937642	T	C	SNV	L2768P	Missense
RK303	Fujimoto A *et al*	WGS	*BRCA2*	13	32914286	32914286	C	A	SNV	H1932N	Missense
RK303	Fujimoto A *et al*	WGS	*ARID1A*	1	27099976	27099977	-	T	Insertion	Y1285 fs	Frameshift
RK303	Fujimoto A *et al*	WGS	*APC*	5	112177286	112177286	C	G	SNV	P1981A	Missense
RK298	Fujimoto A *et al*	WGS	*TP53*	17	7578285	7578295	CAGACCTA AGA	-	Deletion	G187 fs	Frameshift
RK298	Fujimoto A *et al*	WGS	*PTEN*	10	89692911	89692911	G	T	SNV	G132V	Missense
RK298	Fujimoto A *et al*	WGS	*KMT2C*	7	151877881	151877881	A	C	SNV	L2355R	Missense
RK279	Fujimoto A *et al*	WGS	*FGFR2*	10					Fusion	FGFR2-BICC1	Fusion
RK279	Fujimoto A *et al*	WGS	*CDKN2A*	9	21971120	21971120	G	A	SNV	R80*	Nonsense
RK279	Fujimoto A *et al*	WGS	*BAP1*	3	52441268	52441268	A	G	SNV	F168L	Missense
RK272	Fujimoto A *et al*	WGS	*ATM*	11	108196197	108196197	G	C	SNV	E2245Q	Missense
RK272	Fujimoto A *et al*	WGS	*ARID1A*	1	27099046	27099046	G	-	Deletion	M1154 fs	Frameshift
RK269	Fujimoto A *et al*	WGS	*PBRM1*	3	52610639	52610640	CT	-	Deletion	E1178 fs	Frameshift
RK269	Fujimoto A *et al*	WGS	*EPHA2*	1	16462199	16462199	G	-	Deletion	P460 fs	Frameshift
RK226	Fujimoto A *et al*	WGS	*ATM*	11	108106546	108106546	C	A	SNV	Q161K	Missense
RK226	Fujimoto A *et al*	WGS	*ARID2*	12	46246341	46246341	C	T	SNV	Q1479*	Nonsense
RK194	Fujimoto A *et al*	WGS	*KRAS*	12	25398284	25398284	C	T	SNV	G12D	Missense
RK182	Fujimoto A *et al*	WGS	*TP53*	17	7577555	7577564	GCAGGAA CTG	-	Deletion	N239 fs	Frameshift
RK146	Fujimoto A *et al*	WGS	*KRAS*	12	25380275	25380275	T	G	SNV	Q61H	Missense
RK146	Fujimoto A *et al*	WGS	*ARID2*	12	46244470	46244470	C	T	SNV	T855I	Missense
RK138	Fujimoto A *et al*	WGS	*PIK3CA*	3	178928079	178928079	G	A	SNV	E453K	Missense

OS01	Fujimoto A *et al*	WES	*TP53*	17	7577142	7577142	C	T	SNV	G266R	Missense
OS01	Fujimoto A *et al*	WES	*KRAS*	12	25398284	25398284	C	A	SNV	G12V	Missense
HK22	Fujimoto A *et al*	WES	*IDH1*	2	209113113	209113113	G	C	SNV	R132G	Missense
HK16	Fujimoto A *et al*	WES	*KRAS*	12	25398284	25398284	C	A	SNV	G12V	Missense
HK15	Fujimoto A *et al*	WES	*PBRM1*	3	52610695	52610695	G	A	SNV	R1185*	Nonsense
HK15	Fujimoto A *et al*	WES	*ARID2*	12	46242736	46242737	-	T	Insertion	F567 fs	Frameshift
HK14	Fujimoto A *et al*	WES	*IDH2*	15	90631839	90631839	T	A	SNV	R172W	Missense
HK14	Fujimoto A *et al*	WES	*BAP1*	3	52442095	52442095	T	C	SNV	Splice site	Splice site
HK13	Fujimoto A *et al*	WES	*APC*	5	112162809	112162809	A	-	Deletion	L472 fs	Frameshift
HK10	Fujimoto A *et al*	WES	*TP53*	17	7578502	7578502	-	CAGGG	Insertion	P142 fs	Frameshift
HK10	Fujimoto A *et al*	WES	*KRAS*	12	25398284	25398284	C	T	SNV	G12D	Missense
HK10	Fujimoto A *et al*	WES	*KMT2D*	12	49446379	49446379	GGTTCCTTG	-	Deletion	P406 fs	Frameshift
HK10	Fujimoto A *et al*	WES	*ARID2*	12	46233115	46233131	TGTTAGTGTGTCTGGTT	-	Deletion	M445 fs	Frameshift
HK03	Fujimoto A *et al*	WES	*TGFBR2*	3	30732970	30732970	G	A	SNV	R553H	Missense
HK03	Fujimoto A *et al*	WES	*NF1*	17	29562641	29562641	C	T	SNV	R1241*	Nonsense
HK03	Fujimoto A *et al*	WES	*ARID1A*	1	27106504	27106504	C	T	SNV	Q2039*	Nonsense
HK02	Fujimoto A *et al*	WES	*TP53*	17	7577509	7577509	C	T	SNV	E258K	Missense
HK02	Fujimoto A *et al*	WES	*PIK3CA*	3	178936091	178936091	G	A	SNV	E545K	Missense
ICC014	CCGC	WES	*KRAS*	12	25398284	25398284	C	A	SNV	G12V	Missense
ICC014	CCGC	WES	*FBXW7*	4	153332623	153332623	C	A	SNV	E111D	Missense
ICC014	CCGC	WES	*ARID1A*	1	27101708	27101708	A	T	SNV	I1664F	Missense
ICC013	CCGC	WES	*TP53*	17	7577534	7577534	C	A	SNV	R249S	Missense
ICC012	CCGC	WES	*KRAS*	12	25380275	25380275	T	G	SNV	Q61H	Missense

ICC011	CCGC	WES	*PBRM1*	3	52627272	52627272	G	C	SNV	Splice site	Splice site
ICC006	CCGC	WES	*KRAS*	12	25398285	25398285	C	A	SNV	G12C	Missense
ICC005	CCGC	WES	*IDH2*	15	90631839	90631839	T	C	SNV	R172G	Missense
ICC004	CCGC	WES	*TP53*	17	7577098	7577098	T	A	SNV	R280S	Missense
ICC004	CCGC	WES	*NRAS*	1	115256529	115256529	T	C	SNV	Q61R	Missense
ICC002	CCGC	WES	*NRAS*	1	115256528	115256528	T	A	SNV	Q61H	Missense
ICC002	CCGC	WES	*CDKN2A*	9	21970901	21970901	G	C	SNV	G102A	Missense
6	Borad MJ *et al*	WES	*FGFR2*	10					Fusion	FGFR2-TACC3	Fusion
6	Borad MJ *et al*	WES	*BAP1*	3	52442567	52442567	G	A	SNV	R60*	Nonsense
5	Borad MJ *et al*	WES	*FGFR2*	10					Fusion	FGFR2-BICC1	Fusion
4	Borad MJ *et al*	WES	*PBRM1*	3	52621409	52621409	A	T	SNV	V1028D	Missense
4	Borad MJ *et al*	WES	*FGFR2*	10					Fusion	FGFR2-MGEA5	Fusion
2	Borad MJ *et al*	WES	*TP53*	17	7577120	7577120	C	T	SNV	R273H	Missense
1	Borad MJ *et al*	WES	*NRAS*	1	115258745	115258745	C	G	SNV	G13R	Missense
1	Borad MJ *et al*	WES	*KMT2C*	7	151853290	151853290	C	T	SNV	V3938I	Missense
1	Borad MJ *et al*	WES	*IDH2*	15	90631839	90631839	T	A	SNV	R172W	Missense

Table S7: List of somatic nonsilent single nucleotide variations and indels of the targeted genes in the FUDAN-NGS cohort

Sample ID	Gene Symbol	Chromosome	Ref Seq	Start position	End position	Reference Allele	Variant Allele	Nucleotide Change	Amino acid change	Mutation type
ICC-4	*KRAS*	chr12	NM_004985	25398284	25398284	C	T	c.35G>A	p.G12D	Missense
ICC-4	*IRS2*	chr13	NM_003749	110436296	110436296	-	CGGCGGCGG	c.2096_2104dup	p.A699_A701dup	Inframe insertion
ICC-6	*RNF43*	chr17	NM_017763	56492730	56492730	G	A	c.209C>T	p.A70V	Missense
ICC-6	*PBRM1*	chr3	NM_018313	52637700	52637700	A	-	c.2616del	p.F872Lfs*43	Frameshift
ICC-6	*KRAS*	chr12	NM_004985	25380275	25380275	T	G	c.183A > C	p.Q61H	Missense
ICC-7	*PBRM1*	chr3	NM_018313	52682399	52682399	-	T	c.773dup	p.N258Kfs*6	Frameshift
ICC-7	*KRAS*	chr12	NM_004985	25380275	25380275	T	G	c.183A>C	p.Q61H	Missense
ICC-7	*FAT1*	chr4	NM_00524	187539233	187539233	G	A	c.8507C>T	p.A2836	Missense

			5						V	
ICC-17	*PTEN*	chr10	NM_000314	89720811	89720811	-	A	c.968dup	p.N323Kfs*2	Frameshift
ICC-17	*PIK3CA*	chr3	NM_006218	178936091	178936091	G	A	c.1633G>A	p.E545K	Missense
ICC-17	*ATM*	chr11	NM_000051	108178644	108178678	TGCTGTTTGGATAAAAAATCACAAAGAACAATGCT	-	c.5702_5736del	p.L1901Cfs*17	Frameshift
ICC-17	*ATM*	chr11	NM_000051	108172509	108172509	-	A	c.5318dup	p.F1774Vfs*8	Frameshift
ICC-17	*ARID1A*	chr1	NM_006015	27106123	27106124	GA	TTG	c.5734_5735delinsTTG	p.D1912Lfs*2	Frameshift
ICC-17	*APC*	chr5	NM_000038	112128155	112128155	A	T	c.658A>T	p.R220*	Nonsense
ICC-22	*TP53*	chr17	NM_001126112	7577096	7577096	T	A	c.842A>T	p.D281V	Missense
ICC-22	*TERT*	chr5	NM_198253	1295228	1295228	G	A	c.-124C>T	N/A	Promoter
ICC-22	*PIK3R1*	chr5	NM_181523	67576765	67576803	ACTGAAAACCTCATAAAAGTTATAGAAATTTTAATCTCA	-	c.853_891del	p.N285_E297del	Inframe deletion
ICC-22	*NRG3*	chr10	NM_0010100848	84711287	84711287	A	T	c.1117A>T	p.I373F	Missense
ICC-22	*NPM1*	chr5	NM_002520	170827174	170827174	T	A	c.542T>A	p.F181Y	Missense
ICC-22	*NPM1*	chr5	NM_002520	170827173	170827173	T	G	c.541T>G	p.F181V	Missense
ICC-22	*NOTCH2*	chr1	NM_024408	120484321	120484321	A	T	c.2809T>A	p.C937S	Missense
ICC-22	*NF1*	chr17	NM_001042492	29550490	29550490	A	T	c.1750A>T	p.K584*	Nonsense
ICC-22	*ERBB4*	chr2	NM_005235	212530104	212530104	G	T	c.1815C>A	p.F605L	Missense
ICC-22	*CREBBP*	chr16	NM_004380	3900497	3900497	T	A	c.599A>T	p.Q200L	Missense
ICC-22	*CHD2*	chr15	NM_001271	93555631	93555631	A	T	c.4649A>T	p.K1550M	Missense
ICC-23	*TP53*	chr17	NM_001126112	7573977	7573985	GAGTTCCAA	-	c.1042_1050del	p.L348_L350del	Inframe deletion
ICC-23	*TERT*	chr5	NM_198253	1295228	1295228	G	A	c.-124C>T	N/A	Promoter
ICC-23	*PALB2*	chr16	NM_02467	23646541	23646541	A	T	c.1326T>A	p.N442K	Missense

			5							
ICC-23	*ATM*	chr11	NM_000051	108199798	108199799	AA	-	c.7141_7142del	p.N2381Wfs*21	Frameshift
ICC-23	*ARID1B*	chr6	NM_017519	157454197	157454197	G	T	c.2368G>T	p.A790S	Missense
ICC-23	*ARID1A*	chr1	NM_006015	27107083	27107093	CGGCGGGCTGC	-	c.6695_6705del	p.R2232Pfs*42	Frameshift
ICC-24	*WT1*	chr11	NM_024426	32450105	32450105	G	A	c.707C>T	p.A236V	Missense
ICC-24	*TGFBR1*	chr9	NM_004612	101900362	101900362	G	A	c.796G>A	p.D266N	Missense
ICC-24	*PIK3R1*	chr5	NM_181523	67589593	67589593	-	AAC	c.1358_1360dup	p.N453dup	Inframe insertion
ICC-24	*PIK3R1*	chr5	NM_181523	67589607	67589609	AAG	-	c.1372_1374del	p.E458del	Inframe deletion
ICC-24	*NF1*	chr17	NM_001042492	29676201	29676202	CT	-	c.7255_7256del	p.L2419Gfs*2	Frameshift
ICC-24	*CDKN2A*	chr9	NM_000077	21971120	21971120	G	A	c.238C>T	p.R80*	Nonsense
ICC-24	*APC*	chr5	NM_000038	112175951	112175951	-	A	c.4666dup	p.T1556Nfs*3	Frameshift
ICC-32	*ZNF703*	chr8	NM_025069	37555296	37555296	G	A	c.877G>A	p.V293M	Missense
ICC-32	*TP53*	chr17	NM_001126112	7577124	7577124	C	T	c.814G>A	p.V272M	Missense
ICC-32	*TP53*	chr17	NM_001126112	7579578	7579578	A	-	c.109del	p.S37Pfs*7	Frameshift
ICC-32	*SPTA1*	chr1	NM_003126	158650477	158650477	G	A	c.574C>T	p.R192C	Missense
ICC-32	*LRP1B*	chr2	NM_018557	141460115	141460115	A	G	c.6031T>C	p.F2011L	Missense
ICC-32	*FGF19*	chr11	NM_005117						gain	Copy number gain
ICC-32	*CCND1*	chr11	NM_053056						gain	Copy number gain
ICC-32	*BRIP1*	chr17	NM_032043	59761461	59761461	T	A	c.2946A>T	p.K982N	Missense
ICC-32	*ARID2*	chr12	NM_152641	46230562	46230562	C	TT	c.811delinsTT	p.H271Lfs*9	Frameshift
ICC-32	*AKT2*	chr19	NM_001626						gain	Copy number gain
ICC-33	*WT1*	chr11	NM_024426	32410686	32410686	A	G	c.1472A>G	p.K491R	Missense

ICC-33	*SPEN*	chr1	NM_015001	16259649	16259651	CAG	-	c.6915_6917delCAG	p.S2306del	Inframe deletion
ICC-33	*SDHD*	chr11	NM_003002	111965550	111965553	GACT	-	c.337_340delGACT	p.D113fs*21	Frameshift
ICC-33	*RNF43*	chr17	NM_017763	56435160	56435160	G	-	c.1976delG	p.G659fs*41	Frameshift
ICC-33	*RB1*	chr13	NM_000321	48923136	48923136	G	A	c.584G>A	p.W195*	Nonsense
ICC-33	*PTEN*	chr10	NM_000314	89720816	89720816	A	-	c.968delA	p.N323fs*21	Frameshift
ICC-33	*PTEN*	chr10	NM_000314						loss	Copy number loss
ICC-33	*POLD1*	chr19	NM_002691	50905537	50905537	C	T	c.665C>T	p.P222L	Missense
ICC-33	*PIK3C2B*	chr1	NM_002646	204399069	204399069	G	A	c.4378G>A	p.A1460T	Missense
ICC-33	*PBRM1*	chr3	NM_018313	52637555	52637555	C	T	c.2761C>T	p.R921*	Nonsense
ICC-33	*NOTCH1*	chr9	NM_017617	139409083	139409083	G	A	c.2086G>A	p.G696S	Missense
ICC-33	*MLH1*	chr3	NM_000249	37035148	37035148	A	G	c.110A>G	p.E37G	Missense
ICC-33	*MED12*	chrX	NM_005120	70356437	70356437	A	G	c.5332A>G	p.S1778G	Missense
ICC-33	*KRAS*	chr12	NM_004985	25398284	25398284	G	T	c.35G>T	p.G12V	Missense
ICC-33	*IGF1R*	chr15	NM_000875	99454583	99454583	C	T	c.1502C>T	p.S501L	Missense
ICC-33	*FBXW7*	chr4	NM_033632	153247158	153247158	G	T	c.1644G>T	p.Q548H	Missense
ICC-33	*EGFR*	chr7	NM_005228	55229314	55229314	C	T	c.1621C>T	p.L541F	Missense
ICC-33	*BCORL1*	chrX	NM_021946	129190016	129190016	C	-	c.5042delC	p.P1681fs*20	Frameshift
ICC-33	*ATR*	chr3	NM_001184	142215345	142215345	T	C	c.5756T>C	p.M1919T	Missense
ICC-33	*ARID1A*	chr1	NM_006015	27100180	27100180	C	-	c.3977delC	p.P1326fs*155	Frameshift
ICC-33	*ARAF*	chrX	NM_001654	47426390	47426390	G	A	c.733G>A	p.G245S	Missense
ICC-33	*APC*	chr5	NM_000038	112179713	112179713	C	T	c.8422C>T	p.P2808S	Missense
ICC-35	*TP53*	chr17	NM_0011126112	7578406	7578406	C	T	c.524G>A	p.R175H	Missense

ICC-35	*TP53*	chr17	NM_001126112	7579872	7579873	AG	-	c.40_41del	p.L14Efs*14	Frameshift
ICC-35	*KRAS*	chr12	NM_004985	25398284	25398284	C	A	c.35G>T	p.G12V	Missense
ICC-35	*CDKN2A*	chr9	NM_000077	21974757	21974775	GACCCCGGGCCGCGGCCGT	-	c.52_70del	p.T18Gfs*2	Frameshift
ICC-41	*KRAS*	chr12	NM_004985	25398284	25398284	G	T	c.35G>T	p.G12V	Missense
ICC-41	*ARID1A*	chr1	NM_006015	27101609	27101609	-	AGCC	c.4892_4895dupAGCC	p.P1633Afs*16	Frameshift
ICC-42	*TP53*	chr17	NM_001126112	7577099	7577099	G	A	c.839G>A	p.R280K	Missense
ICC-42	*TEK*	chr9	NM_000459	27206580	27206580	A	G	c.2365A>G	p.R789G	Missense
ICC-42	*PTEN*	chr10	NM_000314						loss	Copy number loss
ICC-42	*MDM2*	chr12	NM_002392						gain	Copy number gain
ICC-42	*FLT1*	chr13	NM_001160030	28942727	28942727	C	-	c.2189delC	p.S730fs*43	Frameshift
ICC-42	*ERBB2*	chr17	NM_004448	37881392	37881392	A	G	c.2584A>G	p.T862A	Missense
ICC-42	*BRCA2*	chr13	NM_000059	32915230	32915240	AGTCATGCCA	-	c.6739_6748delAGTCATGCCA	p.S2247fs*30	Frameshift
ICC-42	*BRCA1*	chr17	NM_007294	41246625	41246625	G	A	c.923G>A	p.S308N	Missense
ICC-59	*TSC2*	chr16	NM_000548	2121870	2121870	G	A	c.2032G>A	p.A678T	Missense
ICC-59	*TNFAIP3*	chr6	NM_006290	138192514	138192514	G	T	c.150G>T	p.M50I	Missense
ICC-59	*SPEN*	chr1	NM_015001	16263740	16263740	C	T	c.10109C>T	p.P3370L	Missense
ICC-59	*MSH6*	chr2	NM_000179	48010524	48010524	G	T	c.152G>T	p.S51I	Missense
ICC-59	*IKBKE*	chr1	NM_014002	206647732	206647732	G	T	c.146G>T	p.R49L	Missense
ICC-59	*IDH2*	chr15	NM_002168	90631839	90631839	A	G	c.514A>G	p.R172G	Missense
ICC-59	*FLCN*	chr17	NM_144606	17124751	17124751	C	T	c.971C>T	p.A324V	Missense
ICC-59	*CDKN2A*	chr9	NM_000077						loss	Copy number loss

ICC-59	*ATM*	chr11	NM_000051	108196078	108196078	G	A	c.6614G>A	p.W2205*	Nonsense
ICC-63	*TMPRSS2*	chr21	NM_005656	42839684	42839685	CC	AT	c.1443_1444delinsAT	p.D482Y	Missense
ICC-63	*SMAD4*	chr18	NM_005359	48603148	48603148	T	G	c.1447+2T>G	N/A	Splice site
ICC-63	*RAC1*	chr7	NM_006908	6441646	6441646	G	A	c.436G>A	p.A146T	Missense
ICC-63	*NF1*	chr17	NM_001042492	29546113	29546113	G	T	c.1618G>T	p.E540*	Nonsense
ICC-63	*ESR1*	chr6	NM_001122742	152129276	152129276	G	C	c.229G>C	p.G77R	Missense
ICC-63	*AR*	chrX	NM_000044	66765158	66765158	-	GCAGCA	c.234_239dup	p.Q79_Q80dup	Inframe insertion
ICC-67	*SMARCA2*	chr9	NM_003070	2104032	2104032	T	G	c.3155T>G	p.F1052C	Missense
ICC-67	*PBRM1*	chr3	NM_018313	52637690	52637690	G	A	c.2626C>T	p.R876C	Missense
ICC-67	*KMT2C*	chr7	NM_170606	151877073	151877073	G	A	c.7288C>T	p.Q2430*	Nonsense
ICC-67	*IDH1*	chr2	NM_005896	209113113	209113113	G	A	c.394C>T	p.R132C	Missense
ICC-67	*FAM135B*	chr8	NM_015912	139163579	139163579	C	T	c.3139G>A	p.V1047M	Missense
ICC-67	*ARID1A*	chr1	NM_006015	27023716	27023716	G	-	c.827del	p.G276Efs*87	Frameshift
ICC-69	*KDM6A*	chrX	NM_021140	44950093	44950093	-	T	c.3867dup	p.E1290*	Nonsense
ICC-69	*GLI3*	chr7	NM_000168	42064904	42064904	G	T	c.1315C>A	p.P439T	Missense
ICC-69	*DDR2*	chr1	NM_001014796	162749924	162749924	T	-	c.2458del	p.C820Vfs*8	Frameshift
ICC-69	*BRCA1*	chr17	NM_007294	41243048	41243066	ACCTTAAATAACAAAAACA	-	c.4097-17_4098del	N/A	Splice site
ICC-69	*ATRX*	chrX	NM_000489	76776319	76776319	C	T	c.7147G>A	p.D2383N	Missense
ICC-70	*PTCH1*	chr9	NM_000264	98240426	98240426	G	C	c.1258C>G	p.L420V	Missense
ICC-70	*PALB2*	chr16	NM_024675	23646541	23646541	A	T	c.1326T>A	p.N442K	Missense
ICC-71	*TSC1*	chr9	NM_000368	135771988	135771990	GCT	-	c.3127_3129del	p.S1043del	Inframe deletion
ICC-71	*TP53*	chr17	NM_001126112	7578406	7578406	C	T	c.524G>A	p.R175H	Missense

ICC-71	*TGFBR1*	chr9	NM_004612	101900330	101900330	G	A	c.764G>A	p.R255H	Missense
ICC-71	*PTCH1*	chr9	NM_000264	98209617	98209617	G	-	c.3921del	p.R1308Efs*64	Frameshift
ICC-71	*PIK3R1*	chr5	NM_181523	67593338	67593338	T	C	c.2084T>C	p.V695A	Missense
ICC-71	*NTRK3*	chr15	NM_001012338	88420281	88420281	A	G	c.2405T>C	p.V802A	Missense
ICC-71	*NOTCH1*	chr9	NM_017617	139391832	139391832	C	T	c.6359G>A	p.R2120H	Missense
ICC-71	*NF1*	chr17	NM_001042492	29587450	29587450	C	A	c.4494C>A	p.F1498L	Missense
ICC-71	*LZTR1*	chr22	NM_006767	21348253	21348253	C	T	c.1394C>T	p.A465V	Missense
ICC-71	*LRP1B*	chr2	NM_018557	141625245	141625245	C	T	c.4493G>A	p.W1498*	Nonsense
ICC-71	*FLT4*	chr5	NM_002020	180053023	180053023	G	-	c.1267del	p.Q423Rfs*70	Frameshift
ICC-71	*FLCN*	chr17	NM_144997	17129521	17129521	C	T	c.365G>A	p.R122H	Missense
ICC-71	*CHD4*	chr12	NM_001273	6687691	6687693	TCT	-	c.5001_5003del	p.E1668del	Inframe deletion
ICC-71	*CHD4*	chr12	NM_001273	6711546	6711546	T	-	c.218del	p.K73Rfs*129	Frameshift
ICC-71	*CDK12*	chr17	NM_016507	37618494	37618494	C	-	c.174del	p.E59Kfs*33	Frameshift
ICC-71	*AXIN1*	chr16	NM_003502	347748	347748	G	-	c.1758del	p.N587Tfs*118	Frameshift
ICC-71	*ATRX*	chrX	NM_000489	76920223	76920223	G	T	c.3854C>A	p.S1285Y	Missense
ICC-71	*ARID1B*	chr6	NM_017519	157527697	157527697	C	T	c.5383C>T	p.R1795C	Missense
ICC-71	*ARID1A*	chr1	NM_006015	27101402	27101402	C	-	c.4689del	p.M1564*	Nonsense
ICC-71	*ARID1A*	chr1	NM_006015	27105931	27105931	G	-	c.5548del	p.D1850Tfs*33	Frameshift
ICC-71	*ACVR2A*	chr2	NM_001278579	148683686	148683686	A	-	c.1310del	p.K437Rfs*5	Frameshift
ICC-74	*TP53*	chr17	NM_001126112	7578457	7578457	G	T	c.473G>T	p.R158L	Missense
ICC-74	*TEK*	chr9	NM_000459	27212770	27212770	C	T	c.2752C>T	p.R918C	Missense
ICC-74	*ROS1*	chr6	NM_002944	117686345	117686345	C	T	c.2996C>T	p.S999F	Missense

ICC-74	*PTCH1*	chr9	NM_000264	98224149	98224149	G	A	c.2692G>A	p.D898N	Missense
ICC-74	*POLD1*	chr19	NM_002691	50909521	50909521	G	A	c.1325G>A	p.G442D	Missense
ICC-74	*NOTCH1*	chr9	NM_017617	139405123	139405126	CGAC	-	c.2718_2721delCGAC	p.D907fs*271	Frameshift
ICC-74	*NOTCH1*	chr9	NM_017617	139403426	139403426	-	TGCCCCCCAGCCCTCGGCTCACTCAGCACTCTAGTGCTGATGTC	c.3066_3067ins	p.N1023fs*171	Frameshift
ICC-74	*MTOR*	chr1	NM_004958	11190588	11190588	G	A	c.5611G>A	p.E1871K	Missense
ICC-74	*INPP4B*	chr4	NM_003866	143129658	143129658	G	C	c.992G>C	p.S331T	Missense
ICC-74	*GRM3*	chr7	NM_000840	86415984	86415984	T	G	c.876T>G	p.N292K	Missense
ICC-74	*FBXW7*	chr4	NM_033632	153249367	153249367	G	T	c.1411G>T	p.E471*	Nonsense
ICC-74	*CDKN2A*	chr9	NM_058197	21974591	21974591	C	G	c.236C>G	p.A79G	Missense
ICC-74	*CARD11*	chr7	NM_032415	2959246	2959246	G	T	c.2270G>T	p.G757V	Missense
ICC-74	*AR*	chrX	NM_000044	66931343	66931343	T	C	c.1985T>C	p.V662A	Missense
ICC-77	*KRAS*	chr12	NM_004985	25398284	25398284	C	T	c.35G>A	p.G12D	Missense
ICC-77	*CDKN2A*	chr9	NM_000077	21971120	21971120	G	A	c.238C>T	p.R80*	Nonsense
ICC-89	*SMAD4*	chr18	NM_005359						loss	Copy number loss
ICC-89	*KMT2D*	chr12	NM_003482	49428364	49428364	C	A	c.10440+1G>T	N/A	Splice site
ICC-89	*FAM135B*	chr8	NM_015912	139164247	139164247	C	T	c.2471G>A	p.G824E	Missense
ICC-89	*EPHA2*	chr1	NM_004431	16458707	16458707	C	A	c.2177G>T	p.G726V	Missense
ICC-89	*CDKN2B*	chr9	NM_004936						loss	Copy number loss
ICC-89	*CDKN2A*	chr9	NM_000077						loss	Copy number loss
ICC-89	*BRAF*	chr7	NM_004333	140453154	140453154	T	C	c.1781A>G	p.D594G	Missense

ICC-89	*ARID1B*	chr6	NM_017519	157469898	157469898	C	T	c.2653C>T	p.R885*	Nonsense
ICC-89	*ARID1A*	chr1	NM_006015	27024001	27024001	-	G	c.1113dup	p.Q372Afs*28	Frameshift
ICC-91	*TP53*	chr17	NM_001126112	7578242	7578277	CACGCAAATTTCCTTCCACTCGGATAAGATGCTGAG	-	c.572_607del	p.P191_V203delinsL	Inframe deletion and insertion
ICC-91	*KRAS*	chr12	NM_004985	25398284	25398284	C	A	c.35G>T	p.G12V	Missense
ICC-91	*CDKN2A*	chr9	NM_000077	21971006	21971006	C	G	c.352G>C	p.A118P	Missense
ICC-93	*MED12*	chrX	NM_005120	70348996	70348996	C	T	c.3508C>T	p.R1170W	Missense
ICC-93	*IDH2*	chr15	NM_002168	90631839	90631839	T	A	c.514A>T	p.R172W	Missense
ICC-93	*BAP1*	chr3	NM_004656	52443880	52443880	C	T	c.15G>A	p.W5*	Nonsense
ICC-98	*TP53*	chr17	NM_001126112	7574018	7574018	G	A	c.1009C>T	p.R337C	Missense
ICC-98	*KRAS*	chr12	NM_004985	25380275	25380275	T	G	c.183A>C	p.Q61H	Missense
ICC-98	*CTNNB1*	chr3	NM_001904	41266137	41266137	C	T	c.134C>T	p.S45F	Missense
ICC-100	*PTK2*	chr8	NM_005607						gain	Copy number gain
ICC-100	*MYC*	chr8	NM_002467						gain	Copy number gain
ICC-100	*MAP2K4*	chr17	NM_003010						loss	Copy number loss
ICC-100	*KRAS*	chr12	NM_004985	25398284	25398284	C	T	c.35G>A	p.G12D	Missense
ICC-100	*FGFR1*	chr8	NM_023110						gain	Copy number gain
ICC-100	*FAM135B*	chr8	NM_015912						gain	Copy number gain
ICC-118	*TP53*	chr17	NM_001126112	7578203	7578203	C	T	c.646G>A	p.V216M	Missense
ICC-118	*TGFBR2*	chr3	NM_003242	30691871	30691871	-	A	c.383dup	p.P129Afs*3	Frameshift
ICC-118	*KRAS*	chr12	NM_004985	25380275	25380275	T	A	c.183A>T	p.Q61H	Missense
ICC-118	*KRAS*	chr12	NM_004985						gain	Copy number gain

ICC-118	*FGFR1*	chr8	NM_021623-NM_023110						PLEKHA2-FGFR1	Fusion
ICC-118	*FAT1*	chr4	NM_005245	187629360	187629360	G	T	c.1622C>A	p.S541*	Nonsense
ICC-118	*CDKN2B*	chr9	NM_004936						loss	Copy number loss
ICC-118	*CDKN2A*	chr9	NM_000077						loss	Copy number loss
ICC-124	*TGFBR1*	chr9	NM_004612	101900151	101900151	G	T	c.585G>T	p.L195F	Missense
ICC-124	*TGFBR1*	chr9	NM_004612	101900146	101900151	CCATTG	T	c.580_585delinsT	p.P194Sfs*37	Frameshift
ICC-124	*KMT2D*	chr12	NM_003482	49420228	49420228	C	T	c.15521G>A	p.R5174Q	Missense
ICC-124	*IKZF1*	chr7	NM_006060	50459450	50459450	A	G	c.739A>G	p.S247G	Missense
ICC-124	*ARID1A*	chr1	NM_006015	27106133	27106133	T	A	c.5744T>A	p.L1915*	Nonsense
ICC-134	*TGFBR2*	chr3	NM_003242	30732969	30732969	C	T	c.1582C>T	p.R528C	Missense
ICC-134	*TGFBR2*	chr3	NM_003242	30691872	30691874	AAA	-	c.381_383del	p.K128del	Inframe deletion
ICC-134	*SETD2*	chr3	NM_014159	47139549	47139549	A	G	c.5038T>C	p.C1680R	Missense
ICC-134	*PIK3CA*	chr3	NM_006218	178936092	178936092	A	C	c.1634A>C	p.E545A	Missense
ICC-134	*KRAS*	chr12	NM_004985	25398284	25398284	C	T	c.35G>A	p.G12D	Missense
ICC-134	*BRCA2*	chr13	NM_000059	32930634	32930634	G	A	c.7505G>A	p.R2502H	Missense
ICC-134	*ARID1A*	chr1	NM_006015	27023716	27023716	G	-	c.827del	p.G276Efs*87	Frameshift
ICC-137	*TP53*	chr17	NM_001126112	7577018	7577018	C	T	c.919+1G > A	N/A	Splice site
ICC-137	*SMAD4*	chr18	NM_005359	48604778	48604778	C	T	c.1600C > T	p.Q534*	Nonsense
ICC-137	*KRAS*	chr12	NM_004985	25398285	25398285	C	G	c.34G > C	p.G12R	Missense
ICC-137	*FBXW7*	chr4	NM_033632	153249385	153249385	G	A	c.1393C > T	p.R465C	Missense
ICC-139	*NTRK3*	chr15	NM_001012338	88428951	88428951	C	A	c.2149G>T	p.G717*	Nonsense

ICC-139	*NTRK3*	chr15	NM_001012338	88428947	88428947	T	A	c.2153A>T	p.N718I	Missense
ICC-139	*KRAS*	chr12	NM_004985	25398284	25398284	C	T	c.35G>A	p.G12D	Missense
ICC-139	*CREBBP*	chr16	NM_004380	3779476	3779476	G	A	c.5572C>T	p.R1858C	Missense
ICC-139	*ATM*	chr11	NM_000051	108117712	108117712	G	A	c.923G>A	p.W308*	Nonsense
ICC-144	*TP53*	chr17	NM_001126112	7577538	7577538	C	T	c.743G>A	p.R248Q	Missense
ICC-144	*TERT*	chr5	NM_198253	1295228	1295228	G	A	c.-124C>T	N/A	Promoter
ICC-144	*SMARCA2*	chr9	NM_003070						loss	Copy number loss
ICC-144	*RET*	chr10	NM_020975	43612030	43612030	A	G	c.2137-2A>G	N/A	Splice site
ICC-144	*RAD50*	chr5	NM_005732	131923764	131923764	A	T	c.1034A>T	p.E345V	Missense
ICC-144	*PTK6*	chr20	NM_001256358						gain	Copy number gain
ICC-144	*PRDM1*	chr6	NM_001198	106536077	106536077	C	A	c.44C>A	p.A15D	Missense
ICC-144	*MYC*	chr8	NM_002467						gain	Copy number gain
ICC-144	*MAP3K1*	chr5	NM_005921	56176572	56176572	G	A	c.2122G>A	p.E708K	Missense
ICC-144	*LRP1B*	chr2	NM_018557	140995830	140995830	C	T	c.13451G>A	p.G4484E	Missense
ICC-144	*IGF2*	chr11	NM_001291861	2154282	2154282	C	A	c.478G>T	p.A160S	Missense
ICC-144	*FBXW7*	chr4	NM_033632	153245338	153245338	T	A	c.1853A>T	p.Q618L	Missense
ICC-144	*CREBBP*	chr16	NM_004380	3843590	3843590	T	A	c.1013A>T	p.Q338L	Missense
ICC-144	*CHD2*	chr15	NM_001271	93510737	93510737	A	-	c.2183del	p.Y728Ffs*6	Frameshift
ICC-144	*CDKN2B*	chr9	NM_004936						loss	Copy number loss
ICC-144	*CDKN2A*	chr9	NM_000077						loss	Copy number loss
ICC-144	*ARID1A*	chr1	NM_006015	27092719	27092721	GGC	A	c.2740_2742delinsA	p.G914Ifs*21	Frameshift
ICC-149	*SMAD4*	chr18	NM_005359	48591892	48591892	G	A	c.1055G>A	p.G352E	Missense

ICC-149	*KRAS*	chr12	NM_004985	25398284	25398284	C	A	c.35G>T	p.G12V	Missense
ICC-155	*TP53*	chr17	NM_001126112	7579418	7579426	GAGGGGGCT	CAGGGGCC	c.261_269delinsGGCCCCTG	p.S90Afs*33	Frameshift
ICC-155	*SMAD4*	chr18	NM_005359	48575180	48575181	GT	-	c.376_377del	p.V126Lfs*16	Frameshift
ICC-155	*PTPN11*	chr12	NM_002834	112915529	112915529	A	G	c.928A>G	p.I310V	Missense
ICC-155	*KRAS*	chr12	NM_004985	25398284	25398284	C	T	c.35G>A	p.G12D	Missense
ICC-155	*KMT2D*	chr12	NM_003482	49427265	49427265	-	TGC	c.11220_11222dup	p.Q3745dup	Inframe insertion
ICC-155	*KMT2C*	chr7	NM_170606	151875096	151875101	CTTTTA	ATTTT	c.7443-6_7443-1delinsAAAAT	N/A	Splice site
ICC-155	*AR*	chrX	NM_000044	66765164	66765164	A	T	c.176A>T	p.Q59L	Missense
ICC-155	*ABL1*	chr9	NM_005157	133738378	133738378	G	T	c.778G>T	p.V260L	Missense
ICC-161	*PBRM1*	chr3	NM_018313	52696274	52696274	T	A	c.403A>T	p.K135*	Nonsense
ICC-161	*IDH1*	chr2	NM_005896	209113112	209113112	C	A	c.395G>T	p.R132L	Missense
ICC-161	*ATM*	chr11	NM_000051	108236178	108236178	G	T	c.9114G>T	p.Q3038H	Missense
ICC-161	*ARID1A*	chr1	NM_006015	27101611	27101611	-	C	c.4899dup	p.M1634Hfs*14	Frameshift
ICC-165	*TP53*	chr17	NM_001126112	7579585	7579591	GGGGGAC	-	c.97-1_102del	N/A	Splice site
ICC-165	*SPTA1*	chr1	NM_003126	158644458	158644458	G	A	c.1120C>T	p.R374*	Nonsense
ICC-165	*KEAP1*	chr19	NM_012289	10610637	10610637	C	A	c.73G>T	p.E25*	Nonsense
ICC-165	*KEAP1*	chr19	NM_012289	10610363	10610363	C	T	c.347G>A	p.R116Q	Missense
ICC-165	*KDR*	chr4	NM_002253	55948168	55948168	T	A	c.3803A>T	p.E1268V	Missense
ICC-165	*KDM6A*	chrX	NM_021140	44945135	44945135	T	-	c.3460del	p.S1154Qfs*4	Frameshift
ICC-165	*FAT1*	chr4	NM_005245	187518255	187518255	C	T	c.12439G>A	p.G4147S	Missense
ICC-165	*CDKN2B*	chr9	NM_004936						loss	Copy number loss

ICC-165	*CDKN2A*	chr9	NM_000077						loss	Copy number loss
ICC-165	*CDKN1B*	chr12	NM_004064	12871110	12871116	CGCCCGG	-	c.339_345del	p.P114Rfs*3	Frameshift
ICC-165	*ARID2*	chr12	NM_152641	46231286	46231286	G	T	c.1126G>T	p.E376*	Nonsense
ICC-165	*ARID1B*	chr6	NM_017519	157100442	157100442	C	G	c.1379C>G	p.A460G	Missense
ICC-165	*ARID1A*	chr1	NM_006015	27023245	27023246	CA	-	c.352_353del	p.T118Gfs*281	Frameshift
ICC-167	*TP53*	chr17	NM_001126112	7577538	7577538	C	T	c.743G>A	p.R248Q	Missense
ICC-167	*KRAS*	chr12	NM_004985	25398281	25398281	C	T	c.38G>A	p.G13D	Missense
ICC-167	*KMT2D*	chr12	NM_003482	49431082	49431082	G	A	c.10057C>T	p.Q3353*	Nonsense
ICC-172	*TP53*	chr17	NM_001126112	7577574	7577574	T	C	c.707A>G	p.Y236C	Missense
ICC-172	*SMAD4*	chr18	NM_005359	48575232	48575232	T	A	c.424+2T>A	N/A	Splice site
ICC-172	*KRAS*	chr12	NM_004985	25398285	25398285	C	T	c.34G>A	p.G12S	Missense
ICC-172	*KRAS*	chr12	NM_004985						gain	Copy number gain
ICC-172	*IRS2*	chr13	NM_003749						gain	Copy number gain
ICC-172	*CDKN2B*	chr9	NM_004936						loss	Copy number loss
ICC-172	*CDKN2A*	chr9	NM_000077						loss	Copy number loss
ICC-172	*CCND2*	chr12	NM_001759						gain	Copy number gain
ICC-172	*ARID2*	chr12	NM_152641						loss	Copy number loss
ICC-172	*APC*	chr5	NM_000038	112178000	112178000	C	T	c.6709C>T	p.R2237*	Nonsense
ICC-176	*RAD50*	chr5	NM_005732	131915136	131915136	C	T	c.493C>T	p.P165S	Missense
ICC-176	*EPHA3*	chr3	NM_005233	89390976	89390976	C	A	c.1042C>A	p.P348T	Missense
ICC-178	*KDR*	chr4	NM_002253	55964435	55964435	A	G	c.2378A>G	p.N793S	Missense
ICC-178	*FGFR4*	chr5	NM_022963	176518092	176518092	T	C	c.590T>C	p.I197T	Missense

ICC-178	*FANCG*	chr9	NM_004629	35074166	35074166	C	T	c.1808C>T	p.S603F	Missense
ICC-178	*ERBB4*	chr2	NM_005235	212989553	212989553	A	G	c.158A>G	p.Y53C	Missense
ICC-178	*BAP1*	chr3	NM_004656	52436624	52436624	C	T	c.2050C>T	p.Q684*	Nonsense
ICC-184	*SUFU*	chr10	NM_016169	104263932	104263948	GCGCCCCCGGCCCCACC	-	c.37_53del	p.T13Wfs*29	Frameshift
ICC-184	*RNF43*	chr17	NM_017763	56436186	56436186	T	A	c.953-2A>T	N/A	Splice site
ICC-184	*PBRM1*	chr3	NM_018313						loss	Copy number loss
ICC-184	*NF1*	chr17	NM_001042492	29548880	29548880	C	T	c.C1654T	p.L552F	Missense
ICC-184	*MTOR*	chr1	NM_004958						loss	Copy number loss
ICC-184	*FGFR2*	chr10	NM_000141	123239418	123239418	G	C	c.C2419G	p.P807A	Missense
ICC-184	*BAP1*	chr3	NM_004656	52443761	52443761	T	A	c.38-2A>T	N/A	Splice site
ICC-185	*TSHR*	chr14	NM_000369	81609699	81609699	G	A	c.1297G>A	p.V433I	Missense
ICC-185	*ROCK2*	chr2	NM_004850	11335116	11335116	G	A	c.3484C>T	p.R1162*	Nonsense
ICC-185	*PIK3CA*	chr3	NM_006218	178936082	178936082	G	A	c.1624G>A	p.E542K	Missense
ICC-185	*PBRM1*	chr3	NM_018313	52643660	52643660	A	C	c.2236T>G	p.Y746D	Missense
ICC-185	*KRAS*	chr12	NM_004985	25398284	25398284	C	T	c.35G>A	p.G12D	Missense
ICC-185	*BLK*	chr8	NM_001715	11414325	11414325	G	T	c.931G>T	p.V311F	Missense
ICC-185	*APC*	chr5	NM_000038	112128191	112128191	C	T	c.694C>T	p.R232*	Nonsense
ICC-185	*APC*	chr5	NM_000038	112128143	112128143	C	T	c.646C>T	p.R216*	Nonsense
ICC-190	*IDH2*	chr15	NM_002168	90631839	90631839	T	A	c.514A>T	p.R172W	Missense
ICC-190	*AR*	chrX	NM_000044	66765170	66765170	A	T	c.182A>T	p.Q61L	Missense
ICC-192	*SOX2*	chr3	NM_003106	181430758	181430758	G	A	c.610G>A	p.A204T	Missense
ICC-192	*BAP1*	chr3	NM_004656	52442095	52442098	TGTG	A	c.256-5_256-2delinsT	N/A	Splice site

ICC-193	*TP53*	chr17	NM_001126112	7578395	7578395	G	-	c.535del	p.H179Mfs*68	Frameshift
ICC-193	*TNFAIP3*	chr6	NM_006290	138200324	138200324	G	A	c.1742G>A	p.R581K	Missense
ICC-193	*PIK3CA*	chr3	NM_006218	178952085	178952085	A	G	c.3140A>G	p.H1047R	Missense
ICC-193	*NRAS*	chr1	NM_002524						gain	Copy number gain
ICC-193	*HGF*	chr7	NM_000601	81374337	81374337	C	T	c.725G>A	p.R242Q	Missense
ICC-193	*ETV6*	chr12	NM_001987	11992117	11992117	G	T	c.207G>T	p.W69C	Missense
ICC-201	*FGFR2*	chr10	NM_000141-NM_014000						FGFR2-VCL	Fusion
ICC-201	*BAP1*	chr3	NM_004656	52439852	52439852	G	C	c.860C>G	p.S287*	Nonsense
ICC-217	*SRC*	chr20	NM_198291	36031711	36031711	T	G	c.1540T>G	p.Y514D	Missense
ICC-217	*SMAD4*	chr18	NM_005359	48591889	48591889	A	T	c.1052A>T	p.D351V	Missense
ICC-217	*RBM10*	chrX	NM_005676	47038857	47038857	A	-	c.864del	p.Q289Rfs*19	Frameshift
ICC-217	*NPM1*	chr5	NM_002520	170827173	170827173	T	G	c.541T>G	p.F181V	Missense
ICC-217	*KDM6A*	chrX	NM_021140	44894209	44894209	T	A	c.598T>A	p.C200S	Missense
ICC-217	*DICER1*	chr14	NM_177438	95562994	95562994	A	C	c.4263T>G	p.D1421E	Missense
ICC-217	*CBL*	chr11	NM_005188	119103162	119103168	TGCGGTT	GCGGTG	c.200_206delinsGCGGTG	p.V67Gfs*11	Frameshift
ICC-218	*PBRM1*	chr3	NM_018313	52668785	52668785	G	-	c.1137del	p.F379Lfs*25	Frameshift
ICC-218	*BAP1*	chr3	NM_004656	52441252	52441252	T	C	c.518A>G	p.Y173C	Missense
ICC-219	*TP53*	chr17	NM_001126112	7578551	7578551	A	G	c.379T＞C	p.S127P	Missense
ICC-220	*KRAS*	chr12	NM_004985	25398284	25398284	G	T	c.35G>T	p.G12V	Missense
ICC-220	*EP300*	chr22	NM_001429	41574600	41574600	A	T	c.6885A>T	p.Q2295H	Missense
ICC-220	*BARD1*	chr2	NM_000465	215593618	215593618	A	T	c.706A＞T	p.K236*	Nonsense

ICC-221	*IDH2*	chr15	NM_002168	90631840	90631840	G	A	c.515G＞A	p.R172K	Missense
ICC-221	*EPHA2*	chr1	NM_004431	16475059	16475059	C	A	c.637C＞A	p.E213*	Nonsense
ICC-222	*TP53*	chr17	NM_001126112	7578284	7578284	C	-	c.565del	p.A189Pfs*58	Frameshift
ICC-222	*ARID1A*	chr1	NM_006015	27023560	27023560	C	A	c.666C＞A	p.Y222*	Nonsense
ICC-222	*ARID2*	chr12	NM_152641	46244109	46244109	G	T	c.2203G＞T	p.G735*	Nonsense
ICC-222	*ASXL1*	chr20	NM_015338	30956903	30956903	C	T	c.229C＞T	p.R77*	Nonsense
ICC-222	*ERBB2*	chr17	NM_004448	37881329	37881329	C	G	c.2521C＞G	p.L841V	Missense
ICC-222	*ERBB3*	chr12	NM_001982	56481660	56481660	C	T	c.695C＞T	p.A232V	Missense
ICC-222	*MSH6*	chr2	NM_000179	48010538	48010538	G	A	c.166G＞A	p.G56R	Missense
ICC-223	*ALK*	chr2	NM_004304						STRN-ALK	Fusion
ICC-223	*CDKN2A*	chr9	NM_000077						loss	Copy number loss
ICC-223	*CDKN2B*	chr9	NM_004936						loss	Copy number loss
ICC-224	*TP53*	chr17	NM_001126112	7578437	7578437	C	T	c.493C>T	p.Q165*	Nonsense
ICC-224	*KEAP1*	chr19	NM_203500	10610451	10610451	C	-	c.259del	p.D87Mfs*70	Frameshift
ICC-224	*ARID2*	chr12	NM_152641	46298746	46298746	C	T	c.5393C>T	p.S1798L	Missense
ICC-224	*DICER1*	chr14	NM_177438	95596408	95596408	C	T	c.560C<T	p.R187Q	Missense
ICC-224	*GLI3*	chr7	NM_000168	42005844	42005844	T	G	c.2827T<G	p.T943P	Missense
ICC-224	*KMT2C*	chr7	NM_170606	151962184	151962184	C	A	c.1123C<A	p.V375F	Missense
ICC-224	*AMER1*	chrX	NM_152424						loss	Copy number loss
ICC-224	*NF1*	chr17	NM_001042492						loss	Copy number loss
ICC-224	*CDKN2A*	chr9	NM_000077						loss	Copy number loss
ICC-224	*CDKN2B*	chr9	NM_004936						loss	Copy number loss

ICC-225	*KRAS*	chr12	NM_004985	25380275	25380275	T	G	c.183A > C	p.Q61H	Missense
ICC-225	*KMT2D*	chr12	NM_003482	49420591	49420591	A	T	c.15158A>T	p.D5053V	Missense
ICC-225	*CDKN2A*	chr9	NM_000077						loss	Copy number loss
ICC-225	*CDKN2B*	chr9	NM_004936						loss	Copy number loss

Table S8: Impact of major clinical factors on the mutual exclusivity between Cluster 1 and Cluster 2 mutations

Clinical factors	Subtype	N	All wildtype	Cluster1 mutation	Cluster 2 mutation	co-mutation	OR	P
Gender	Male	788	333	299	141	15	0.11848004	< 0.0001
	Female	582	212	194	161	15	0.101812128	< 0.0001
Age	≥ 65	491	199	168	115	9	0.092701863	< 0.0001
	< 65	752	297	255	181	19	0.122261943	< 0.0001
Population	Eastern	924	393	390	129	12	0.093738819	< 0.0001
	Western	501	180	111	191	19	0.161313146	< 0.0001
Etiology	Infection/inflammation related	302	124	142	33	3	0.079385403	< 0.0001
	None Infection/inflammation related	568	259	164	138	7	0.080107812	< 0.0001
Stage	I/II	364	176	105	81	2	0.041387419	< 0.0001
	III/IV	505	186	206	99	14	0.127684613	< 0.0001

Table S9: List of cases with Cluster1 and Cluster2 co-mutations in the discovery cohort and validation cohorts

**ID**	**Ref**	**Cluster 1 gene mutation**	**Cluster 2 gene mutation**	**T**	**N**	**M**	**AJCC 7th Stage**
CT46	Zou SS et al	*KRAS* G12D	*IDH1* R132C	2	1	0	IVA
QX032	Sheng YY *et al*	*KRAS* G12D	*IDH1* R132G	3	0	0	III
MO_1550	Robinson DR *et al*	*KRAS* G12C	*IDH1* R132C	X	X	X	IV

38	Okamura R et al	*KRAS* G12A	*IDH1* R132C; *BAP1* loss exon 3	X	X	X	IV
201819720	Lamarca A et al	*KRAS* G13D	*IDH2* R172S	X	X	1	IVB
53	Simbolo M *et al*	*KRAS* G13D	*BAP1* K419T	3	1	0	IVA
5	Simbolo M *et al*	*KRAS* G12S	*BAP1* R57L	2	1	0	IVA
TCGA-W5-AA2O	TCGA	TP53 Y234*; *TP53* M237Gfs*20	*IDH1* R132C	1	0	0	I
MO_1369	Robinson DR *et al*	*TP53* R248Q	*IDH1* 3R132C	X	X	1	IVB
CCA_JP_31	Jusakul A *et al*	*TP53* Y220C	FGFR2-KCTD1 fusion	2b	0	0	II
MO_1039	Robinson DR *et al*	*TP53* R267W	FGFR2-BICC1 fusion	X	X	X	IV
201804639	Lamarca A et al	*TP53* C242Y	FGFR2-PAH fusion	X	X	1	IVB
CCA_TH_75	Jusakul A *et al*	*TP53* R273C	*BAP1* G128R	3	0	0	III
ICC-102	Fudan cohort	*TP53* N268Tfs*77	*BAP1* N695I	3	0	0	III
14	Simbolo M *et al*	*TP53* M246V	*BAP1* Splice site	3	0	0	III
CCA_IT_4	Jusakul A *et al*	*SMAD4* G365S	*BAP1* R540H	3	0	0	III

Table S10: The list of tested compounds and concentrations in the cell viability assay

Compounds	Concentration (μM)									
Gemcitabine	0	0.004882813	0.01953125	0.078125	0.3125	1.25	5	20	80	320
5-FU	0	0.006103516	0.024414063	0.09765625	0.390625	1.5625	6.25	25	100	400
Cisplatin	0	0.012207031	0.048828125	0.1953125	0.78125	3.125	12.5	50	200	800
Docetaxel	0	0.000305176	0.001220703	0.004882813	0.01953125	0.078125	0.3125	1.25	5	20
Dasatinib	0	0.000976563	0.00390625	0.015625	0.0625	0.25	1	4	16	64
Lapatinib	0	0.001220703	0.004882813	0.01953125	0.078125	0.3125	1.25	5	20	80
JQ1	0	0.003662109	0.014648439	0.05859375	0.234375	0.9375	3.75	15	60	240
ibet	0	0.000305176	0.001220703	0.004882813	0.01953125	0.078125	0.3125	1.25	5	20
Olaparib	0	0.006103516	0.024414063	0.09765625	0.390625	1.5625	6.25	25	100	400
Niraparib	0	0.006103516	0.024414063	0.09765625	0.390625	1.5625	6.25	25	100	400
Gefitinib	0	0.000305176	0.001220703	0.004882813	0.01953125	0.078125	0.3125	1.25	5	20
FK866	0	0.00078125	0.0015625	0.003125	0.00625	0.0125	0.025	0.05	0.1	0.2
MK1775	0	0.002441406	0.009765625	0.0390625	0.15625	0.625	2.5	10	40	160
Obatoclax	0	0.000610352	0.002441406	0.009765625	0.0390625	0.15625	0.625	2.5	10	40
Palbocilib	0	0.000305176	0.001220703	0.004882813	0.01953125	0.078125	0.3125	1.25	5	20
Sorafenib	0	0.78125	1.5625	3.125	6.25	12.5	25	50	100	200

Vorinostat	0	0.001525879	0.006103516	0.024414063	0.09765625	0.390625	1.5625	6.25	25	100
Crizotinib	0	0.001525879	0.006103516	0.024414063	0.09765625	0.390625	1.5625	6.25	25	100
GSK126	0	0.001525879	0.006103516	0.024414063	0.09765625	0.390625	1.5625	6.25	25	100

Table S11: The comparison of gene expression profiles between Cluster 1A and Cluster 2 ICC by integrating 3 independent ICC gene expression datasets

Gene symbole	logFC Cluster1A vs Cluster2			
	GSE26566	GSE89748	GSE32225	Average
S100P	3.9747099	5.130206636	2.353376246	3.819430927
KRT17	3.608404219	3.061646724	2.570120501	3.080057148
C19ORF33	3.56112877	3.399710274	1.745962188	2.902267077
SERPINB5	2.604064386	2.606215691	2.140592464	2.450290847
COL17A1	3.039328763	2.855540906	1.384705074	2.426524914
PLA2G10	2.103115904	2.774277206	1.765708078	2.214367063
GPRC5A	2.538573581	2.451334083	1.457892319	2.149266661
TFF2	1.692960923	2.810763141	1.801951415	2.101891826
CST6	2.253349214	1.89731465	2.047566749	2.066076871
TCN1	2.007428986	2.334457945	1.815883246	2.052590059
TFF1	2.057555424	1.890856654	1.975397584	1.97460322
CLIC3	1.989128178	2.463716825	1.14081763	1.864554211
ABP1	1.957679331	1.931873229	1.700145509	1.86323269
TMPRSS4	2.878558912	1.410729388	1.136211328	1.808499876
FAM3D	1.302524595	2.175016278	1.66527871	1.714273194
EVPL	1.546709842	2.165550754	1.427190601	1.713150399
CEACAM5	1.542376606	2.050367383	1.432690425	1.675144805
ERN2	1.375825888	1.064614844	2.342824057	1.594421596
VSIG2	1.928340522	1.45538244	1.265677311	1.549800091
PLAC8	1.599380564	1.892715336	1.057799808	1.516631903
FUT3	1.387359275	1.436475987	1.177126032	1.333653764
KCTD14	1.422059574	1.362757367	1.134668324	1.306495089
FXYD3	1.025030891	1.638526826	1.222592607	1.295383442
DUOXA2	1.349707287	1.023086355	1.366329963	1.246374535
FAM83E	1.587742246	1.093482012	1.004802694	1.228675651
DHRS9	1.04211449	1.149234193	1.178724714	1.123357799
APCDD1	-1.413425889	-1.00380651	-1.000748156	-1.139326852
GATS	-1.282524502	-1.000673393	-1.372092163	-1.218430019

ZNF467	-1.48057395	-1.1626845	-1.115529539	-1.25292933
OGDHL	-1.048389768	-1.511460988	-1.200939958	-1.253596905
MPP6	-1.433742594	-1.107122909	-1.506373092	-1.349079532
NPTX2	-1.566747951	-1.513629726	-1.00894911	-1.363108929
GAMT	-2.003500791	-1.117929136	-1.122546568	-1.414658832
SNRPN	-2.083778194	-1.140846283	-1.079970833	-1.434865103
PROX1	-2.014320638	-1.133409487	-1.203316297	-1.450348807
ANKRD1	-1.256940074	-1.841914363	-1.270712209	-1.456522215
SNX10	-2.01365091	-1.101543013	-1.369965389	-1.495053104
AKAP7	-1.844969705	-1.442663417	-1.20454009	-1.497391071
HKDC1	-2.330388854	-1.077712898	-1.089363372	-1.499155041
CHST13	-2.237340482	-1.37093466	-1.34514535	-1.651140164
EEF1A2	-2.25103471	-1.348029348	-1.402704364	-1.667256141
C10ORF65	-2.169816391	-1.718413514	-1.360550793	-1.749593566
LOC285016	-1.626709075	-2.456762704	-1.312353985	-1.798608588
PPARGC1A	-2.0253496	-2.102079812	-1.283614797	-1.803681403
PDGFD	-2.363775492	-1.988509874	-1.186586284	-1.84629055
TMEM27	-1.881529452	-2.149523588	-1.583264817	-1.871439286
BAAT	-2.499253575	-2.231464446	-1.009211662	-1.913309894
SCTR	-3.534875101	-1.932586196	-1.010318367	-2.159259888
FXYD2	-2.191901324	-3.083742105	-1.469041061	-2.248228163
VTCN1	-2.656176514	-2.559348339	-1.583591678	-2.266372177
DCDC2	-3.36705438	-2.306043271	-1.3598303	-2.344309317
DEFB1	-3.803511063	-2.324083757	-1.394677966	-2.507424262

References

1. Li H, Durbin R. Fast and accurate long-read alignment with Burrows-Wheeler transform. Bioinformatics. 2010;26: 589-595.

2. Li H, Handsaker B, Wysoker A, Fennell T, Ruan J, Homer N, et al. The Sequence Alignment/Map format and SAMtools. Bioinformatics. 2009;25: 2078-2079.

3. Koboldt DC, Zhang Q, Larson DE, Shen D, McLellan MD, Lin L, et al. VarScan2: somatic mutation and copy number alteration discovery in cancer by exome sequencing. Genome Res. 2012;22: 568-576.

4. Lawrence MS, Stojanov P, Polak P, Kryukov GV, Cibulskis K, Sivachenko A, et al. Mutational heterogeneity in cancer and the search for new cancer-associated genes. Nature. 2013;499: 214-218.

5. Wang P, Dong Q, Zhang C, Kuan PF, Liu Y, Jeck WR, et al. Mutations in isocitrate dehydrogenase 1 and 2 occur frequently in intrahepatic cholangiocarcinomas and share hypermethylation targets with glioblastomas. Oncogene. 2013;32: 3091-3100.
